# Transcriptional cellular responses in midgut tissue of *Aedes aegypti* larvae following intoxication with Cry11Aa toxin from *Bacillus thuringiensis*

**DOI:** 10.1186/s12864-015-2240-7

**Published:** 2015-12-09

**Authors:** Pablo Emiliano Canton, Angeles Cancino-Rodezno, Sarjeet S. Gill, Mario Soberón, Alejandra Bravo

**Affiliations:** Departamento de Microbiología, Instituto de Biotecnología, Universidad Nacional Autónoma de México, Apdo. postal 510-3, Cuernavaca, 62250 Morelos Mexico; Facultad de Ciencias, Universidad Nacional Autónoma de México, Av. Universidad 3000, Coyoacán, Distrito Federal 04510 Mexico; Cell Biology and Neuroscience Department, University of California, Riverside, Riverside, CA 92521 USA

**Keywords:** *Aedes aegypti*, *Bacillus thuringiensis* subsp *israelensis*, RNA-seq, biological control, defense response, Cry toxins

## Abstract

**Background:**

Although much is known about the mechanism of action of *Bacillus thuringiensis* Cry toxins, the target tissue cellular responses to toxin activity is less understood. Previous transcriptomic studies indicated that significant changes in gene expression occurred during intoxication. However, most of these studies were done in organisms without a sequenced and annotated reference genome. A reference genome and transcriptome is available for the mosquito *Aedes aegypti,* and its importance as a disease vector has positioned its biological control as a primary health concern. Through RNA sequencing we sought to determine the transcriptional changes observed during intoxication by Cry11Aa in *A. aegypti* and to analyze possible defense and recovery mechanisms engaged after toxin ingestion.

**Results:**

In this work the changes in the transcriptome of 4^th^ instar *A. aegypti* larvae exposed to Cry11Aa toxin for 0, 3, 6, 9, and 12 h were analyzed. A total of 1060 differentially expressed genes after toxin ingestion were identified with two bioconductoR packages: DESeq2 and EdgeR. The most important transcriptional changes were observed after 9 or 12 h of toxin exposure. GO enrichment analysis of molecular function and biological process were performed as well as Interpro protein functional domains and pBLAST analyses. Up regulated processes include vesicular trafficking, small GTPase signaling, MAPK pathways, and lipid metabolism. In contrast, down regulated functions are related to transmembrane transport, detoxification mechanisms, cell proliferation and metabolism enzymes. Validation with RT-qPCR showed large agreement with Cry11Aa intoxication since these changes were not observed with untreated larvae or larvae treated with non-toxic Cry11Aa mutants, indicating that a fully functional pore forming Cry toxin is required for the observed transcriptional responses.

**Conclusions:**

This study presents the first transcriptome of Cry intoxication response in a fully sequenced insect, and reveals possible conserved cellular processes that enable larvae to contend with Cry intoxication in the disease vector *A. aegypti*. We found some similarities of the mosquito responses to Cry11Aa toxin with previously observed responses to other Cry toxins in different insect orders and in nematodes suggesting a conserved response to pore forming toxins. Surprisingly some of these responses also correlate with transcriptional changes observed in Bti-resistant and Cry11Aa-resistant mosquito larvae.

**Electronic supplementary material:**

The online version of this article (doi:10.1186/s12864-015-2240-7) contains supplementary material, which is available to authorized users.

## Background

Vector-borne diseases remain some of human kind’s biggest health challenges that impose a significant toll on afflicted populations. The World Health Organization (WHO) estimates more than 500 million cases of malaria worldwide, transmitted by *Anopheles* mosquitoes, while *Aedes* mosquitoes transmit different viruses that cause hundreds of millions of annual infections of yellow fever, dengue, and chikungunya fever [1]. About 40 % of the human population is at risk since they live in *Aedes* inhabited locations. Importantly effective vaccines or antiviral drugs for most of these diseases do not exist, except for yellow fever. Thus efforts to control mosquito populations remain an important strategy to reduce infection rates. Biological control through the use of insecticidal proteins produced by the bacterium *Bacillus thuringiensis* (*Bt*) represents a promising alternative to control mosquitoes due to the emergence of mosquito populations that are resistant to chemical insecticides [1]. In addition, the recalcitrant nature of chemical insecticides and their non-target effects pose environmental and health disadvantages [2, 3].

*Bt* bacteria produce a wide variety of toxins, of which the pore-forming 3-domain Cry and the Cyt toxin families have been widely studied, and used in mosquito control due to their highly specific insecticidal activities [4]. In particular, the *Bt* subsp. *israelensis* (*Bti*) strain has been used in the control of *A. aegypti. Bti* mainly produces a combination of Cry11Aa, Cry4Ba, Cry4Aa and Cyt1Aa toxins as crystalline inclusions during the sporulation phase. Although *Bti* has been used for more than four decades in vector control programs worldwide, resistant populations have not been observed in the field. This lack of resistance is usually explained by the combined action of *Bti* Cry and Cyt toxins which are synergistic towards mosquito larvae [5, 6]. Also, it was shown that Cyt1Aa overcomes lab-generated resistance to individual Cry toxins [7].

The mode of action of Cry toxins has been characterized mainly in lepidopteran insect species. However, conservation of the three-dimensional structure among Cry toxins specific to different insect orders as well as conservation of cell receptors and other biochemical evidence suggest a common mechanism of action of Cry toxins in different insect orders [4]. Briefly, susceptible larvae ingest the toxin crystals, from which protoxins are solubilized and processed by midgut proteases. Activated toxin monomers bind to GPI-membrane anchored proteins, such as aminopeptidases (APN) or alkaline phosphatases (ALP). Subsequently, an additional binding event occurs with a cadherin protein, which favors further processing of monomers and induces conformational changes that generate an oligomeric toxic structure. This oligomer gains higher affinity to GPI-anchored receptors and subsequently inserts into the membrane, opening a non-selective pore [8]. Osmotic shock mediated by this pore is thought to be the main cause of cell death [8]. For the mosquitocidal Cry toxins produced by *Bti*, similar Cry-receptor molecules have been identified in different mosquito species like cadherins, APN and ALP indicating a conserved mode of action in mosquitoes (reviewed in [9]). In addition an α-amylase was shown to bind Cry4Ba and Cry11Aa toxins and proposed as toxin receptor in *Anopheles albimanus* [10].

Although much is known about the molecular interactions of Cry toxins with its membrane receptors, much less is known about the cellular responses after Cry toxin action. Bacterial pore-forming toxins have been studied in a number of systems including mammalian-cells, where toxin-receptor binding or damage to the membrane elicits a diverse array of responses depending on the toxin dose, cell type, and duration of exposure. Mechanisms such as apoptosis and pyroptosis have been observed, as well as lipid synthesis, membrane repair, endo/exocytosis and activation of autophagy [11–15]. Shedding of membrane proteins [16] and membrane vesicle blebbing are other observed effects [13]. It has been reported that phosphorylation of MAP kinases is frequently observed during the responses elicited in different cells to several pore-forming toxins. Specifically JNK kinase was activated by streptolysin-O [17] and Cry5B [18]; ERK 1/2 by anthralysin-O [16]; and p38 MAPkinase by streptolysin-O [17], anthralysin-O [16], α-toxin [14, 19], pneumolysin [20, 21], β-toxin [22], aerolysin [23], Cry5B [18], Cry3Aa [24], Cry1Ab and Cry11Aa toxins [25].

The targets of these MAP kinases include transcriptional factors implying that changes in gene expression can be expected during cellular responses to damage induced by a pore-forming toxin. Previous studies have indicated that this is indeed the case for Cry toxins in insects and nematodes. For example, transcript changes in response to Cry toxins have been reported in lepidopteran larvae, such as *Spodoptera frugiperda* [26], *Choristoneura fumiferana* [27], and *Lymatria dispar* [28], as well as in the coleopteran *Tenebrio molitor* [24]. However, limitations on the availability of a reference genome restricted the amount of information obtained, and phylogenetic distances between these insects may influence the response mechanisms to Cry toxins. Therefore, any new information about transcriptome changes after Cry toxin exposure in larvae brings us closer to unraveling a shared response in insects against these important toxins. In this paper we present a transcriptomic study of the response of the mosquito *A. aegypti* in the midgut tissue after intoxication with Cry11Aa.

## Results

### High-throughput Illumina sequencing

*Bti* produces several mosquitocidal toxins. For this study, we chose Cry11Aa since it shows high toxicity to *A. aegypti* larvae with an LC_50_ value of 450.9 ng/ml (confidence limits, 350.5–557.1 ng/ml). Also, because it was previously shown that Cry11Aa affects MAPK p38 phosphorylation during intoxication of *A. aegypti* larvae [25]. In this work we analyzed the transcriptome of 4th instar *A. aegypti* larvae exposed to Cry11Aa toxin (500 ng/ml) for different times. Control larvae at the shorter and longer incubation times in the absence of toxin were also included in our analyses. The Illumina reads obtained were checked for overrepresented sequences or artifacts, but none were found. All samples produced around 20–60 million reads with good quality. Reads were aligned to the *A. aegypti* transcripts as indicated in methods. Additional file 1: Table S1 shows the alignment rates for all samples. Above 65 % of all reads were aligned to *A. aegypti* transcripts uniquely. In addition 5 % to 12 % of total reads aligned to *A. aegypti* genome but not to the transcriptome, which could represent new genes or new splicing alternatives that have not been characterized before (data not shown).

### Differential expression analysis

We used two different algorithms, DESeq2 and EdgeR [29, 30], to identify genes with significant differential expression. Table 1 shows that DESeq2 reported more genes as differentially expressed genes (DEG) than EdgeR for every exposure time except at 3 h. Also, most of the genes detected by EdgeR were also detected by DESeq2, with the subset of genes defined as DEG only by EdgeR being small overall. We observed that as the exposure time to Cry11Aa increases, the number of DEGs also increases, where the number of positively regulated genes is larger than the number of negatively regulated ones.

**Table 1:** Number of genes reported in differential expression analysis by DESeq2 (P-adj < 0.05) and EdgeR (FDR < 0.05)

Condition		EdgeR	DESeq2	Common	Only EdgeR	Only DESeq2
3 h	all	10	5	4	6	1
	Up regulated	3	1	1	2	0
	Down regulated	7	4	3	4	1
6 h	all	33	340	27	6	313
	Up regulated	24	245	23	1	222
	Down regulated	9	95	4	5	91
9 h	all	151	458	145	6	313
	Up regulated	105	273	104	1	169
	Down regulated	46	185	41	5	144
12 h	all	1059	1980	1038	21	942
	Up regulated	623	1123	613	10	510
	Down regulated	436	857	425	11	432
	all	39	16	13	26	3
12 h no toxin	Up regulated	20	7	4	16	3
	Down regulated	19	9	9	10	0

Across all timepoints, a total of unique 1060 DEG common to both packages were used for further analyses (Additional file 2: Table S2). As control we analyzed the DEG of non-toxin exposed larvae after incubation for 0 or 12 h by RNAseq in triplicate. In contrast to the 1038 common DEG seen at 12 h of Cry11Aa exposure, only 13 DEG were found in the control non-toxin exposed larvae after 12 h, with nine genes down regulated and four up regulated genes (Additional file 3: Table S3). Of these genes found in control larvae, only 6 genes are also found in the 1060 DEGs found for all toxin exposure conditions. Two of them, AAEL003841 and AAEL003816, correspond to a defensin antimicrobial peptide and a close homolog, suggesting that this process is not related to the response to Cry11Aa toxicity.

### Biological Process and Molecular Function GO enrichment analysis

**Fig. 1:**
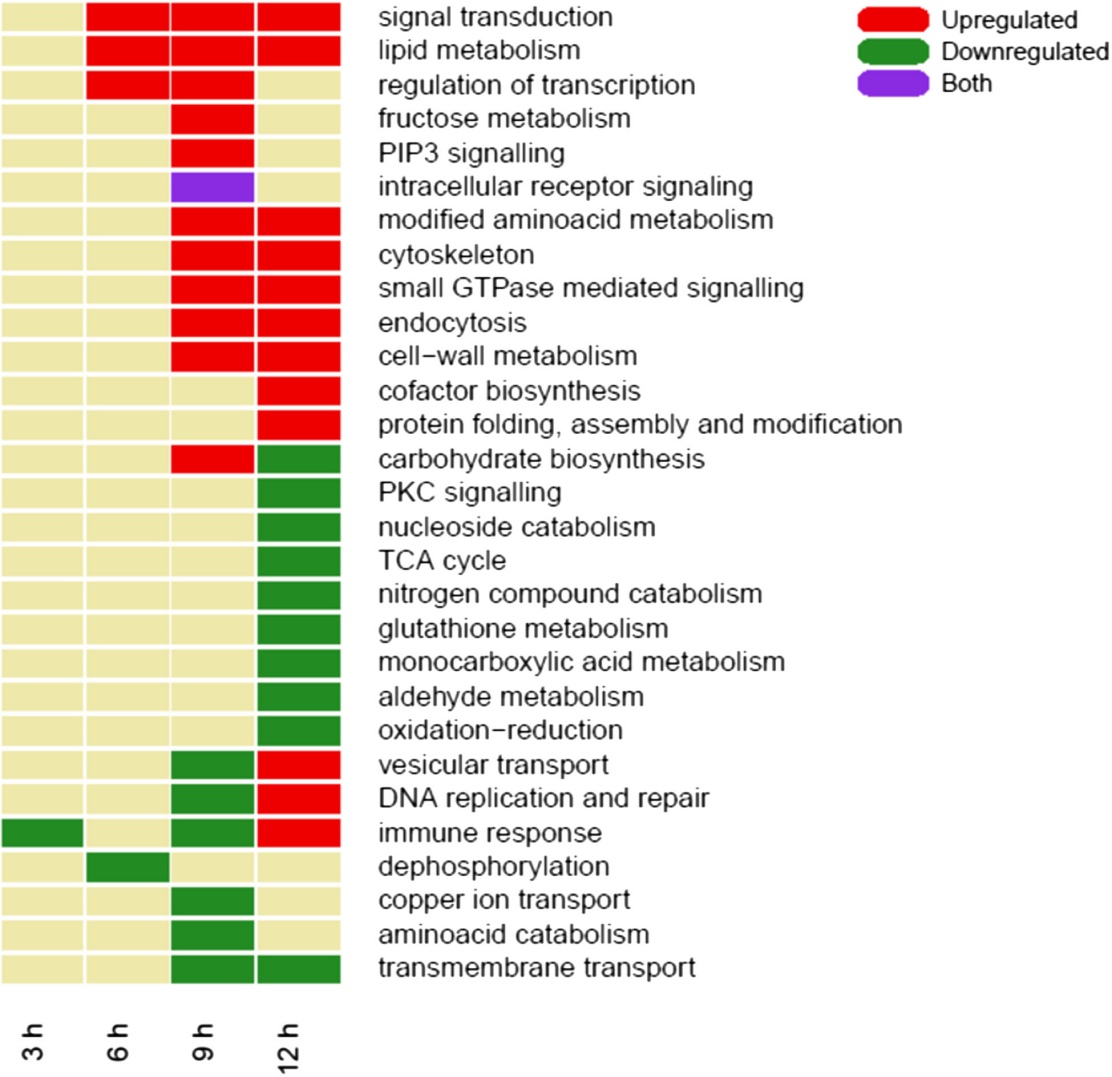
Enrichment analysis for biological process GO terms. For each experimental condition, terms reported as enriched by TopGO on differentially expressed genes (as described in Methods) are colored green if present in down regulated genes, red if found in up regulated genes, and purple if found in both sets of genes. Some related GO terms are shown as one for simplicity

We performed a GO enrichment analysis of biological process annotations on the subset of DEG that were common in the DESeq2 and EdgeR results. Figure 1 represents these overrepresented terms for each toxin exposure time. Some related GO terms are represented as a single term for simplicity. It is not surprising to find almost no enriched terms at 3 h of Cry11Aa exposure since few genes were detected as differentially expressed under this condition. However, there is a clear increase in this number as exposure time increases, as is expected from the number of DEG for each condition. As exposure time to Cry11Aa increases there is a progressive enrichment of terms related to cellular remodeling and component shuttling in positively regulated genes: cytoskeleton, endocytosis, lipid metabolism, cell-wall metabolism and vesicular transport.

In addition, our data suggest that small GTPase and PIP_3_ signaling contribute to a general response to the membrane damage induced by Cry11Aa. It was previously reported that signaling through Rho-GTPase participates in the regulation of vesicular traffic [31], indicating that this response could have a role in the removal and repair of damaged cell membranes after Cry toxin intoxication. Protein integrity is also conserved through the up regulation of chaperones Hsp70 and Hsp20, and this is reflected in the enrichment of the terms for protein folding, complex assembly and post-translational modification.

Among the terms that were down regulated we found some transmembrane transport functions such as those involved in sulfate and carbohydrate transport. Other terms that were highly repressed include those related to catabolic pathways, cell replication, and PKC signaling. The GO term for immune response was initially negatively regulated, but as intoxication proceeds, immune response showed a positive regulation. Importantly our studies were done with purified Cry11Aa-crystals by sucrose gradient centrifugation, limiting the number of bacteria or bacterial wall components present in the crystal samples used for intoxication. These data may represent a survival response of larvae that attempt to defend against possible future bacterial intrusions.

**Fig. 2:**
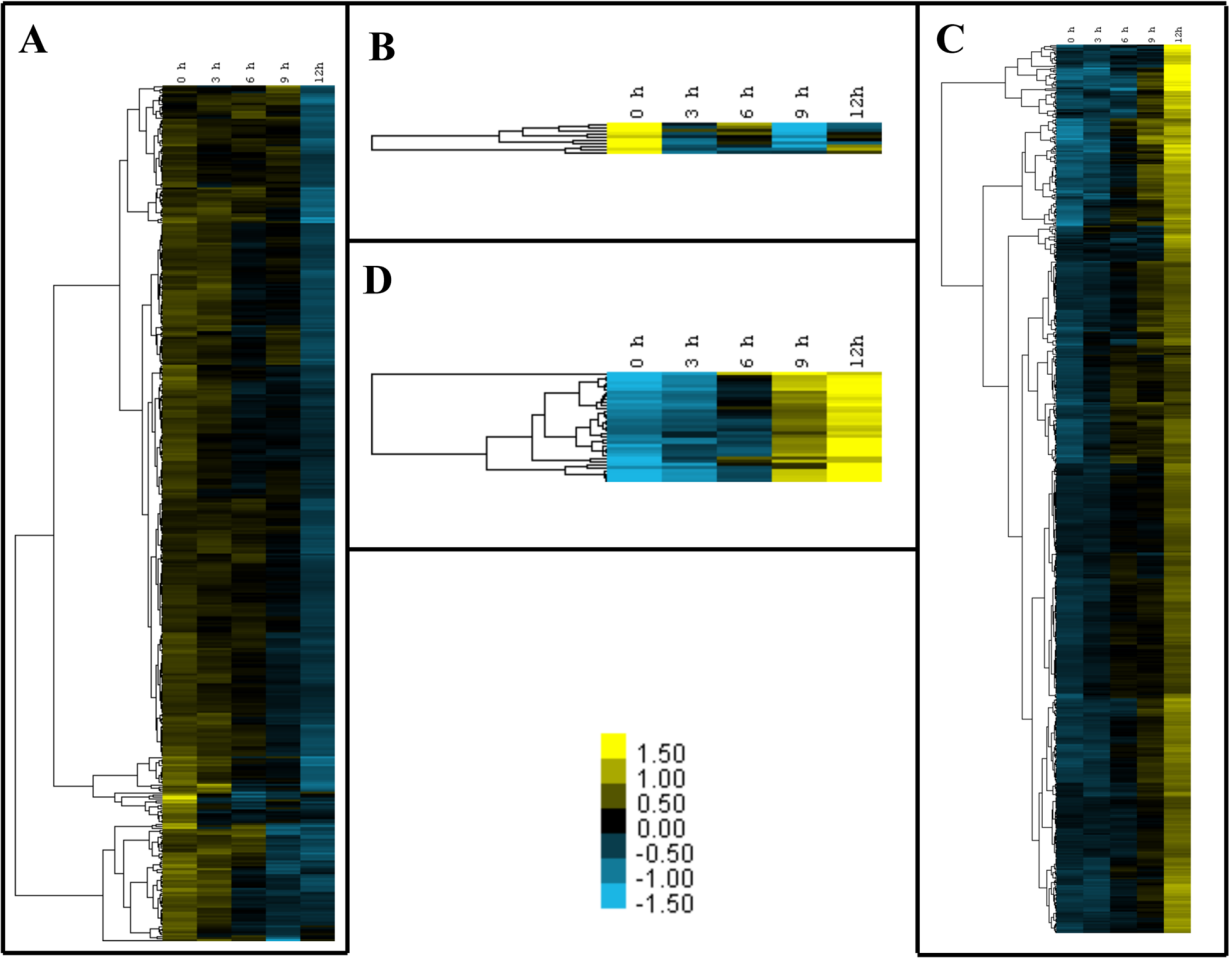
Clustering of differentially expressed genes by expression profile. Log_2_ fold expression profiles from 0 to 12 h of Cry11Aa exposure for all genes determined as differentially expressed in at least one experimental condition and clustered using DEseq2 and Cluster 3.0 as described in Methods. We considered strong response when difference between maximal and minimal expression values are over 2 fold and moderate response when difference between maximal and minimal expression values are equal or lower than 2 fold. Cluster **a**, represents genes with moderate down regulation; cluster **b**, strong down regulation; cluster **c**, moderate up regulation and cluster **d**, strong up regulation

To compare all conditions at the same time instead of through individual enrichment, we performed a clustering analysis on the log_2_ fold values of the 1060 common DEG as described in Methods, and determined those genes with an overall similar expression profile throughout the time of toxin exposure. Four different clusters could be observed (Fig. 2). Clusters A and B show groups of genes with down regulation patterns. Genes with strong down regulation (when difference between maximal and minimal expression values are over 2 fold) are grouped in cluster B while moderately down regulated genes (when the difference between maximal and minimal expression values are equal or lower than 2 fold) are in cluster A. Up regulated genes were grouped in clusters C and D. Cluster C showed those with moderate up regulation and cluster D those with strong up regulation. Additional file 2: Table S2 lists all genes that are present in each cluster. The only exception was AAEL008767 gene, corresponding to a serine protease, which showed a unique expression profile with a peak induction at 3 h. With these separate clusters we performed again a GO enrichment analysis of biological process terms as described above and results are presented in Table 2. In general the GO enrichment results of these clusters resemble the GO terms shown in Fig. 1. We observed moderate down regulation of terms involved in oxidation processes, TCA enzymes, transmembrane transport, aldehyde, monocarboxylic acid and glutathione metabolisms, catabolic process of nitrogen compounds, heterocycle and nucleosides, and PKC signaling. The strongest down regulation at 9 h were found in bacterial defense terms. The cluster with moderate up regulation is enriched with terms of vesicular transport, lipid biosynthetic process, protein complex formation and protein folding, cellular morphogenesis, cytoskeleton binding, initiation of DNA replication, integrin and cell-cell signaling, as well as MAPK and small GTPase signaling. The strong up regulation cluster was enriched for actin nucleation, lipid catabolism, calcium ion transport, and Rho GTPase regulation (Table 2).

**Table 2:** TopGO enrichment of biological process terms in differentially expressed gene clusters

Cluster	GO.ID	Term	Annotated	Significant	Expected	ClassicFisher
A. Moderate down regulated	GO:0055114	oxidation-reduction process	769	75	29.4	2.10E-13
	GO:0008272	sulfate transport	10	5	0.38	1.70E-05
	GO:0006081	cellular aldehyde metabolic process	9	3	0.34	0.0039
	GO:0032787	monocarboxylic acid metabolic process	70	8	2.68	0.0067
	GO:0006749	glutathione metabolic process	14	3	0.54	0.0147
	GO:0005978	glycogen biosynthetic process	6	2	0.23	0.0197
	GO:1901700	response to oxygen-containing compound	6	2	0.23	0.0197
	GO:0044270	cellular nitrogen compound catabolic process	385	17	14.72	0.0235
	GO:0046700	heterocycle catabolic process	386	17	14.76	0.0264
	GO:0006099	tricarboxylic acid cycle	18	3	0.69	0.0295
	GO:0044262	cellular carbohydrate metabolic process	41	6	1.57	0.0318
	GO:1901361	organic cyclic compound catabolic process	388	17	14.83	0.0326
	GO:0006730	one-carbon metabolic process	8	2	0.31	0.035
	GO:1901292	nucleoside phosphate catabolic process	357	13	13.65	0.0511
	GO:0055085	transmembrane transport	576	34	22.02	0.0619
	GO:0043648	dicarboxylic acid metabolic process	11	2	0.42	0.0638
	GO:0007205	protein kinase C-activating G-protein coupled receptor signaling pathway	11	2	0.42	0.0638
	GO:0044106	cellular amine metabolic process	20	3	0.76	0.0743
	GO:0006979	response to oxidative stress	28	3	1.07	0.0897
B. Strong down regulated	GO:0006952	defense response	14	2	0.01	0.0021
	GO:1902476	chloride transmembrane transport	6	1	0	0.0034
	GO:0042742	defense response to bacterium	9	1	0.01	0.0051
C. Moderate up regulated	GO:0071702	organic substance transport	266	27	10.92	2.10E-05
	GO:0008610	lipid biosynthetic process	103	13	4.23	0.00025
	GO:0016192	vesicle-mediated transport	114	15	4.68	0.00091
	GO:0043623	cellular protein complex assembly	32	7	1.31	0.00103
	GO:0042398	cellular modified amino acid biosynthetic process	20	5	0.82	0.00105
	GO:0007229	integrin-mediated signaling pathway	8	3	0.33	0.00329
	GO:0006195	purine nucleotide catabolic process	341	24	14	0.00404
	GO:0006955	immune response	17	4	0.7	0.00433
	GO:0042742	defense response to bacterium	9	3	0.37	0.00478
	GO:0032940	secretion by cell	18	4	0.74	0.00539
	GO:0009207	purine ribonucleoside triphosphate catabolic process	340	24	13.96	0.00684
	GO:0006777	Mo-molybdopterin cofactor biosynthetic process	11	3	0.45	0.00884
	GO:0030029	actin filament-based process	35	6	1.44	0.00941
	GO:0010466	negative regulation of peptidase activity	24	4	0.99	0.01544
	GO:0006108	malate metabolic process	5	2	0.21	0.01547
	GO:0007267	cell-cell signaling	14	3	0.57	0.0178
	GO:0034613	cellular protein localization	136	11	5.58	0.01829
	GO:0007264	small GTPase mediated signal transduction	189	12	7.76	0.02202
	GO:0006470	protein dephosphorylation	54	6	2.22	0.02244
	GO:0019751	polyol metabolic process	6	2	0.25	0.02258
	GO:1901617	organic hydroxy compound biosynthetic process	6	2	0.25	0.02258
	GO:0016051	carbohydrate biosynthetic process	26	4	1.07	0.04051
	GO:0006457	protein folding	80	7	3.28	0.04524
	GO:0007017	microtubule-based process	81	7	3.33	0.04786
	GO:0046130	purine ribonucleoside catabolic process	340	24	13.96	0.04798
	GO:0048285	organelle fission	34	4	1.4	0.04903
	GO:0000165	MAPK cascade	9	2	0.37	0.04995
	GO:0006270	DNA replication initiation	9	2	0.37	0.04995
	GO:0033014	tetrapyrrole biosynthetic process	10	2	0.41	0.06079
	GO:0046939	nucleotide phosphorylation	11	2	0.45	0.07233
	GO:0016998	cell wall macromolecule catabolic process	11	2	0.45	0.07233
	GO:0000902	cell morphogenesis	11	2	0.45	0.07233
	GO:0044262	cellular carbohydrate metabolic process	41	4	1.68	0.08597
	GO:0006879	cellular iron ion homeostasis	13	2	0.53	0.09725
	GO:0006826	iron ion transport	13	2	0.53	0.09725
	GO:0046854	phosphatidylinositol phosphorylation	13	2	0.53	0.09725
D. Strong up regulated	GO:0016042	lipid catabolic process	51	2	0.11	0.005
	GO:0034314	Arp2/3 complex-mediated actin nucleation	7	1	0.01	0.015
	GO:0070588	calcium ion transmembrane transport	17	1	0.04	0.035
	GO:0032319	regulation of Rho GTPase activity	24	1	0.05	0.05

**Fig. 3:**
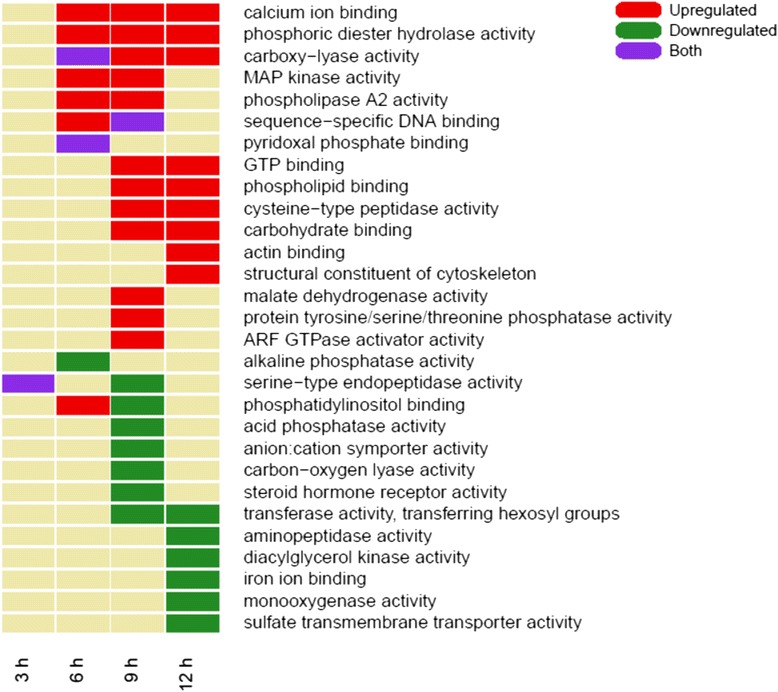
Molecular functions of genes differentially expressed. For each experimental condition, genes were categorized into different categories as reported by TopGO and enrichment on differentially expressed genes (as described in Methods) are colored green indicating down regulated genes, red indicating up regulated genes, and purple if found in both sets of genes

Of the 17,478 predicted genes for *A. aegypti,* only about a third are annotated with a biological process GO term. Therefore, there are many DEG for which a role in biological process could not be assigned in our GO analysis shown in Fig. 1. We performed a similar enrichment analysis but using a different GO ontology, that was focused to the molecular function instead of the biological process. Figure 3 shows this GO molecular function enrichment analysis. At 3 h the only enriched term for both up and down regulated genes is for serine-type endopeptidase activity. This could be an artifact due to the small number of DEG detected for this condition. An alternate explanation is that the serine protease response may be down-regulated when the insects stop feeding as a response to intoxication, and other serine proteases may be up-regulated due to a starvation response. From 6 h onwards, up regulated genes are enriched in terms related to phospholipid interactions, such as lipid binding or phospholipase A2 activity. We also observed enrichment of carboxylyase activity in both up and down regulated genes at 6 h, but this activity is only further enriched at 9 and 12 h of toxin exposure. Here again, MAPK activity and terms related to both ARF and Rho GTPase activity are enriched in up regulated genes from 6 h and forward, supporting a possible role these two signaling pathways in the response to Cry intoxication. Probably related to the activity of these proteins, nucleotide binding and phosphotransferase terms are also enriched at 12 h. The scavenger receptor term in these exposure times may point to the recycling of membrane proteins. In the down regulated genes at 9 and 12 h we observed enrichment of terms related to ion binding and transport as well as terms related to oxidation-reduction processes, although other different terms involved in oxidation-reduction processes were observed in the group of up regulated genes. Interestingly, the ALP and APN activity terms were down regulated at 6 and 12 h, respectively. Proteins in both these families function as receptors for Cry toxins [4]. However, these genes that are down regulated do not correspond to the ALP or APN proteins that have been shown to bind Cry11Aa toxin in mosquito epithelial cells [32].

### Identification of differential expressed genes that were not annotated

We found 676 DEG that were not annotated with GO biological process terms. To extend our analysis, these 676 genes were analyzed to obtain their Interpro protein functional domains [33] as described in Methods. Table 3 shows the representative domains that are distributed in the different sets of genes, and Additional file 4: Table S4 shows a list with all domains found with frequency ≥ 90^th^ percentile (0.9). For the up regulated genes at 6 h of toxin exposure we detected protein domains related to carboxylases, and these are also up regulated at 9 and 12 h of Cry11Aa intoxication. At these times we also found further evidence of genes that support the results of up regulated genes identified by GO enrichment of biological functions that were presented in Fig. 1 such as chaperones involved in protein folding and complex assembly, annexin involved in cytoskeleton and EF-Hand domains involved in Ca^2+^ binding. Regarding DEG that were down regulated we found domains of zinc fingers, type-C lectins, and ATPse domains of replication proteins. ABC transporter domains were also found to be down regulated.

**Table 3:** Representative Interpro domains identified on differential expressed genes without biological process GO annotation

Condition	InteproID	Percentile	Description
6 h down regulated	IPR007588	1	Zync finger, FLYWCH-type
6 h up regulated	IPR002018	0.94	Carboxylesterase, typeB
	IPR029058	1	Alpha/Beta hydrolase fold
9 h down regulated	IPR007087	0.9	Zync finger, C2H2
9 h up regulated	IPR013126	0.966	Heat shock protein 70 family
	IPR029058	0.955	Alpha/Beta hydrolase fold
	IPR002018	0.944	Carboxylesterase, typeB
	IPR013783	0.933	Immunoglobulin-like fold
12 h down regulated	IPR007087	0.996	Zync finger, C2H2
	IPR011009	0.977	Protein kinase-like domain
	IPR013026	0.936	Tetratricopeptide repeat-containing domain
	IPR013525	0.936	ABC-2 type transporter
12 h up regulated	IPR029058	1	Alpha/Beta hydrolase fold
	IPR008978	0.997	HSP20-like chaperone
	IPR027417	0.988	P-loop containing nucleoside triphoshpate hydrolase
	IPR013126	0.969	Heat shock protein 70 family
	IPR002048	0.969	EF-hand domain
	IPR002018	0.958	Carboxylesterase, typeB
	IPR001849	0.947	Pleckstrin homology domain
	IPR015880	0.947	Zync finger C2H2-like
	IPR002110	0.925	Ankyrin repeat
	IPR001452	0.925	SH3 domain
	IPR008985	0.925	Concanavalin A-like lectin/glucanases superfamily
	IPR018502	0.925	Annexin repeat

After performing the Interpro analysis we realized that 154 DEG still did not have any identifiable protein domain. These 154 genes were further analyzed by performing pBLAST analysis on the NR database [34], though few proteins were found to have significant homology to other gene targets. Table 4 shows the identified genes. We found homologs of phospholipase A2 family, which supports the importance of activating this process in response to Cry11Aa intoxication. Again, a component of Ras GTPase signaling such as the homolog of Ras guanine exchange factor was identified in genes induced after Cry11Aa exposure. Among the down regulated genes we found some antimicrobial peptides such as CEC-A and gambicin, and also an ADAM metalloprotease.

**Table 4:** Homologs to differentially expressed genes without biological process GO annotation found by pBLAST analysis

Condition	Gene ID	Coverage	*E*-value	Identity	Homolog ID	Protein	Species
9 h Down regulated	AAEL013895	90 %	1e-32	53 %	XP_001868961.1	14.5 kDa salivary protein	Cq
	AAEL009985	94 %	2e-59	65 %	XP_001868961.1	14.5 kDa salivary protein	Cq
	AAEL000777	83 %	3e-15	60 %	ABU80079.1	CEC-A	Ag
	AAEL004522	98 %	8e-29	65 %	ACA05605.1	gambicin	Am
9 h up regulated	AAEL007206	92 %	2e-22	48 %	XP_004526025.1	^a^rhytmically expressed gene 5 protein-like	Cc
	AAEL000811	91 %	1e-127	48 %	ETN67412.1	methusalah	Ad
	AAEL002963	32 %	5e-09	57 %	XP_008549507.1	^a^ras guanine nucleotide exchange factor L isoform X8	Md
12 h Down regulated	AAEL006228	98 %	9e-102	45 %	XP_001846856.1	PPARgamma constitutive conactivator 1	Cq
	AAEL012622	98 %	5e-108	68 %	XP_001862944.1	ADAM metalloprotease	Cq
	AAEL007780	96 %	2e-107	57 %	AAV90698.1	putative salivary secreted protein 275	Aa
	AAEL009985	94 %	2e-59	65 %	XP_001868961.1	14.5 kDa salivary protein	Cq
	AAEL006346	81 %	5e-62	55 %	XP_005177200.1	^a^BAG family molecular chaperone regulator 2-like isoform X1	Mud
	AAEL003594	98 %	1e-169	61 %	XP_001862361.1	microtubule binding protein D-CLIP-190	Cq
	AAEL013356	30 %	8e-62	62 %	XP_003401795.1	^a^eukaryotic initiation factor 4A-like isoform 2	Bi
12 h Up regulated	AAEL006493	98 %	7e-11	49 %	AAV90653.1	putative salivary secreted peptide	Aa
	AAEL005874	100 %	4e-63	73 %	XP_003707525.1	^a^E3 ubiquitin-protein ligase AMFR-like	Mr
	AAEL006493	98 %	7e-11	49 %	AAV90653.1	putative salivary secreted peptide	Aa
	AAEL013220	75 %	3e-62	72 %	NP_648500.1	crimpled	Dm
	AAEL011867	92 %	0	40 %	XP_003395464.1	^a^muscle M-line assembly protein unc-89 isoform X3	Apm
	AAEL014310	86 %	8e-34	79 %	NP_648366.2	calcium-independent phospholipase A2 VIA, isoform B	Dm
	AAEL007206	92 %	2e-22	48 %	XP_004526025.1	^a^rhythmically expressed gene 5 protein	Cc
	AAEL001576	94 %	1e-31	83 %	ETN61409.1	cysteine string protein	Ad
	AAEL003567	99 %	6e-43	49 %	AAV90653.1	putative salivary secreted peptide	Aa
	AAEL002578	97 %	1e-59	65 %	NP_649184.1	snakeskin, isoform A	Dm
	AAEL000811	91 %	1e-127	48 %	ETN67412.1	methusalah	Ad
	AAEL005428	98 %	2e-32	51 %	ABV44740.1	13.6 kDa midgut protein	Pp
	AAEL002963	32 %	5e-09	57 %	XP_008549503.1	^a^myb-like protein J isoform X4	Md

*Aa* Aedes albopictus, *Ad* Anopheles darlingi, *Ag* Anopheles gambiae, *Am* Anopheles melas, *Apm* Apis mellifera, *Bi* Bombus impatiens, *Cc* Ceratitis capitata, *Cq* Culex quinquefasciatus, *Dm* Drosophila melanogaster, *Md* Microplitis demolitor, *Mud* Musca domestica, *Mr* Megachile roduntata, *Pp* Phlebotomus papatasi

^a^indicates protein classified as “PREDICTED”

### Validation through RT-qPCR

To corroborate the RNAseq data, we selected 14 genes from the common DEG to validate their expression by RT-qPCR at the same time-points analyzed by RNAseq. Among these genes, two genes related to MAPK signaling: JNK (AAEL008622) and Ets domain containing protein (AAEL006533) were analyzed. The closest homolog for AAEL006533 is Elk-1 transcription factor, a known downstream target of MAPK-p38. Therefore its positive regulation may confirm the activation of this pathway, as the transcript for p38 itself does not show differential expression, which is consistent with previous observations [25]. According to Vectorbase annotation *A. aegypti* has to two isoforms of JNK, where AAEL008622 shows higher homology to human JNK2. We also selected one gene for phospholipase A2 activity (AAEL001523), two genes for vesicular traffic such as lipoma preferred partner and sorting nexin (AAEL007704, AAEL005655), two metalloproteases (AAEL002661, AAEL005992), three genes for transporter proteins related to transport such as monocarboxylate, sucrose and sugar transporter proteins (AAEL008347, AAEL003633, AAEL012903), a CoA-Ligase (AAEL014664) and an ALP (AAEL003905). We also analyzed the gene for the antimicrobial peptide defensin (AAEL003841) and a hypothetical protein with high homology to defensin (AAEL003816) that were also observed in control larvae.

**Fig. 4:**
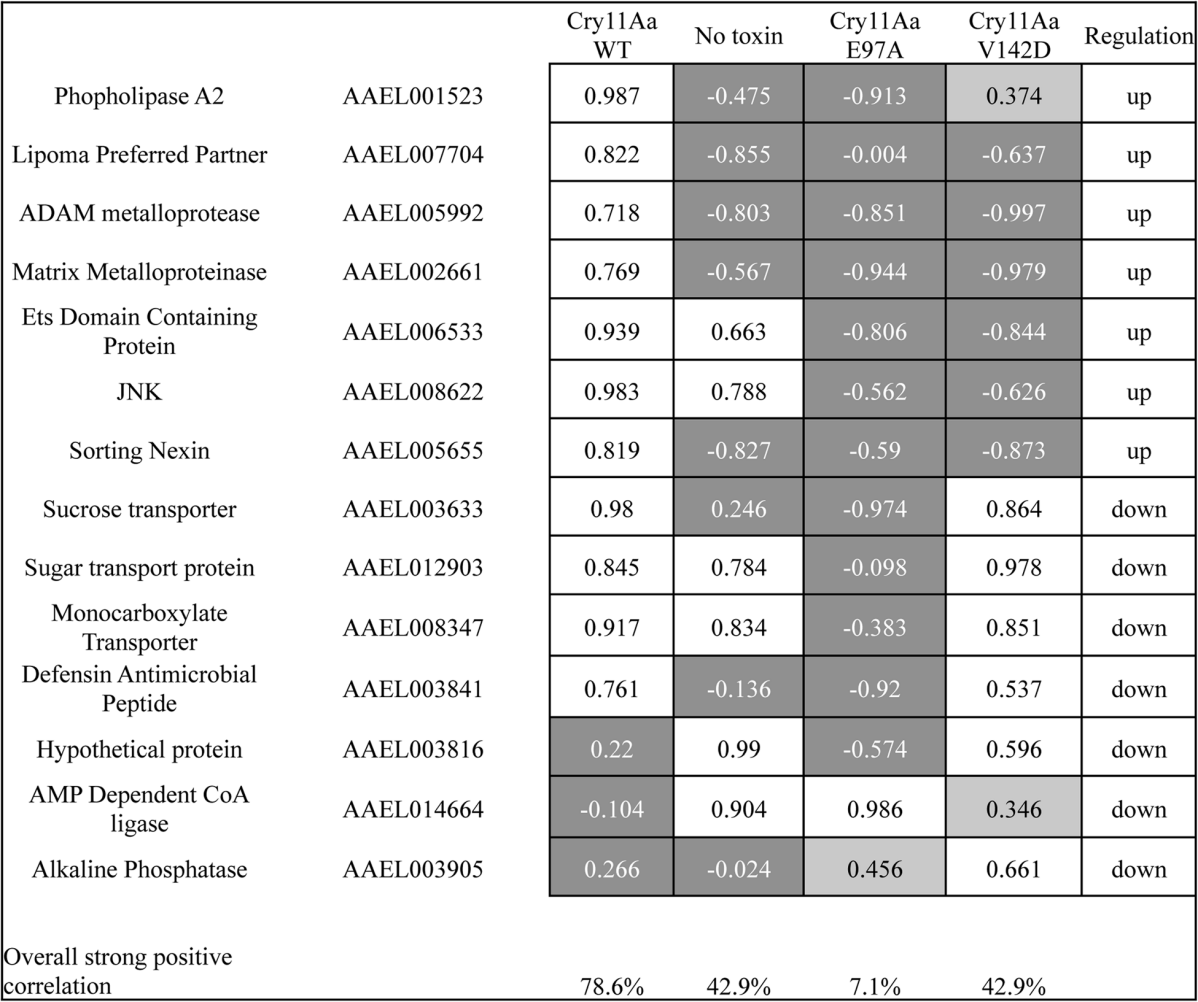
RNAseq and RT-qPCR correlation. Transcript abundance ratios were determined for each gene by SYBR Green RT-qPCR on a biological replicate of a 12 h Cry11Aa exposure curve, a control curve of non-toxin exposed *A. aegypti* larvae, or larvae exposed to non-lethal Cry11Aa mutants. Spearman correlation was determined between corresponding RNAseq values and log_2_ transformed abundance ratios. Strong positive correlations (above 0.5) are colored in white, weak positive correlation (between 0.3 and 0.5) are colored in light gray, and low, negative or no correlation values are colored in dark gray. Genes are indicated to be up regulated or down regulated as determined by RNAseq analysis. The percentage of genes with strong positive correlation between each curve and RNAseq data is shown

Transcript abundance ratios were determined at all times after toxin exposure. As controls we analyzed the transcript abundance ratios of control larvae without toxin exposure for different times or after exposure to the non-lethal Cry11Aa mutants E97A and V142D. The mutant E97A is not toxic since it is unable to form toxin oligomers required for pore formation [35], while the mutant V142D is able to oligomerize but it does not insert into the lipid bilayer of the cell membrane [36]. Additional file 5: Figure S1 shows the relative expression of all 14 genes at different times of the four conditions analyzed (after Cry11Aa toxin ingestion, control without-toxin, or with non-toxic mutants Cry11Aa-E97A and Cry11AaV142D). In order to compare data of Additional file 5: Figure S1 with our results of transcription expression by RNAseq a correlation coefficient for each target gene between the RT-qPCR and their corresponding RNAseq data is presented in Fig. 4. Most target genes show strong positive correlation with the data of the biological replicate of the LC_50_ curve, but not strong positive correlation with data of either non-exposed larvae or non-lethal Cry11Aa exposed larvae. Overall correlation of this set of genes is 79 % with the Cry11Aa LC_50_ biological replicate, which is more than the closest value of 43 % with Cry11Aa V142D data. These results indicate that our RNAseq data and observations have good experimental validation. Results also indicate that the expression values observed are more likely a response to the successful formation of toxin pores in the membranes of the intestinal epithelial cells than to the presence of the toxin, and that changes observed are not merely due to the growth or circadian regulation of genes in the mosquito larvae during the time of the experiment.

As an additional validation analysis of RNAseq data we determined the changes in expression of JNK protein by western blot analysis (Additional file 6: Figure S2). RNAseq data showed that JNK (AAEL008622) was up regulated after 9 and 12 h of Cry11Aa exposure. Our analysis indicated a direct correlation of the expression of JNK protein with JNK (AAEL008622) gene expression after 9 and 12 h of Cry11Aa exposure (Additional file 6: Figure S2).

## Discussion

Here we show the analysis of the transcriptional response of *A. aegypti* midgut epithelial cells after the first 12 h of Cry11Aa exposure. With intoxication, cellular responses are likely diverse in an attempt by the host to avert toxicity. We took care not to include severely intoxicated or dead larvae in the sequenced samples. That way we could say that the larvae analyzed were mildy intoxicated and maybe able to defend from the toxin up to the point when they were sampled. We found that the longer the exposure the more genes were differentially expressed. This is consistent with previous microarray data obtained (Gill SS, data not published), where high doses of toxin generated a widespread repression of genes while an LD_50_ or lower showed a general profile favoring positive differential expression. Also in the coleopteran pest *Tenebrio molitor* the timepoint where the most differential gene expression was observed was after 12 h incubation with Cry3Aa toxin [24].

Due to the experimental bioassays conditions we cannot be sure that all individual larvae consume Cry toxin at the same rate and in the same amount. It is possible that in the shorter exposure times not all larvae had fed Cry11Aa crystals, which could explain why the overall transcriptional response is milder than at longer exposure times. However, at 9 and 12 h of Cry11Aa toxin exposure we saw a strong cellular response and a similar profile of enriched biological functions. The control larvae at 0 and 12 h in absence of toxin administration showed a limited number of DEG, indicating that the responses observed here correspond to transcriptional changes due to Cry11Aa toxin action.

### Up-regulated responses

Our results indicate that exposure to an LC_50_ of Cry11Aa induces the expression of genes related to vesicular traffic, protein folding, lipid metabolism and cell membrane metabolism. Chaperone, phospholipase A2 and carboxylesterase activities were also found among the induced functions at longer intoxication times. Small GTPases of the Ras superfamily are known regulators of cell junctions, cytoskeleton and vesicular traffic in cells [37, 38]. In particular, they play key roles in maintaining epithelial homeostasis [38], as observed in mammalian intestinal epithelium [31] and also insect epithelium [39]. Here we observed that components of the ARF and Rab family are induced, as well as many nucleotide exchangers and regulators of Rho signaling, especially at 12 h of exposure. These data, coupled with the induction of genes related to actin nucleation and cell junction establishment, point to an active process of epithelial tissue homeostasis. We also found homologs of *Drosophila melanogaster* crimpled and snakeskin genes induced at 12 h; both have a role in maintenance of cell junctions [40, 41]. It is important to mention that these genes were shown to be expressed in the gut of *D. melanogaster* [40, 41]. The gene for Rab-11 was induced in *A. aegypti* after 12 h of toxin ingestion which is consistent with previous reports that showed that Rab-5 and Rab-11 had an important role in the response to Cry5B in *C. elegans* [42]. Rab-5 and Rab-11 of *C. elegans* are thought to regulate a pathway through which Cry5B exposed cells may remove and recycle damaged membranes, since it was shown that absence of either of these two proteins results in a hypersensitive phenotype to Cry toxin exposure [42]. Endocytosis is also utilized in the clearance of membranes damaged by toxin pores of *S. aureus* α-toxin [15] and *S. pyogenes* streptolysin-O [43]. Thus, while the specific genes involved may vary the overall cellular responses detected in this work are similar to the responses observed in other organism to different pore forming toxins.

Small GTPase signaling networks involve MAPK proteins and, since genes related to these transduction modules are induced, it could partly explain the cellular response to Cry toxins provided by JNK and p38 [31]. It was previously reported the participation of the p38 MAPK signaling pathway in the response and defense against Cry11Aa toxin in *A. aegypti* [18, 25].

JNK and p38 have been shown to regulate a great number of genes in response to Cry5B in *C. elegans* [18]. The p38 MAPK pathway may be inducing expression of Ets domain containing transcription factors, which in turn could be the reason for an up regulation of metalloproteases [44]. Our data also suggest the involvement of signaling via PIP_3_ in the larval defense response. Arachidonic acid, the product of phospholipase A2 activity, has been reported to stimulate cell adhesion via a novel MAPK p38-RhoA pathway [45]. Both arachidonic acid and PIP_3_ are messenger metabolites derived from membrane phospholipids. These data may indicate a link between some enriched functional processes detected in our analysis, and the potential role that they may play in defending *A. aegypti* from the effects of pore forming toxins. However, more precise biochemical experiments are required to evaluate the cross-talk between these signaling pathways.

### Down-regulation response

*A. aegypti*’s defense response involves the down-regulation of several processes. Among these we identify genes related detoxification proteins such a cytochrome 450 and ABC transporters. Most of these down-regulated processes were observed at 12 h of toxin exposure. It is notable that similar expression patterns were observed in *Bti* tolerant *A. aegypti* strains [46], in the Cry11Aa resistant *A. aegypti* [47], as well as in chemical insecticide resistant strains of *A. aegypti* [48]. It was suggested that this down-regulation may be a metabolic tradeoff for the energy spent on other defense processes [46, 48]. In the absence of pathogen-associated molecular patterns (PAMPs) from bacterial components to stimulate innate immunity in our purified Cry11Aa crystals, our results suggest that the cells divert metabolism to membrane remodeling and tissue junction sealing, rather than functions for which no stimuli is detected. This energy saving measures could explain the initial down-regulation of cell proliferation, although this process seems to be induced at the last time-point analyzed, perhaps to substitute irreparably damaged cells after wound-healing. Our analysis also revealed an overall repression of transmembrane transport, especially that concerning sulfate metabolism and carbohydrates, which seems inconsistent with attempts to reestablish solute gradients after the disruption produced by Cry11Aa pores at the cell membrane. However, the cell appears to shift first to restore membrane integrity and repress any energy consuming activities not related to this process.

As mentioned above we found genes with ABC transporter domains down-regulated DEG. This is relevant since some Cry1Ac resistance phenotypes in different lepidopteran species, such as *Plutella xylostella, Bombyx mori, Heliothis virescens* and *Helicoverpa armigera* larvae are linked to mutations in the ABCC2 transporters [49–51], and negative differential expression of genes involved in detoxification activity, including ABC transporters and cytochrome 450, has been found in mosquitoes tolerant to *Bti* [46]. Many detoxification processes require energy consumption to pump offending agents out of the cell. We show here that when membrane is damage due to the pore formation of the toxin, the cell responds by resealing the cell membrane and removing damaged parts of the membrane instead of activation of transmembrane transports and detoxification. A possible explanation is that down regulation of detoxification-related genes could be an energy saving process as previously suggested for *Bti-* and Cry11Aa*-* resistant *Ae. aegypti* mosquitoes that also showed under-transcription of enzymes classically involved in the detoxification [46–48].

### Comparison with mosquito resistant to Bti toxins and with Cry toxin intoxications in different insect orders

It is interesting to mention that the convergence of some processes from the transitory response to Cry11Aa intoxication with that of a genetically established resistance to *Bti* toxins [46–48] may suggests that resistance involves the selection of mutations that could confer a metabolic state similar to a constitutive toxin defense response. Awareness of the pathways utilized by midgut cells to contend with toxin damage may allow us to better predict possible resistance mechanisms that could occur by field application of *Bt* formulations.

Importantly, there is a great deal of overlap in genome wide transcription changes and functional enrichment of *A. aegypti* reported here with that found for intoxication of *T. molitor* with Cry3Aa [24]. For example, both analyses found that toxin treated larvae show a repression of enzymes related to antioxidant processes, as well as functions associated with DNA replication and cell division. Carbohydrate and tricarboxylic acid enzymes are also repressed in both organisms. Signaling through MAPK and small GTPases are induced in both organisms upon intoxication, as are functions related to maintaining cell integrity and cytoskeleton complexes. All of these data suggest that insects from different orders respond similarly to Cry toxin action, supporting a conserved defense response to Cry toxins in these insect species.

### Implications in the Cry11Aa mechanism of action

Finally, it appears that only fully functional pore forming Cry11Aa toxins is able to elicit the full range of transcriptional changes observed in this work. Neither Cry11Aa-mutant toxins tested in RT-qPCR assays showed high positive correlation with RNAseq data, where Cry11Aa E97A, which is unable to form a stable oligomeric pre-pore structure, was most different. The higher correlation observed with Cry11Aa V142D for some genes is similar to that observed for non-toxin exposed larvae, but could also be due to non-complete loss of function. The oligomeric structure formed by this toxin may not form a pore stable enough to cause a lethal phenotype, but that is nonetheless sufficient to be sensed by the cellular machinery.

Our data indicated that binding to the cell membrane was not enough to provoke a response, involving an intracellular cascade activation of a receptor coupled protein G that activates adenylate cyclase and PKA activities as was proposed in Zhang *et al., *2006 [52]. First, our results with the mutant toxins indicated that full pore formation is required to elicit the entire response program, at least for the genes tested. We observed activation of PIP3 response and small monomeric GTPases families which are found in the cytosol and have a molecular weight of about 21 kDa, in contrast to the proposed participation of a heterotrimeric G proteins, specifically from *G*_*s*_ family that participates in activation of adenylate cyclase [52]. Finally, we did not observed changes in PKA expression.

## Conclusions

The burden imposed on global human health by mosquito borne diseases is tremendous. Biological control of mosquito populations through the use of *Bt* toxins is an important part of strategies to diminish this burden. However, it is important to consider emergence of resistant insect vector populations due to widespread use of *Bti* preparations. Although *Bti* has been used for decades worldwide seemingly without such resistance events surfacing, the risk remains. In this work we took a glimpse at the physiological response that the *A. aegypti* midgut mounts upon action of a Cry toxin, and how the epithelial cells therein change their gene expression to defend and restore tissue integrity. This knowledge contributes to the field of *Bt* toxins in several ways. First, it complements and extends earlier transcriptomic studies performed on other insects exposed to Cry that may allow us to determine differences and similarities in response between distinct insect species. Second, it improves our understanding of the overall molecular interactions and the mode of action of Cry toxins with target cells, building upon the in vitro biochemical data with data of in vivo effects. Lastly, we now possess information that may be helpful to improve our use of *Bti* toxins for the biological control of mosquitoes. Knowing what are the defense response pathways employed by mosquito larvae to survive the activity of *Bt* toxins may potentially help in the design of better strategies or formulations that could limit the defense response in mosquito An alternative could be the use of inhibitors of pathways that may weaken the mosquito’s defense mechanisms. Another alternative is the release of mosquitoes with knock-out or mutant alleles in proteins of the defense pathways. It is clear that these ideas are just speculations that require much further work before their field application to minimize the impact of vector-borne diseases on human populations.

## Methods

### Production, purification of Cry11Aa toxins and insect bioassay

Bt strains were grown for three days on HCT medium supplemented with 10 μg/ml of erythromycin as previously described [5] for the production of crystals of Cry11Aa or its mutants E97A and V142D [35, 36]. Crystals were obtained by washing the cultures with 0.3 M NaCl/0.01 M EDTA and purified by discontinuous sucrose gradients [53]. The medium lethal concentration (LC_50_) of wild-type Cry11Aa pure crystals was determined after 24 h exposure of 4th instar *A. aegypti* larvae to different doses in a response curve in triplicate as previously reported [5] and calculated by ProBit analysis (POLO-Plus, LeOra Software).

### Intoxication and tissue dissection

For each time of each biological replicate of the intoxication assay, a dose of 500 ng Cry11Aa pure crystals per ml was administered to three cups containing each ten 4^th^-instar *A. aegypti* larvae in 100 ml of water, these experiments were performed four times. After 3, 6, 9 and 12 h of incubation with Cry11Aa crystals the midgut tissue was dissected from the larvae, taking care to remove the food bolus. Control conditions of non-intoxicated larvae at time point zero and 12 h without toxin were also analyzed in triplicate. Dead larvae or larvae that were severely affected by intoxication were discarded. Each biological replicate was conducted at the same time of the day to minimize gene expression changes due to circadian rhythms. The dissected midguts were placed in RNAlater (Qiagen) for subsequent processing and stored at −70 °C per the manufacturer’s instructions.

### RNA extraction and sequencing

Strength of differential expression analysis increases with the number of replicates used to estimate the expression levels, for this reason four biological replicates were sequenced on an Illumina platform. RNAlater buffer was removed from dissected midguts and the tissues homogenized with 27G syringes in TRIzol reagent (Ambion, Life Technologies). Cellular debris was removed by centrifugation at 12,000 *xg* for 10 min at 4 °C, and the supernatant transferred to new tubes. RNA was extracted following the manufacturer’s instructions. Total RNA was dissolved in DEPC treated water and quantified by Nanodrop 1000 (Thermo Scientific). RNA was treated with DNAse I (Thermo Scientific), and samples were cleaned with RNeasy columns (QIAGEN) following the kit’s protocol. The integrity and concentration of eluted RNA were determined by Bioanalyzer 2100 (Agilent Technologies). For sequencing, mRNA libraries were prepared from 2 μg of total RNA using standard Illumina protocols using the TruSeq RNA Sample Preparation Kit. The mRNA libraries were sequenced in 72 bp pair-ended format on a Genome Analyzer IIx (Illumina) at the Unidad Universitaria de Secuenciación Masiva y Bioinformática (UUSMB) of the Instituto de Biotecnología-UNAM facilities, or in 100 bp pair-ended format on a Illumina Hi-Seq 2000 at Laboratorio Nacional de Genómica para la Biodiversidad (LANGEBIO) of Centro de investigación y de Estudios Avanzados del Instituto Politécnico Nacional (CINVESTAV-IPN) facilities. Quality control of reads was performed at UUSMB facilities. Read quality control include data for GC content, for overrepresented reads (for example, contaminating ribosomal RNA reads). Quality per base and more parameters were obtained using the FastQC utility. A specific read is flagged as overrepresented if that identical sequence appears above the 0.1 % of total reads obtained in the sequencing of an individual sample. Longer reads were trimmed with FastX-Trimmer to 72 pb to remove adapter and lower quality bases at the ends.

### Differential expression analysis

RNAseq reads were aligned to *A. aegypti* transcripts v3.2 (Vectorbase) [54] using TopHat 2 v 2.0.12 [55] in transcriptome mode and base features file v 3.2, allowing for 2 mismatches. For each sample, a count table for all genes was generated with HTSeq Count v0.5.4p5 [56]. Aligned reads where filtered to remove reads with multiple alignments or ambiguous assignment by executing the script in “intersection non-empty” mode. Only reads with unique alignments were further analyzed. A count table for all samples of four biological replicates was compiled and used for differential expression analysis by the BioconductoR packages DEseq2 v 1.4.5 [29] and EdgeR v 3.6.8 [30], both in generalized linear model mode that included both time of toxin exposure and biological replicate of the experiment as possible sources of variation to control for differences among individuals dissected on different days. Results for DEG were obtained from comparisons of each intoxication time to non-intoxicated control. A gene was considered significant if the adjusted *p*-value was < 0.05 for DESeq2 or if False Discovery Rate was < 0.05 for EdgeR. For each intoxication time DEG reported by both tools were recovered by a custom R script.

### Functional analysis

GO enrichment analysis of biological process annotations was performed with the BioconductoR package TopGO v 2.16.0 [57] using the “Weight” algorithm. GO annotations for all genes were retrieved from Vectorbase, formatted, and used to detect enrichment of GO terms in up or down regulated DEG from each intoxication condition on genes common to both differential expression analysis tools. We used the “Weight” algorithm because it yields more informative GO terms due to the way it processes the GO ontology hierarchy, and allowed us to obtain information of more particular metabolic processes. A *p*-value of 0.1 was used as cutoff for enriched terms. GO annotations terms with less than five annotated genes were excluded from analysis to reduce statistical artifacts. Genes without a GO biological process term were further analyzed, and their predicted Interpro functional domains were retrieved from Vectorbase. Frequency for domains was counted with a custom Perl script and those above the 90^th^ percentile were kept. Finally, we conducted pBLAST queries [34] against NR database with genes for which no Interpro domain could be retrieved. Only matches having more that 40 % of query identity and an *e*-value of < e-8 were considered significant.

### Clustering analysis

Through DEseq2 a variance stabilized transformation was performed on all log_2_ fold changes for all samples. These data were filtered to retain only those genes that were determined to have significant differential expression in at least one treatment and were part of the genes common to DESeq2 and EdgeR analysis. Data for each gene were summarized as mean of replicates per treatment. Clustering of individual DEGs that followed the same expression pattern over the 4 time points was performed with Cluster 3.0 (Stanford University), centering values by mean and using complete linkage and euclidian distances as parameters. Java Treeview v1.1.6r4 [58] was used to visualize results and to recover clusters of genes with common expression profiles throughout the intoxication experiment. GO enrichment analysis was performed within these clusters as before.

### RT-qPCR target selection and validation

Targets for validation by RT-qPCR were selected from DEG. These targets were selected according to three criteria: high statistical significance of differential expression, absolute log_2_ fold change larger than 2, and statistical significance in more than one tested condition. Some target genes do not cover all criteria but were nonetheless selected due to their very high significance or log_2_ fold change. Primers were designed to amplify products of 100–180 bp. All primer pairs were designed in the same exon with exception of two pairs of primers for AAEL003633 and AAEL008192 genes, which were located in two exons separated by an intron. For cDNA template synthesis from RNA samples we used Superscript III Reverse Transcriptase (Invitrogen) following the manufacturer’s instructions. For all primers, a control amplification was performed with the total RNA in absence of reverse transcriptase, in these controls the signal was absent or extremely low indicating that contamination of our total RNA samples was negligible.

Dynamic range for signal detection was determined by SYBR-green (Thermo Scientific) RT-qPCR amplification of some selected genes on cDNA prepared using 5000, 500, 50, 5 and 0.5 μg of total RNA from *A. aegypti* midgut on Illumina Eco equipment. To determine primer amplification efficiency, 1.5 μg of total RNA from each time of a LC_50_ intoxication replicate were mixed for cDNA synthesis. This template was used for SYBR green RT-qPCR amplification in 10-fold serial dilutions for each primer pair.

RT-qPCR was performed (Roche Light Cycler 480) using a Cry11Aa LC_50_ intoxication biological replicate that was not sequenced. The RT-qPCR data for all 14 selected genes presented in Additional file 5: Figure S1 were obtained for 0, 3, 6, 9 and 12 h after administration of Cry11Aa. In the case of Cry11Aa Cry11Aa E97A, and Cry11Aa V142D mutants the RT-qPCR data were obtained after 0, 9, and 12 h since these times were the ones that showed most important significant changes in expression. The controls of no-toxin exposed larvae were also done at 0, 9 and 12 h. Primer efficiency correction was used in ΔΔCq relative quantitation analysis. The ribosomal protein S3 gene was used for reference and normalization. Transcript abundance ratios between conditions were log_2_ transformed and the Spearman correlation determined with corresponding RNAseq log_2_ fold data for each gene.

### Western blot

Midguts were dissected from 4^th^ instar *A. aegypti* larvae and pools of 50 entire midguts in 100 μl of rehydration buffer (7 M urea, 2 M thiourea, 4 % CHAPS, 40 mM dithiothreitol, 0.5 % pharmalyte or IPG buffer [GE Life Sciences], 0.002 % bromophenol blue, 2.5 ml) containing protease inhibitors (Complete, Roche Diagnostics) and were kept at −80 °C until processed. Midgut pools were homogenized with a motorized pellet pestle (Motor Sigma-Aldrich Z359971.1EA) on ice. Ten μg of midgut protein was fractionated by 10 % SDS-PAGE and transferred to nitrocellulose membrane. Western blot was performed as described by Aranda *et al.,* (1996) [59]. JNK protein was detected after incubation of 1 h with polyclonal anti-JNK (1/5,000; JNK2 N-18: sc-827 Santacruz Biotech. Inc). Visualization was performed with goat anti-rabbit secondary antibody coupled to horseradish peroxidase (Amersham) (1/10,000; 1 h), followed by Super Signal chemiluminescence Luminol substrate (Pierce), according to the manufacturer’s instructions.

### Data accessibility

Raw RNA-seq data and gene expression abundance measurements for this study have been deposited at Gene Expression Omnibus under accession code GSE74785. Read data can accessed directly in Sequence Read Archive with accession code SRP065731.

## Additional files

Additional file 1: Table S1. TopHat2 alignment rates. Number of total reads is rounded to closest million. (DOCX 73 kb) Additional file 2: Table S2. Complete list of 1060 differentially expressed genes across all experimental conditions (3-12 h of Cry11Aa toxin administration) and gene description (if available), by expression profile cluster. (DOCX 325 kb) Additional file 3: Table S3. List of differentially expressed genes in control (non-toxin exposed larvae) RNAseq data at 12 h. (DOCX 68 kb) Additional file 4: Table S4. List of all Interpro domains found in differential expressed genes without biological process GO annotation. (DOCX 92 kb) Additional file 5: Figure S1. RNAseq and log2 transformed RT-qPCR fold changes. Data is presented for RNAseq and RT-qPCR at 3, 6, 9, and 12 h of an LC_50_ Cry11Aa treatment. Also shown are RT-qPCR values for 9 and 12 h of unexposed larvae or Cry11Aa non-toxic mutants treatments. All log_2_ fold changes are referred to control larvae at the start of respective treatment. Panel A and B show genes with high correlation between RNAseq data and RT-qPCR of Cry11Aa. Panel C shows the three genes with low or no positive correlation between these data. (ZIP 779 kb) Additional file 6: Figure S2. Characterization of JNK expression by Western Blot analysis using α-JNK antibody. Presence of JNK was determined on protein extracts of midguts of *A. aegypti* larvae exposed to Cry11Aa for 9 or 12 h (lanes 4 and 5, respectively). Proteins were separated by SDS-PAGE and transferred to PVDF membranes for Western Blotting with human αJNK2 antibody. Non-toxin exposed larvae dissected at corresponding times were used as controls (lanes 1 to 3). (TIF 1042 kb)

## Abbreviations

ALP: alkaline phosphatases
APN: aminopeptidases
*Bt*: *Bacillus thuringiensis*
*Bti*: *Bacillus thuringiensis* subsp. *israelensis*
DEG: differentially expressed genes
WHO: World Health Organization
