# Supplementary material for: Transcriptional cellular responses in midgut tissue of Aedes aegypti larvae following intoxication with Cry11Aa toxin from Bacillus thuringiensis
Source: BMC Genomics. 2015 Dec 9;16:1042. doi: 10.1186/s12864-015-2240-7 (PMC4673840; doi:10.1186/s12864-015-2240-7)
Supplement: Additional file 1: Table S1. — TopHat2 alignment rates. Number of total reads is rounded to closest million. (DOCX 73 kb) [file 12864_2015_2240_MOESM1_ESM.docx]

**Table S1**. TopHat2 alignment rates. Number of total reads is rounded to closest million

| **Replicate** | **Condition** | **Total Reads** | **% genome alignment** | **% transcriptome alignment** | **% unique alignment (Transcriptome)** |
| --- | --- | --- | --- | --- | --- |
| 1 | **0 h ( no tox)** | 38 M | 76.63 | 71.31 | 70.30 |
|  | **3 h** | 60 M | 77.54 | 67.99 | 67.85 |
|  | **6 h** | 30 M | 76.07 | 69.11 | 67.80 |
|  | **9 h** | 60 M | 77.81 | 71.40 | 71.40 |
|  | **12 h** | 50 M | 77.98 | 71.59 | 71.23 |
| 2 | **0 h (no tox)** | 40 M | 77.04 | 70.57 | 69.49 |
|  | **3 h** | 50 M | 78.57 | 68.72 | 67.66 |
|  | **6 h** | 54 M | 79.72 | 66.49 | 65.31 |
|  | **9 h** | 32 M | 78.94 | 70.98 | 64.54 |
|  | **12 h** | 33 M | 78.98 | 70.36 | 65.76 |
| 3 | **0 h (no tox)** | 23 M | 77.80 | 71.30 | 69.50 |
|  | **3 h** | 23 M | 77.34 | 70.09 | 68.19 |
|  | **6 h** | 22 M | 77.78 | 71.04 | 69.46 |
|  | **9 h** | 21 M | 77.68 | 71.16 | 69.40 |
|  | **12 h** | 22 M | 77.03 | 71.67 | 69.43 |
| 4 | **0 h (no tox)** | 57 M | NA | 69.47 | 68.83 |
|  | **3 h** | 46 M | NA | 72.71 | 71.85 |
|  | **6 h** | 57 M | NA | 71.34 | 70.21 |
|  | **9 h** | 59 M | NA | 70.83 | 69.75 |
|  | **12 h** | 53 M | NA | 70.86 | 69.26 |
