# Supplementary material for: Transcriptional cellular responses in midgut tissue of Aedes aegypti larvae following intoxication with Cry11Aa toxin from Bacillus thuringiensis
Source: BMC Genomics. 2015 Dec 9;16:1042. doi: 10.1186/s12864-015-2240-7 (PMC4673840; doi:10.1186/s12864-015-2240-7)
Supplement: Additional file 2: Table S2. — Complete list of 1060 differentially expressed genes across all experimental conditions (3-12 h of Cry11Aa toxin administration) and gene description (if available), by expression profile cluster. (DOCX 325 kb) [file 12864_2015_2240_MOESM2_ESM.docx]

**Table S2**. Complete list of 1060 differentially expressed genes across all experimental conditions (3-12 h of Cry11Aa toxin administration) and gene description (if available), by expression profile cluster.

| Gene stable ID | Gene description | DEseq2 log_2_ Fold Change | | | |
| --- | --- | --- | --- | --- | --- |
|  |  | 3 h | 6 h | 9 h | 12 h |
| Down Regulated  (cluster A) |  |  |  |  |  |
| [AAEL000101](https://www.vectorbase.org/aedes_aegypti/Gene/Summary?db=core;g=AAEL000101) | AMP dependent coa ligase [Source:VB External Description;Acc:AAEL000101] | 0.095766 | 0.200956 | 0.130571 | -0.95506 |
| [AAEL000119](https://www.vectorbase.org/aedes_aegypti/Gene/Summary?db=core;g=AAEL000119) | AMP dependent coa ligase [Source:VB External Description;Acc:AAEL000119] | 0.096951 | 0.152915 | 0.140512 | -1.05869 |
| [AAEL000143](https://www.vectorbase.org/aedes_aegypti/Gene/Summary?db=core;g=AAEL000143) |  | -0.18717 | -0.21283 | -0.34539 | -0.61059 |
| [AAEL000164](https://www.vectorbase.org/aedes_aegypti/Gene/Summary?db=core;g=AAEL000164) | d-amino acid oxidase [Source:VB External Description;Acc:AAEL000164] | -0.16807 | -0.02663 | 0.173502 | -0.79534 |
| [AAEL000172](https://www.vectorbase.org/aedes_aegypti/Gene/Summary?db=core;g=AAEL000172) |  | -0.19573 | -0.64222 | -0.686 | -0.7962 |
| [AAEL000271](https://www.vectorbase.org/aedes_aegypti/Gene/Summary?db=core;g=AAEL000271) | gamma-glutamyl hydrolase [Source:VB External Description;Acc:AAEL000271] | -0.44834 | -0.57733 | -0.05511 | -0.88299 |
| [AAEL000314](https://www.vectorbase.org/aedes_aegypti/Gene/Summary?db=core;g=AAEL000314) | malate dehydrogenase [Source:VB External Description;Acc:AAEL000314] | -0.15999 | -0.44688 | -0.33889 | -0.74982 |
| [AAEL000415](https://www.vectorbase.org/aedes_aegypti/Gene/Summary?db=core;g=AAEL000415) | AMP dependent coa ligase [Source:VB External Description;Acc:AAEL000415] | 0.302869 | -0.13447 | -0.4729 | -0.62204 |
| [AAEL000417](https://www.vectorbase.org/aedes_aegypti/Gene/Summary?db=core;g=AAEL000417) | monocarboxylate transporter [Source:VB External Description;Acc:AAEL000417] | -0.0098 | -0.19955 | -0.33217 | -0.80236 |
| [AAEL000427](https://www.vectorbase.org/aedes_aegypti/Gene/Summary?db=core;g=AAEL000427) |  | -0.07907 | 0.081998 | -0.26276 | -0.93238 |
| [AAEL000429](https://www.vectorbase.org/aedes_aegypti/Gene/Summary?db=core;g=AAEL000429) |  | -0.15738 | -0.45034 | -0.60377 | -1.01553 |
| [AAEL000440](https://www.vectorbase.org/aedes_aegypti/Gene/Summary?db=core;g=AAEL000440) | DNA repair/transcription protein met18/mms19 [Source:VB External Description;Acc:AAEL000440] | -0.09972 | 0.047952 | -0.69113 | -0.79492 |
| [AAEL000452](https://www.vectorbase.org/aedes_aegypti/Gene/Summary?db=core;g=AAEL000452) | histone acetyltransferase gcn5 [Source:VB External Description;Acc:AAEL000452] | 0.003378 | -0.27654 | -0.25294 | -0.63714 |
| [AAEL000476](https://www.vectorbase.org/aedes_aegypti/Gene/Summary?db=core;g=AAEL000476) |  | -0.24749 | -0.5114 | -0.64852 | -0.96462 |
| [AAEL000522](https://www.vectorbase.org/aedes_aegypti/Gene/Summary?db=core;g=AAEL000522) |  | 0.02937 | -0.35017 | -0.08479 | -1.02369 |
| [AAEL000581](https://www.vectorbase.org/aedes_aegypti/Gene/Summary?db=core;g=AAEL000581) |  | 0.077026 | -0.27091 | -0.49832 | -1.28606 |
| [AAEL000590](https://www.vectorbase.org/aedes_aegypti/Gene/Summary?db=core;g=AAEL000590) | Defense repressor 1 [Source:VB Community Annotation;Acc:AAEL000590] | -0.18865 | -0.24931 | -0.23237 | -0.75878 |
| [AAEL000602](https://www.vectorbase.org/aedes_aegypti/Gene/Summary?db=core;g=AAEL000602) | GPCR Orphan/Putative Class B Family [Source:VB Community Annotation;Acc:AAEL000602] | -0.08174 | -0.40509 | -0.3938 | -0.73724 |
| [AAEL000622](https://www.vectorbase.org/aedes_aegypti/Gene/Summary?db=core;g=AAEL000622) |  | -0.00532 | -0.16958 | -0.22291 | -0.5509 |
| [AAEL000645](https://www.vectorbase.org/aedes_aegypti/Gene/Summary?db=core;g=AAEL000645) |  | -0.07535 | -0.35595 | -0.37428 | -0.62016 |
| [AAEL000658](https://www.vectorbase.org/aedes_aegypti/Gene/Summary?db=core;g=AAEL000658) |  | -0.07927 | -0.05223 | -1.04528 | -1.48559 |
| [AAEL000670](https://www.vectorbase.org/aedes_aegypti/Gene/Summary?db=core;g=AAEL000670) | methionine sulfoxide reductase [Source:VB External Description;Acc:AAEL000670] | -0.35448 | -1.05834 | -0.39262 | -1.78848 |
| [AAEL000746](https://www.vectorbase.org/aedes_aegypti/Gene/Summary?db=core;g=AAEL000746) | NADP-specific isocitrate dehydrogenase [Source:VB External Description;Acc:AAEL000746] | -0.18237 | -0.27135 | -0.23847 | -0.65461 |
| [AAEL000824](https://www.vectorbase.org/aedes_aegypti/Gene/Summary?db=core;g=AAEL000824) |  | -0.32676 | -0.30108 | -0.59971 | -0.84946 |
| [AAEL000851](https://www.vectorbase.org/aedes_aegypti/Gene/Summary?db=core;g=AAEL000851) | predicted G-protein coupled receptor (GPCR) [Source:VB External Description;Acc:AAEL000851] | -0.18262 | -0.53075 | -0.40106 | -0.86686 |
| [AAEL000902](https://www.vectorbase.org/aedes_aegypti/Gene/Summary?db=core;g=AAEL000902) | sugar transporter [Source:VB External Description;Acc:AAEL000902] | -0.02576 | -0.59108 | -0.39625 | -1.01638 |
| [AAEL000920](https://www.vectorbase.org/aedes_aegypti/Gene/Summary?db=core;g=AAEL000920) |  | -0.19902 | -0.42589 | -0.46228 | -1.20762 |
| [AAEL000952](https://www.vectorbase.org/aedes_aegypti/Gene/Summary?db=core;g=AAEL000952) |  | 0.047727 | -0.38289 | -0.48005 | -0.61911 |
| [AAEL000956](https://www.vectorbase.org/aedes_aegypti/Gene/Summary?db=core;g=AAEL000956) |  | -0.25576 | -0.51921 | -0.7382 | -1.50986 |
| [AAEL001020](https://www.vectorbase.org/aedes_aegypti/Gene/Summary?db=core;g=AAEL001020) | anterior fat body protein [Source:VB External Description;Acc:AAEL001020] | -0.35396 | -0.40106 | -0.63568 | -0.68185 |
| [AAEL001022](https://www.vectorbase.org/aedes_aegypti/Gene/Summary?db=core;g=AAEL001022) | anterior fat body protein [Source:VB External Description;Acc:AAEL001022] | -0.61636 | -0.5166 | -0.76845 | -1.00107 |
| [AAEL001069](https://www.vectorbase.org/aedes_aegypti/Gene/Summary?db=core;g=AAEL001069) | histone deacetylase [Source:VB External Description;Acc:AAEL001069] | -0.1956 | -0.25139 | -0.08352 | -1.04245 |
| [AAEL001128](https://www.vectorbase.org/aedes_aegypti/Gene/Summary?db=core;g=AAEL001128) | AMP dependent coa ligase [Source:VB External Description;Acc:AAEL001128] | 0.085362 | -0.04998 | -0.03859 | -0.55098 |
| [AAEL001143](https://www.vectorbase.org/aedes_aegypti/Gene/Summary?db=core;g=AAEL001143) |  | 0.181329 | -0.48013 | -0.40266 | -0.87987 |
| [AAEL001198](https://www.vectorbase.org/aedes_aegypti/Gene/Summary?db=core;g=AAEL001198) | sodium/solute symporter [Source:VB External Description;Acc:AAEL001198] | 0.122382 | -0.11279 | -0.38559 | -0.60937 |
| [AAEL001209](https://www.vectorbase.org/aedes_aegypti/Gene/Summary?db=core;g=AAEL001209) | sodium-dependent phosphate transporter [Source:VB External Description;Acc:AAEL001209] | -0.33593 | -0.1674 | -0.7708 | -0.78188 |
| [AAEL001257](https://www.vectorbase.org/aedes_aegypti/Gene/Summary?db=core;g=AAEL001257) | sugar transporter [Source:VB External Description;Acc:AAEL001257] | 0.203934 | -0.54081 | -1.19777 | -0.72702 |
| [AAEL001419](https://www.vectorbase.org/aedes_aegypti/Gene/Summary?db=core;g=AAEL001419) | nocturnin [Source:VB External Description;Acc:AAEL001419] | -0.09186 | -0.43845 | -0.28565 | -0.83241 |
| [AAEL001422](https://www.vectorbase.org/aedes_aegypti/Gene/Summary?db=core;g=AAEL001422) | proteasome inhibitor [Source:VB External Description;Acc:AAEL001422] | -0.0803 | -0.4161 | -0.29379 | -0.53489 |
| [AAEL001607](https://www.vectorbase.org/aedes_aegypti/Gene/Summary?db=core;g=AAEL001607) | galactose-1-phosphate uridylyltransferase [Source:VB External Description;Acc:AAEL001607] | -0.2006 | -0.30001 | -0.60128 | -0.72987 |
| [AAEL001642](https://www.vectorbase.org/aedes_aegypti/Gene/Summary?db=core;g=AAEL001642) | 6-pyruvoyltetrahydropterin synthase, putative [Source:VB External Description;Acc:AAEL001642] | -0.16771 | -0.6683 | -0.8394 | -1.02687 |
| [AAEL001647](https://www.vectorbase.org/aedes_aegypti/Gene/Summary?db=core;g=AAEL001647) |  | 0.087707 | -0.27726 | -0.06676 | -0.77469 |
| [AAEL001730](https://www.vectorbase.org/aedes_aegypti/Gene/Summary?db=core;g=AAEL001730) |  | -0.96599 | -1.52388 | -1.48573 | -1.02597 |
| [AAEL001807](https://www.vectorbase.org/aedes_aegypti/Gene/Summary?db=core;g=AAEL001807) | cytochrome P450 (CYP9M9) [Source:VB Community Annotation;Acc:AAEL001807] | -0.18155 | 0.131562 | -0.38837 | -0.62733 |
| [AAEL001816](https://www.vectorbase.org/aedes_aegypti/Gene/Summary?db=core;g=AAEL001816) | glucosyl/glucuronosyl transferases [Source:VB External Description;Acc:AAEL001816] | -0.31814 | -0.34931 | -1.23612 | -0.93277 |
| [AAEL001902](https://www.vectorbase.org/aedes_aegypti/Gene/Summary?db=core;g=AAEL001902) | glutamate decarboxylase [Source:VB External Description;Acc:AAEL001902] | -0.12777 | -1.42841 | -1.01756 | -0.2575 |
| [AAEL002019](https://www.vectorbase.org/aedes_aegypti/Gene/Summary?db=core;g=AAEL002019) |  | -0.14234 | -0.30203 | -0.31148 | -0.65106 |
| [AAEL002043](https://www.vectorbase.org/aedes_aegypti/Gene/Summary?db=core;g=AAEL002043) | cytochrome P450 (CYP305A5) [Source:VB Community Annotation;Acc:AAEL002043] | -0.12031 | -0.39365 | -0.13712 | -0.89507 |
| [AAEL002063](https://www.vectorbase.org/aedes_aegypti/Gene/Summary?db=core;g=AAEL002063) | cationic amino acid transporter [Source:VB External Description;Acc:AAEL002063] | 0.015307 | -0.0915 | -0.39774 | -0.6542 |
| [AAEL002085](https://www.vectorbase.org/aedes_aegypti/Gene/Summary?db=core;g=AAEL002085) | cytochrome P450 (CYP4H31) [Source:VB Community Annotation;Acc:AAEL002085] | -0.06514 | 0.487049 | -0.56293 | -0.99281 |
| [AAEL002113](https://www.vectorbase.org/aedes_aegypti/Gene/Summary?db=core;g=AAEL002113) | 3-oxoacyl-[acyl-carrier-protein] synthase [Source:VB External Description;Acc:AAEL002113] | -0.10197 | -0.58316 | -0.71881 | -0.89928 |
| [AAEL002141](https://www.vectorbase.org/aedes_aegypti/Gene/Summary?db=core;g=AAEL002141) | endothelin-converting enzyme [Source:VB External Description;Acc:AAEL002141] | -0.5232 | -0.1546 | 0.672594 | -1.06754 |
| [AAEL002145](https://www.vectorbase.org/aedes_aegypti/Gene/Summary?db=core;g=AAEL002145) | gonadotropin inducible transcription factor [Source:VB External Description;Acc:AAEL002145] | -0.1589 | -0.33196 | -0.27842 | -0.62718 |
| [AAEL002278](https://www.vectorbase.org/aedes_aegypti/Gene/Summary?db=core;g=AAEL002278) | guanylate cyclase, putative [Source:VB External Description;Acc:AAEL002278] | 0.078781 | -0.47825 | -0.63505 | -0.96797 |
| [AAEL002335](https://www.vectorbase.org/aedes_aegypti/Gene/Summary?db=core;g=AAEL002335) | atbf1 [Source:VB External Description;Acc:AAEL002335] | -0.01483 | -0.40012 | -0.14137 | -0.76196 |
| [AAEL002370](https://www.vectorbase.org/aedes_aegypti/Gene/Summary?db=core;g=AAEL002370) |  | -0.03191 | -0.34389 | -0.30043 | -1.08551 |
| [AAEL002381](https://www.vectorbase.org/aedes_aegypti/Gene/Summary?db=core;g=AAEL002381) |  | -0.04873 | -0.01733 | -1.15498 | -1.11985 |
| [AAEL002384](https://www.vectorbase.org/aedes_aegypti/Gene/Summary?db=core;g=AAEL002384) |  | -0.09068 | -0.34918 | -0.5082 | -0.68973 |
| [AAEL002457](https://www.vectorbase.org/aedes_aegypti/Gene/Summary?db=core;g=AAEL002457) |  | 0.269441 | -0.4323 | -0.2903 | -0.85151 |
| [AAEL002475](https://www.vectorbase.org/aedes_aegypti/Gene/Summary?db=core;g=AAEL002475) |  | 0.033912 | -0.125 | -0.17305 | -0.58765 |
| [AAEL002487](https://www.vectorbase.org/aedes_aegypti/Gene/Summary?db=core;g=AAEL002487) | P53 regulated pa26 nuclear protein sestrin [Source:VB External Description;Acc:AAEL002487] | -0.17536 | -0.29387 | -0.96441 | -1.18922 |
| [AAEL002497](https://www.vectorbase.org/aedes_aegypti/Gene/Summary?db=core;g=AAEL002497) |  | -0.4933 | -1.15705 | -1.15552 | -1.83425 |
| [AAEL002581](https://www.vectorbase.org/aedes_aegypti/Gene/Summary?db=core;g=AAEL002581) | structural maintenance of chromosomes 6 smc6 [Source:VB External Description;Acc:AAEL002581] | -0.04623 | -0.66452 | -0.93447 | -0.67552 |
| [AAEL002633](https://www.vectorbase.org/aedes_aegypti/Gene/Summary?db=core;g=AAEL002633) | cytochrome P450 [Source:VB Community Annotation;Acc:AAEL002633] | -0.05222 | -0.09635 | 0.428075 | -0.61921 |
| [AAEL002638](https://www.vectorbase.org/aedes_aegypti/Gene/Summary?db=core;g=AAEL002638) | cytochrome P450 (CYP9J6) [Source:VB Community Annotation;Acc:AAEL002638] | 0.043386 | 0.236912 | 0.223668 | -0.74838 |
| [AAEL002668](https://www.vectorbase.org/aedes_aegypti/Gene/Summary?db=core;g=AAEL002668) | AMP dependent ligase [Source:VB External Description;Acc:AAEL002668] | -0.12081 | -0.45135 | -0.42794 | -1.12828 |
| [AAEL002796](https://www.vectorbase.org/aedes_aegypti/Gene/Summary?db=core;g=AAEL002796) | l-asparaginase i [Source:VB External Description;Acc:AAEL002796] | -0.12397 | -0.38357 | -0.37887 | -0.8742 |
| [AAEL002857](https://www.vectorbase.org/aedes_aegypti/Gene/Summary?db=core;g=AAEL002857) |  | -0.11211 | 0.00403 | -1.05518 | -0.89403 |
| [AAEL002877](https://www.vectorbase.org/aedes_aegypti/Gene/Summary?db=core;g=AAEL002877) | kinesin-like protein KIF17 [Source:VB External Description;Acc:AAEL002877] | -0.14887 | -0.43054 | -1.21658 | -1.19903 |
| [AAEL003086](https://www.vectorbase.org/aedes_aegypti/Gene/Summary?db=core;g=AAEL003086) |  | -1.90652 | -1.44197 | -2.60096 | -2.68741 |
| [AAEL003090](https://www.vectorbase.org/aedes_aegypti/Gene/Summary?db=core;g=AAEL003090) | malate synthase [Source:VB External Description;Acc:AAEL003090] | 0.083021 | -0.172 | -0.13637 | -1.02817 |
| [AAEL003098](https://www.vectorbase.org/aedes_aegypti/Gene/Summary?db=core;g=AAEL003098) | glucosyl/glucuronosyl transferases [Source:VB External Description;Acc:AAEL003098] | -0.25344 | -0.05324 | -0.24902 | -0.57258 |
| [AAEL003102](https://www.vectorbase.org/aedes_aegypti/Gene/Summary?db=core;g=AAEL003102) | glucosyl/glucuronosyl transferases [Source:VB External Description;Acc:AAEL003102] | 0.145797 | -0.13101 | -0.61379 | -0.71241 |
| [AAEL003187](https://www.vectorbase.org/aedes_aegypti/Gene/Summary?db=core;g=AAEL003187) | Carboxy/choline esterase Alpha Esterase [Source:VB Community Annotation;Acc:AAEL003187] | 0.252859 | 0.244972 | 0.383068 | -0.67487 |
| [AAEL003277](https://www.vectorbase.org/aedes_aegypti/Gene/Summary?db=core;g=AAEL003277) |  | -0.46733 | -0.69378 | -0.53673 | -0.91695 |
| [AAEL003283](https://www.vectorbase.org/aedes_aegypti/Gene/Summary?db=core;g=AAEL003283) | bitesize isoform [Source:VB External Description;Acc:AAEL003283] | -0.22522 | -0.50601 | -0.44304 | -0.84561 |
| [AAEL003339](https://www.vectorbase.org/aedes_aegypti/Gene/Summary?db=core;g=AAEL003339) |  | -0.47275 | -0.69748 | -0.67427 | -2.01703 |
| [AAEL003347](https://www.vectorbase.org/aedes_aegypti/Gene/Summary?db=core;g=AAEL003347) | CRAL/TRIO domain-containing protein [Source:VB External Description;Acc:AAEL003347] | 0.111873 | -0.38095 | -0.65094 | -1.04902 |
| [AAEL003355](https://www.vectorbase.org/aedes_aegypti/Gene/Summary?db=core;g=AAEL003355) |  | -0.59969 | -0.97849 | -1.16368 | -0.89964 |
| [AAEL003424](https://www.vectorbase.org/aedes_aegypti/Gene/Summary?db=core;g=AAEL003424) | zinc carboxypeptidase [Source:VB External Description;Acc:AAEL003424] | -0.48048 | -0.62598 | -1.28581 | -1.24421 |
| [AAEL003428](https://www.vectorbase.org/aedes_aegypti/Gene/Summary?db=core;g=AAEL003428) |  | -0.11788 | -0.24233 | 0.038827 | -0.85728 |
| [AAEL003430](https://www.vectorbase.org/aedes_aegypti/Gene/Summary?db=core;g=AAEL003430) |  | -0.18431 | -0.20847 | -0.32782 | -0.57068 |
| [AAEL003438](https://www.vectorbase.org/aedes_aegypti/Gene/Summary?db=core;g=AAEL003438) |  | 0.038793 | 0.018553 | -0.16601 | -0.75922 |
| [AAEL003495](https://www.vectorbase.org/aedes_aegypti/Gene/Summary?db=core;g=AAEL003495) |  | 0.029016 | -0.33168 | -0.24847 | -0.70533 |
| [AAEL003548](https://www.vectorbase.org/aedes_aegypti/Gene/Summary?db=core;g=AAEL003548) | sulphate transporter [Source:VB External Description;Acc:AAEL003548] | -0.15987 | -0.26397 | -0.02261 | -0.85861 |
| [AAEL003587](https://www.vectorbase.org/aedes_aegypti/Gene/Summary?db=core;g=AAEL003587) | rhoGTPase [Source:VB External Description;Acc:AAEL003587] | -0.13181 | -0.44642 | -0.41216 | -1.02473 |
| [AAEL003588](https://www.vectorbase.org/aedes_aegypti/Gene/Summary?db=core;g=AAEL003588) | DNA-J/hsp40 [Source:VB External Description;Acc:AAEL003588] | -0.25749 | -0.47457 | -0.96264 | -1.22198 |
| [AAEL003591](https://www.vectorbase.org/aedes_aegypti/Gene/Summary?db=core;g=AAEL003591) | xaa-pro dipeptidase app(e.coli) [Source:VB External Description;Acc:AAEL003591] | -0.24326 | -0.61342 | -0.45351 | -1.18129 |
| [AAEL003594](https://www.vectorbase.org/aedes_aegypti/Gene/Summary?db=core;g=AAEL003594) | kinectin, putative [Source:VB External Description;Acc:AAEL003594] | -0.08206 | -0.43617 | -0.46038 | -0.84948 |
| [AAEL003599](https://www.vectorbase.org/aedes_aegypti/Gene/Summary?db=core;g=AAEL003599) |  | -0.26831 | -0.1123 | -0.26271 | -1.18933 |
| [AAEL003623](https://www.vectorbase.org/aedes_aegypti/Gene/Summary?db=core;g=AAEL003623) |  | 0.117706 | -0.082 | -0.2525 | -0.7393 |
| [AAEL003633](https://www.vectorbase.org/aedes_aegypti/Gene/Summary?db=core;g=AAEL003633) | sucrose transport protein [Source:VB External Description;Acc:AAEL003633] | -0.08093 | -0.38363 | -0.68455 | -1.38165 |
| [AAEL003703](https://www.vectorbase.org/aedes_aegypti/Gene/Summary?db=core;g=AAEL003703) | scarlet protein [Source:VB External Description;Acc:AAEL003703] | -0.38711 | -0.90672 | -0.74327 | -1.10014 |
| [AAEL003792](https://www.vectorbase.org/aedes_aegypti/Gene/Summary?db=core;g=AAEL003792) |  | -0.42209 | -0.37186 | -0.19894 | -0.62273 |
| [AAEL003807](https://www.vectorbase.org/aedes_aegypti/Gene/Summary?db=core;g=AAEL003807) |  | -0.21765 | -0.1428 | -0.16249 | -0.66208 |
| [AAEL003810](https://www.vectorbase.org/aedes_aegypti/Gene/Summary?db=core;g=AAEL003810) | sugar transporter [Source:VB External Description;Acc:AAEL003810] | -0.48613 | -0.30954 | -0.40753 | -0.83565 |
| [AAEL003842](https://www.vectorbase.org/aedes_aegypti/Gene/Summary?db=core;g=AAEL003842) |  | -0.14157 | -0.11465 | -0.13146 | -0.9395 |
| [AAEL003905](https://www.vectorbase.org/aedes_aegypti/Gene/Summary?db=core;g=AAEL003905) | alkaline phosphatase [Source:VB External Description;Acc:AAEL003905] | -1.69516 | -2.8763 | -2.06855 | -1.50208 |
| [AAEL003909](https://www.vectorbase.org/aedes_aegypti/Gene/Summary?db=core;g=AAEL003909) |  | -0.075 | 0.105993 | -0.07863 | -0.71617 |
| [AAEL003963](https://www.vectorbase.org/aedes_aegypti/Gene/Summary?db=core;g=AAEL003963) | calpain 4, 6, 7, invertebrate [Source:VB External Description;Acc:AAEL003963] | 0.061776 | -0.27518 | -0.40336 | -1.36308 |
| [AAEL003970](https://www.vectorbase.org/aedes_aegypti/Gene/Summary?db=core;g=AAEL003970) | amino acid transporter [Source:VB External Description;Acc:AAEL003970] | -0.29722 | -0.39885 | -0.45437 | -0.82428 |
| [AAEL003971](https://www.vectorbase.org/aedes_aegypti/Gene/Summary?db=core;g=AAEL003971) |  | -0.14293 | -0.09211 | -0.05603 | -0.83171 |
| [AAEL004059](https://www.vectorbase.org/aedes_aegypti/Gene/Summary?db=core;g=AAEL004059) | cystathionine beta-lyase [Source:VB External Description;Acc:AAEL004059] | -0.17558 | -0.18351 | 0.025409 | -0.53786 |
| [AAEL004125](https://www.vectorbase.org/aedes_aegypti/Gene/Summary?db=core;g=AAEL004125) | signal transduction protein lnk-realted [Source:VB External Description;Acc:AAEL004125] | -0.23743 | -0.40207 | -0.36869 | -0.67147 |
| [AAEL004127](https://www.vectorbase.org/aedes_aegypti/Gene/Summary?db=core;g=AAEL004127) | acyl-coa dehydrogenase [Source:VB External Description;Acc:AAEL004127] | 0.02431 | -0.18921 | -0.32732 | -0.62877 |
| [AAEL004138](https://www.vectorbase.org/aedes_aegypti/Gene/Summary?db=core;g=AAEL004138) | signal peptide peptidase [Source:VB External Description;Acc:AAEL004138] | -0.06425 | -0.1182 | -0.28479 | -0.9159 |
| [AAEL004242](https://www.vectorbase.org/aedes_aegypti/Gene/Summary?db=core;g=AAEL004242) | juvenile hormone-inducible protein, putative [Source:VB External Description;Acc:AAEL004242] | -0.09667 | -0.29469 | -0.47532 | -1.11157 |
| [AAEL004356](https://www.vectorbase.org/aedes_aegypti/Gene/Summary?db=core;g=AAEL004356) |  | -0.1965 | -0.49488 | -0.57485 | -0.98833 |
| [AAEL004418](https://www.vectorbase.org/aedes_aegypti/Gene/Summary?db=core;g=AAEL004418) |  | -0.49688 | -0.71852 | -0.92778 | -1.30754 |
| [AAEL004489](https://www.vectorbase.org/aedes_aegypti/Gene/Summary?db=core;g=AAEL004489) |  | 0.097327 | -0.11934 | -0.21939 | -0.70393 |
| [AAEL004542](https://www.vectorbase.org/aedes_aegypti/Gene/Summary?db=core;g=AAEL004542) | ceramide kinase [Source:VB External Description;Acc:AAEL004542] | -0.23429 | -0.18318 | -0.30749 | -0.76304 |
| [AAEL004579](https://www.vectorbase.org/aedes_aegypti/Gene/Summary?db=core;g=AAEL004579) | short-chain dehydrogenase [Source:VB External Description;Acc:AAEL004579] | -0.05625 | -0.07866 | 0.342781 | -0.64191 |
| [AAEL004582](https://www.vectorbase.org/aedes_aegypti/Gene/Summary?db=core;g=AAEL004582) | beta-galactosidase [Source:VB External Description;Acc:AAEL004582] | -1.1624 | -1.35704 | -1.23825 | -1.83938 |
| [AAEL004634](https://www.vectorbase.org/aedes_aegypti/Gene/Summary?db=core;g=AAEL004634) | condensin [Source:VB External Description;Acc:AAEL004634] | 0.02865 | -0.40511 | -0.23326 | -0.6885 |
| [AAEL004639](https://www.vectorbase.org/aedes_aegypti/Gene/Summary?db=core;g=AAEL004639) | histone deacetylase [Source:VB External Description;Acc:AAEL004639] | -0.08099 | -0.49743 | -0.50219 | -0.84914 |
| [AAEL004658](https://www.vectorbase.org/aedes_aegypti/Gene/Summary?db=core;g=AAEL004658) |  | 0.154835 | 0.105873 | -0.10964 | -0.91041 |
| [AAEL004676](https://www.vectorbase.org/aedes_aegypti/Gene/Summary?db=core;g=AAEL004676) | (s)-2-hydroxy-acid oxidase [Source:VB External Description;Acc:AAEL004676] | -0.32613 | -0.29538 | -0.51649 | -0.67647 |
| [AAEL004687](https://www.vectorbase.org/aedes_aegypti/Gene/Summary?db=core;g=AAEL004687) | otopetrin [Source:VB External Description;Acc:AAEL004687] | -0.01048 | -0.38087 | -0.4893 | -0.97704 |
| [AAEL004692](https://www.vectorbase.org/aedes_aegypti/Gene/Summary?db=core;g=AAEL004692) | otopetrin [Source:VB External Description;Acc:AAEL004692] | -0.16142 | -0.2643 | -0.55148 | -0.7679 |
| [AAEL004707](https://www.vectorbase.org/aedes_aegypti/Gene/Summary?db=core;g=AAEL004707) |  | -0.26085 | -0.265 | -0.38925 | -0.53468 |
| [AAEL004710](https://www.vectorbase.org/aedes_aegypti/Gene/Summary?db=core;g=AAEL004710) | spingomyelin synthetase [Source:VB External Description;Acc:AAEL004710] | 0.04507 | 0.009309 | -0.1413 | -0.59037 |
| [AAEL004814](https://www.vectorbase.org/aedes_aegypti/Gene/Summary?db=core;g=AAEL004814) | potassium-dependent sodium-calcium exchanger, putative [Source:VB External Description;Acc:AAEL004814] | 0.749638 | -1.01527 | -0.94774 | -1.56851 |
| [AAEL004860](https://www.vectorbase.org/aedes_aegypti/Gene/Summary?db=core;g=AAEL004860) | acireductone dioxygenase [Source:VB External Description;Acc:AAEL004860] | -0.19893 | -0.30097 | -0.13943 | -0.67293 |
| [AAEL004905](https://www.vectorbase.org/aedes_aegypti/Gene/Summary?db=core;g=AAEL004905) | monocarboxylate transporter [Source:VB External Description;Acc:AAEL004905] | -0.05049 | -0.06537 | -0.09887 | -1.00585 |
| [AAEL004930](https://www.vectorbase.org/aedes_aegypti/Gene/Summary?db=core;g=AAEL004930) | carbonic anhydrase [Source:VB External Description;Acc:AAEL004930] | -0.1696 | -0.04247 | -0.01788 | -0.74318 |
| [AAEL004931](https://www.vectorbase.org/aedes_aegypti/Gene/Summary?db=core;g=AAEL004931) | beta-hexosaminidase b [Source:VB External Description;Acc:AAEL004931] | -2.56534 | -2.89749 | -1.32353 | -2.34097 |
| [AAEL005001](https://www.vectorbase.org/aedes_aegypti/Gene/Summary?db=core;g=AAEL005001) | aquaporin [Source:VB External Description;Acc:AAEL005001] | 0.113723 | -0.11068 | 0.126309 | -0.63397 |
| [AAEL005002](https://www.vectorbase.org/aedes_aegypti/Gene/Summary?db=core;g=AAEL005002) | roundabout [Source:VB External Description;Acc:AAEL005002] | -0.16859 | -0.17967 | -0.00044 | -0.5767 |
| [AAEL005019](https://www.vectorbase.org/aedes_aegypti/Gene/Summary?db=core;g=AAEL005019) | lactosylceramide 4-alpha-galactosyltransferase (alpha- 1,4-galactosyltransferase) [Source:VB External Description;Acc:AAEL005019] | -0.20429 | -0.47464 | -0.29396 | -0.92797 |
| [AAEL005047](https://www.vectorbase.org/aedes_aegypti/Gene/Summary?db=core;g=AAEL005047) |  | -0.19805 | -0.7539 | -0.48392 | -0.73244 |
| [AAEL005058](https://www.vectorbase.org/aedes_aegypti/Gene/Summary?db=core;g=AAEL005058) |  | 0.057313 | -0.32287 | -0.20076 | -0.84517 |
| [AAEL005068](https://www.vectorbase.org/aedes_aegypti/Gene/Summary?db=core;g=AAEL005068) | S-phase kinase-associated protein 2 (skp2), putative [Source:VB External Description;Acc:AAEL005068] | 0.135206 | -0.41789 | -0.10123 | -0.7609 |
| [AAEL005101](https://www.vectorbase.org/aedes_aegypti/Gene/Summary?db=core;g=AAEL005101) | Carboxy/choline esterase Alpha Esterase [Source:VB Community Annotation;Acc:AAEL005101] | 0.220916 | 0.297147 | 0.081853 | -0.62652 |
| [AAEL005102](https://www.vectorbase.org/aedes_aegypti/Gene/Summary?db=core;g=AAEL005102) |  | -0.24999 | -0.64209 | -0.27508 | -0.78968 |
| [AAEL005277](https://www.vectorbase.org/aedes_aegypti/Gene/Summary?db=core;g=AAEL005277) |  | -0.24293 | -0.48772 | -0.69892 | -0.66366 |
| [AAEL005289](https://www.vectorbase.org/aedes_aegypti/Gene/Summary?db=core;g=AAEL005289) | ornithine aminotransferase [Source:VB External Description;Acc:AAEL005289] | -0.17541 | -0.12797 | -0.24284 | -1.03621 |
| [AAEL005312](https://www.vectorbase.org/aedes_aegypti/Gene/Summary?db=core;g=AAEL005312) |  | -0.2517 | -0.8321 | -0.64432 | -1.0385 |
| [AAEL005337](https://www.vectorbase.org/aedes_aegypti/Gene/Summary?db=core;g=AAEL005337) | carbonic anhydrase [Source:VB External Description;Acc:AAEL005337] | -2.17688 | -1.8618 | -2.34835 | -2.43847 |
| [AAEL005340](https://www.vectorbase.org/aedes_aegypti/Gene/Summary?db=core;g=AAEL005340) |  | -0.53706 | -1.12194 | -0.75982 | -0.98975 |
| [AAEL005359](https://www.vectorbase.org/aedes_aegypti/Gene/Summary?db=core;g=AAEL005359) | inhibitor of kappab kinase epsilon [Source:VB External Description;Acc:AAEL005359] | -0.2966 | -0.35706 | -0.38285 | -0.56144 |
| [AAEL005366](https://www.vectorbase.org/aedes_aegypti/Gene/Summary?db=core;g=AAEL005366) |  | -0.24898 | -0.25136 | -0.50061 | -1.07089 |
| [AAEL005396](https://www.vectorbase.org/aedes_aegypti/Gene/Summary?db=core;g=AAEL005396) |  | 0.002415 | -0.00432 | -0.11013 | -0.59167 |
| [AAEL005406](https://www.vectorbase.org/aedes_aegypti/Gene/Summary?db=core;g=AAEL005406) |  | -0.17884 | -0.46572 | -0.18414 | -0.70837 |
| [AAEL005439](https://www.vectorbase.org/aedes_aegypti/Gene/Summary?db=core;g=AAEL005439) | mical [Source:VB External Description;Acc:AAEL005439] | -0.48373 | -0.53466 | -0.50179 | -0.76235 |
| [AAEL005441](https://www.vectorbase.org/aedes_aegypti/Gene/Summary?db=core;g=AAEL005441) | heterogeneous nuclear ribonucleoprotein (hnrnp) [Source:VB External Description;Acc:AAEL005441] | -0.18924 | -0.62725 | -0.90086 | -1.08765 |
| [AAEL005476](https://www.vectorbase.org/aedes_aegypti/Gene/Summary?db=core;g=AAEL005476) |  | -0.11752 | -0.36242 | -0.54363 | -1.31138 |
| [AAEL005581](https://www.vectorbase.org/aedes_aegypti/Gene/Summary?db=core;g=AAEL005581) | norepinephrine/norepinephrine transporter [Source:VB External Description;Acc:AAEL005581] | -0.02369 | -0.36308 | -0.90135 | -1.45094 |
| [AAEL005607](https://www.vectorbase.org/aedes_aegypti/Gene/Summary?db=core;g=AAEL005607) | trypsin [Source:VB External Description;Acc:AAEL005607] | -0.4716 | -0.49022 | -0.6493 | -0.60489 |
| [AAEL005676](https://www.vectorbase.org/aedes_aegypti/Gene/Summary?db=core;g=AAEL005676) | adenosine deaminase [Source:VB External Description;Acc:AAEL005676] | -0.24119 | -0.13274 | -0.10985 | -0.69548 |
| [AAEL005694](https://www.vectorbase.org/aedes_aegypti/Gene/Summary?db=core;g=AAEL005694) |  | -0.43706 | -0.56861 | -0.70498 | -1.34925 |
| [AAEL005716](https://www.vectorbase.org/aedes_aegypti/Gene/Summary?db=core;g=AAEL005716) |  | -0.15377 | -0.48016 | -0.41277 | -0.99088 |
| [AAEL005737](https://www.vectorbase.org/aedes_aegypti/Gene/Summary?db=core;g=AAEL005737) |  | 0.068521 | -0.31148 | -0.00367 | -0.93956 |
| [AAEL005754](https://www.vectorbase.org/aedes_aegypti/Gene/Summary?db=core;g=AAEL005754) | cgmp-dependent protein kinase [Source:VB External Description;Acc:AAEL005754] | -0.51131 | -0.60033 | -0.823 | -1.07956 |
| [AAEL005832](https://www.vectorbase.org/aedes_aegypti/Gene/Summary?db=core;g=AAEL005832) | programmed cell death [Source:VB External Description;Acc:AAEL005832] | -0.05579 | 0.030318 | -0.20258 | -0.85749 |
| [AAEL005866](https://www.vectorbase.org/aedes_aegypti/Gene/Summary?db=core;g=AAEL005866) | Succinate dehydrogenase assembly factor 2, mitochondrial Precursor (SDH assembly factor 2)(Succinate dehydrogenase subunit 5, mitochondrial) [Source:VB External Description;Acc:AAEL005866] | -0.05877 | -0.12226 | -0.38123 | -0.7597 |
| [AAEL005921](https://www.vectorbase.org/aedes_aegypti/Gene/Summary?db=core;g=AAEL005921) | D-lactate dehydrognease 2, [Source:VB External Description;Acc:AAEL005921] | -0.06314 | 0.006533 | -0.02613 | -0.56948 |
| [AAEL006027](https://www.vectorbase.org/aedes_aegypti/Gene/Summary?db=core;g=AAEL006027) | lipase [Source:VB External Description;Acc:AAEL006027] | -0.50781 | -1.20422 | -0.97098 | -1.78349 |
| [AAEL006081](https://www.vectorbase.org/aedes_aegypti/Gene/Summary?db=core;g=AAEL006081) | NADPH fad oxidoreductase [Source:VB External Description;Acc:AAEL006081] | -0.12575 | -0.46338 | -0.23737 | -0.5886 |
| [AAEL006127](https://www.vectorbase.org/aedes_aegypti/Gene/Summary?db=core;g=AAEL006127) |  | -0.99267 | -1.2444 | -0.57828 | -2.17457 |
| [AAEL006189](https://www.vectorbase.org/aedes_aegypti/Gene/Summary?db=core;g=AAEL006189) |  | -0.09182 | -0.29883 | -0.47261 | -1.00154 |
| [AAEL006198](https://www.vectorbase.org/aedes_aegypti/Gene/Summary?db=core;g=AAEL006198) |  | 0.398856 | 0.395253 | -0.02917 | -1.00269 |
| [AAEL006216](https://www.vectorbase.org/aedes_aegypti/Gene/Summary?db=core;g=AAEL006216) |  | -0.08472 | -0.06974 | -0.51507 | -0.91964 |
| [AAEL006228](https://www.vectorbase.org/aedes_aegypti/Gene/Summary?db=core;g=AAEL006228) |  | 0.111305 | -0.20763 | -0.10753 | -0.69369 |
| [AAEL006301](https://www.vectorbase.org/aedes_aegypti/Gene/Summary?db=core;g=AAEL006301) | fruitless [Source:VB External Description;Acc:AAEL006301] | 0.004385 | -0.38964 | 0.040439 | -0.82826 |
| [AAEL006346](https://www.vectorbase.org/aedes_aegypti/Gene/Summary?db=core;g=AAEL006346) |  | 0.168291 | 0.035103 | -0.27254 | -1.03865 |
| [AAEL006372](https://www.vectorbase.org/aedes_aegypti/Gene/Summary?db=core;g=AAEL006372) | sulphate transporter [Source:VB External Description;Acc:AAEL006372] | -0.01079 | -0.08115 | -0.2084 | -0.64418 |
| [AAEL006390](https://www.vectorbase.org/aedes_aegypti/Gene/Summary?db=core;g=AAEL006390) | vacuolar proton ATPases [Source:VB External Description;Acc:AAEL006390] | -0.03308 | -0.02993 | -0.03479 | -0.54981 |
| [AAEL006467](https://www.vectorbase.org/aedes_aegypti/Gene/Summary?db=core;g=AAEL006467) | alcohol dehydrogenase [Source:VB External Description;Acc:AAEL006467] | -0.1792 | -0.04155 | 0.127477 | -0.79723 |
| [AAEL006491](https://www.vectorbase.org/aedes_aegypti/Gene/Summary?db=core;g=AAEL006491) | peroxisomal targeting signal 2 receptor [Source:VB External Description;Acc:AAEL006491] | 0.010059 | -0.56167 | -0.46642 | -0.81471 |
| [AAEL006525](https://www.vectorbase.org/aedes_aegypti/Gene/Summary?db=core;g=AAEL006525) | kelch repeat protein [Source:VB External Description;Acc:AAEL006525] | -0.05451 | -0.25315 | -0.18765 | -0.82897 |
| [AAEL006604](https://www.vectorbase.org/aedes_aegypti/Gene/Summary?db=core;g=AAEL006604) |  | -0.3161 | -0.58444 | -0.70822 | -1.53164 |
| [AAEL006625](https://www.vectorbase.org/aedes_aegypti/Gene/Summary?db=core;g=AAEL006625) |  | -0.13601 | -0.57317 | -0.67325 | -0.78067 |
| [AAEL006758](https://www.vectorbase.org/aedes_aegypti/Gene/Summary?db=core;g=AAEL006758) |  | -0.16443 | -0.42215 | 0.194273 | -1.75603 |
| [AAEL006802](https://www.vectorbase.org/aedes_aegypti/Gene/Summary?db=core;g=AAEL006802) | cytochrome P450 [Source:VB Community Annotation;Acc:AAEL006802] | 0.021811 | 0.392338 | -0.10321 | -0.84377 |
| [AAEL006832](https://www.vectorbase.org/aedes_aegypti/Gene/Summary?db=core;g=AAEL006832) | GPCR Frizzled/Smoothened Family [Source:VB Community Annotation;Acc:AAEL006832] | -0.322 | -0.60978 | -0.58882 | -1.15718 |
| [AAEL006843](https://www.vectorbase.org/aedes_aegypti/Gene/Summary?db=core;g=AAEL006843) |  | -0.02475 | -0.3252 | -0.08652 | -0.71043 |
| [AAEL006863](https://www.vectorbase.org/aedes_aegypti/Gene/Summary?db=core;g=AAEL006863) |  | 0.179179 | 0.087521 | -0.45346 | -0.73006 |
| [AAEL006903](https://www.vectorbase.org/aedes_aegypti/Gene/Summary?db=core;g=AAEL006903) | trypsin [Source:VB External Description;Acc:AAEL006903] | -4.08591 | -4.31017 | -3.30253 | -5.99503 |
| [AAEL006915](https://www.vectorbase.org/aedes_aegypti/Gene/Summary?db=core;g=AAEL006915) |  | -0.37573 | -0.24604 | -0.21719 | -0.63344 |
| [AAEL006984](https://www.vectorbase.org/aedes_aegypti/Gene/Summary?db=core;g=AAEL006984) | cytochrome P450 [Source:VB Community Annotation;Acc:AAEL006984] | 0.245465 | 0.301736 | 0.061183 | -0.63271 |
| [AAEL006992](https://www.vectorbase.org/aedes_aegypti/Gene/Summary?db=core;g=AAEL006992) | cytochrome P450 [Source:VB Community Annotation;Acc:AAEL006992] | -0.05774 | -0.08546 | -0.42123 | -0.69397 |
| [AAEL007016](https://www.vectorbase.org/aedes_aegypti/Gene/Summary?db=core;g=AAEL007016) | sodium/solute symporter [Source:VB External Description;Acc:AAEL007016] | -0.12778 | -0.53599 | -0.79272 | -1.37281 |
| [AAEL007028](https://www.vectorbase.org/aedes_aegypti/Gene/Summary?db=core;g=AAEL007028) | ceramidase [Source:VB External Description;Acc:AAEL007028] | -0.07685 | -0.13362 | -0.5167 | -0.97872 |
| [AAEL007136](https://www.vectorbase.org/aedes_aegypti/Gene/Summary?db=core;g=AAEL007136) | sugar transporter [Source:VB External Description;Acc:AAEL007136] | -0.03322 | -0.21612 | -0.29966 | -0.63963 |
| [AAEL007189](https://www.vectorbase.org/aedes_aegypti/Gene/Summary?db=core;g=AAEL007189) | nocturnin [Source:VB External Description;Acc:AAEL007189] | 0.013237 | -0.61565 | -0.33911 | -0.93948 |
| [AAEL007267](https://www.vectorbase.org/aedes_aegypti/Gene/Summary?db=core;g=AAEL007267) | suppressor of ty3 [Source:VB External Description;Acc:AAEL007267] | -0.14244 | -0.26913 | -0.47315 | -0.59724 |
| [AAEL007279](https://www.vectorbase.org/aedes_aegypti/Gene/Summary?db=core;g=AAEL007279) |  | -0.06379 | -0.62386 | -0.95313 | -1.36229 |
| [AAEL007305](https://www.vectorbase.org/aedes_aegypti/Gene/Summary?db=core;g=AAEL007305) |  | -0.68061 | -1.31115 | 0.420985 | -2.08627 |
| [AAEL007375](https://www.vectorbase.org/aedes_aegypti/Gene/Summary?db=core;g=AAEL007375) | pyruvate dehydrogenase [Source:VB External Description;Acc:AAEL007375] | 0.008687 | -0.09036 | -0.45109 | -0.8093 |
| [AAEL007399](https://www.vectorbase.org/aedes_aegypti/Gene/Summary?db=core;g=AAEL007399) |  | -0.26558 | -0.33574 | -0.57365 | -0.62271 |
| [AAEL007416](https://www.vectorbase.org/aedes_aegypti/Gene/Summary?db=core;g=AAEL007416) | cysteine dioxygenase [Source:VB External Description;Acc:AAEL007416] | -0.02658 | 0.220582 | -0.11164 | -0.90597 |
| [AAEL007417](https://www.vectorbase.org/aedes_aegypti/Gene/Summary?db=core;g=AAEL007417) |  | 0.050653 | -0.27403 | -0.40111 | -0.66344 |
| [AAEL007466](https://www.vectorbase.org/aedes_aegypti/Gene/Summary?db=core;g=AAEL007466) | histone-fold protein CHRAC subunit, putative [Source:VB External Description;Acc:AAEL007466] | -0.17113 | -0.33203 | -0.61065 | -0.67787 |
| [AAEL007473](https://www.vectorbase.org/aedes_aegypti/Gene/Summary?db=core;g=AAEL007473) | cytochrome P450 [Source:VB Community Annotation;Acc:AAEL007473] | -0.29892 | -0.3067 | -0.16696 | -0.83678 |
| [AAEL007541](https://www.vectorbase.org/aedes_aegypti/Gene/Summary?db=core;g=AAEL007541) | DNA polymerase delta small subunit [Source:VB External Description;Acc:AAEL007541] | -0.12687 | -0.63205 | -0.27039 | -0.82383 |
| [AAEL007560](https://www.vectorbase.org/aedes_aegypti/Gene/Summary?db=core;g=AAEL007560) | core 1 udp-galactose:n-acetylgalactosamine-alpha-r beta 1,3- galactosyltransferase [Source:VB External Description;Acc:AAEL007560] | -0.18112 | -0.2702 | -0.4897 | -0.54832 |
| [AAEL007633](https://www.vectorbase.org/aedes_aegypti/Gene/Summary?db=core;g=AAEL007633) | dihydropyrimidinase [Source:VB External Description;Acc:AAEL007633] | -0.26299 | -0.40346 | -0.68217 | -1.06367 |
| [AAEL007680](https://www.vectorbase.org/aedes_aegypti/Gene/Summary?db=core;g=AAEL007680) |  | 0.178259 | -0.03696 | -0.12222 | -1.07085 |
| [AAEL007780](https://www.vectorbase.org/aedes_aegypti/Gene/Summary?db=core;g=AAEL007780) |  | 0.139307 | -1.12585 | -0.10103 | -1.70492 |
| [AAEL007802](https://www.vectorbase.org/aedes_aegypti/Gene/Summary?db=core;g=AAEL007802) | multicopper oxidase [Source:VB External Description;Acc:AAEL007802] | -0.19836 | 0.126587 | -0.29229 | -0.85589 |
| [AAEL007808](https://www.vectorbase.org/aedes_aegypti/Gene/Summary?db=core;g=AAEL007808) | cytochrome P450 (CYP4D39) [Source:VB Community Annotation;Acc:AAEL007808] | 0.511279 | -0.55304 | -0.39243 | -1.34891 |
| [AAEL007811](https://www.vectorbase.org/aedes_aegypti/Gene/Summary?db=core;g=AAEL007811) | short-chain dehydrogenase [Source:VB External Description;Acc:AAEL007811] | -0.44017 | -0.88335 | -0.51972 | -1.51411 |
| [AAEL007816](https://www.vectorbase.org/aedes_aegypti/Gene/Summary?db=core;g=AAEL007816) | cytochrome P450 (CYP4D23) [Source:VB Community Annotation;Acc:AAEL007816] | -3.48389258617376e-05 | 0.026376 | 0.053285 | -0.72833 |
| [AAEL007871](https://www.vectorbase.org/aedes_aegypti/Gene/Summary?db=core;g=AAEL007871) | anaphase-promoting complex [Source:VB External Description;Acc:AAEL007871] | -0.31381 | -0.48193 | -0.25883 | -0.65216 |
| [AAEL007959](https://www.vectorbase.org/aedes_aegypti/Gene/Summary?db=core;g=AAEL007959) |  | -0.02965 | -0.19633 | -0.30009 | -0.53235 |
| [AAEL007971](https://www.vectorbase.org/aedes_aegypti/Gene/Summary?db=core;g=AAEL007971) | tyrosine transporter [Source:VB External Description;Acc:AAEL007971] | -0.01952 | -0.13359 | -0.16401 | -0.57382 |
| [AAEL008016](https://www.vectorbase.org/aedes_aegypti/Gene/Summary?db=core;g=AAEL008016) | short-chain dehydrogenase [Source:VB External Description;Acc:AAEL008016] | -0.08678 | 0.157721 | -0.20763 | -0.63409 |
| [AAEL008032](https://www.vectorbase.org/aedes_aegypti/Gene/Summary?db=core;g=AAEL008032) |  | -0.18952 | -0.93512 | -1.00203 | -2.01236 |
| [AAEL008041](https://www.vectorbase.org/aedes_aegypti/Gene/Summary?db=core;g=AAEL008041) | bleomycin hydrolase [Source:VB External Description;Acc:AAEL008041] | -0.11144 | -0.23293 | -0.50363 | -0.87089 |
| [AAEL008049](https://www.vectorbase.org/aedes_aegypti/Gene/Summary?db=core;g=AAEL008049) |  | -0.09529 | -0.16823 | -0.05753 | -0.88138 |
| [AAEL008102](https://www.vectorbase.org/aedes_aegypti/Gene/Summary?db=core;g=AAEL008102) | actin binding protein, putative [Source:VB External Description;Acc:AAEL008102] | -0.29053 | -0.24137 | -0.61292 | -0.69584 |
| [AAEL008136](https://www.vectorbase.org/aedes_aegypti/Gene/Summary?db=core;g=AAEL008136) |  | -0.16257 | -0.26058 | -0.48216 | -0.6888 |
| [AAEL008138](https://www.vectorbase.org/aedes_aegypti/Gene/Summary?db=core;g=AAEL008138) | ABC transporter [Source:VB External Description;Acc:AAEL008138] | -0.18368 | -0.45254 | -0.20188 | -0.91655 |
| [AAEL008144](https://www.vectorbase.org/aedes_aegypti/Gene/Summary?db=core;g=AAEL008144) | AMP dependent ligase [Source:VB External Description;Acc:AAEL008144] | 0.145401 | -0.30817 | -0.2863 | -0.57379 |
| [AAEL008183](https://www.vectorbase.org/aedes_aegypti/Gene/Summary?db=core;g=AAEL008183) | t complex protein [Source:VB External Description;Acc:AAEL008183] | -0.21477 | -0.37906 | -0.65994 | -0.80033 |
| [AAEL008222](https://www.vectorbase.org/aedes_aegypti/Gene/Summary?db=core;g=AAEL008222) | lipase [Source:VB External Description;Acc:AAEL008222] | -0.33192 | -0.32746 | -0.81794 | -0.9602 |
| [AAEL008242](https://www.vectorbase.org/aedes_aegypti/Gene/Summary?db=core;g=AAEL008242) | hook protein [Source:VB External Description;Acc:AAEL008242] | -0.07309 | -0.08802 | -0.36661 | -0.58417 |
| [AAEL008249](https://www.vectorbase.org/aedes_aegypti/Gene/Summary?db=core;g=AAEL008249) |  | -0.14836 | -0.06877 | -0.02219 | -0.5443 |
| [AAEL008262](https://www.vectorbase.org/aedes_aegypti/Gene/Summary?db=core;g=AAEL008262) | ppa (fragment) [Source:VB External Description;Acc:AAEL008262] | -0.11187 | 0.059488 | -0.58817 | -1.11923 |
| [AAEL008279](https://www.vectorbase.org/aedes_aegypti/Gene/Summary?db=core;g=AAEL008279) |  | 0.092542 | 0.128963 | -0.69182 | -0.90912 |
| [AAEL008347](https://www.vectorbase.org/aedes_aegypti/Gene/Summary?db=core;g=AAEL008347) | monocarboxylate transporter [Source:VB External Description;Acc:AAEL008347] | -0.37659 | -0.60689 | -0.93764 | -0.99356 |
| [AAEL008386](https://www.vectorbase.org/aedes_aegypti/Gene/Summary?db=core;g=AAEL008386) | ATP-binding cassette sub-family A member 3, putative [Source:VB External Description;Acc:AAEL008386] | -0.22008 | -0.24152 | -0.56501 | -0.89531 |
| [AAEL008395](https://www.vectorbase.org/aedes_aegypti/Gene/Summary?db=core;g=AAEL008395) |  | 0.025346 | -0.18174 | -0.297 | -1.08961 |
| [AAEL008430](https://www.vectorbase.org/aedes_aegypti/Gene/Summary?db=core;g=AAEL008430) |  | -1.77621 | -0.84348 | -3.08726 | -4.11714 |
| [AAEL008472](https://www.vectorbase.org/aedes_aegypti/Gene/Summary?db=core;g=AAEL008472) | glycerol kinase [Source:VB External Description;Acc:AAEL008472] | -0.20102 | -0.26932 | -0.49815 | -0.75985 |
| [AAEL008519](https://www.vectorbase.org/aedes_aegypti/Gene/Summary?db=core;g=AAEL008519) | DNA replication factor Cdt1 [Source:VB External Description;Acc:AAEL008519] | -0.05941 | -0.4201 | -0.35896 | -0.87695 |
| [AAEL008560](https://www.vectorbase.org/aedes_aegypti/Gene/Summary?db=core;g=AAEL008560) | glucosyl/glucuronosyl transferases [Source:VB External Description;Acc:AAEL008560] | -0.03028 | 0.107287 | -0.7278 | -0.81079 |
| [AAEL008563](https://www.vectorbase.org/aedes_aegypti/Gene/Summary?db=core;g=AAEL008563) |  | -0.07618 | 0.155971 | -0.06009 | -0.76455 |
| [AAEL008587](https://www.vectorbase.org/aedes_aegypti/Gene/Summary?db=core;g=AAEL008587) | glutamate receptor, ionotropic, N-methyl d-aspartate [Source:VB External Description;Acc:AAEL008587] | 0.379711 | 0.172815 | -0.60528 | -0.93614 |
| [AAEL008624](https://www.vectorbase.org/aedes_aegypti/Gene/Summary?db=core;g=AAEL008624) | ABC transporter [Source:VB External Description;Acc:AAEL008624] | -0.23707 | -0.45761 | -1.07172 | -1.05391 |
| [AAEL008629](https://www.vectorbase.org/aedes_aegypti/Gene/Summary?db=core;g=AAEL008629) | ABC transporter [Source:VB External Description;Acc:AAEL008629] | -0.36149 | -0.25249 | -0.78863 | -0.80893 |
| [AAEL008693](https://www.vectorbase.org/aedes_aegypti/Gene/Summary?db=core;g=AAEL008693) | cation efflux protein/ zinc transporter [Source:VB External Description;Acc:AAEL008693] | -0.13385 | -0.15022 | -0.17325 | -0.76245 |
| [AAEL008701](https://www.vectorbase.org/aedes_aegypti/Gene/Summary?db=core;g=AAEL008701) | myoinositol oxygenase [Source:VB External Description;Acc:AAEL008701] | -0.42355 | -0.6314 | -0.71678 | -1.24651 |
| [AAEL008841](https://www.vectorbase.org/aedes_aegypti/Gene/Summary?db=core;g=AAEL008841) | acyl-CoA oxidase [Source:VB External Description;Acc:AAEL008841] | -0.13129 | -0.18764 | -0.50305 | -0.83198 |
| [AAEL008878](https://www.vectorbase.org/aedes_aegypti/Gene/Summary?db=core;g=AAEL008878) | diacylglycerol o-acyltransferase [Source:VB External Description;Acc:AAEL008878] | -0.23344 | -0.31177 | -0.03522 | -0.65324 |
| [AAEL008879](https://www.vectorbase.org/aedes_aegypti/Gene/Summary?db=core;g=AAEL008879) | Kynurenine 3-monooxygenase (EC 1.14.13.9)(Kynurenine 3-hydroxylase) [Source:VB External Description;Acc:AAEL008879] | -0.1057 | -0.42692 | 0.018243 | -1.1007 |
| [AAEL008881](https://www.vectorbase.org/aedes_aegypti/Gene/Summary?db=core;g=AAEL008881) | RHC18, putative [Source:VB External Description;Acc:AAEL008881] | 0.135521 | 0.168551 | -0.00785 | -0.71058 |
| [AAEL008898](https://www.vectorbase.org/aedes_aegypti/Gene/Summary?db=core;g=AAEL008898) | sulfotransferase (sult) [Source:VB External Description;Acc:AAEL008898] | 0.052179 | -0.26321 | -0.25376 | -0.66864 |
| [AAEL008939](https://www.vectorbase.org/aedes_aegypti/Gene/Summary?db=core;g=AAEL008939) |  | -0.16475 | -0.1445 | -0.04363 | -0.6767 |
| [AAEL008961](https://www.vectorbase.org/aedes_aegypti/Gene/Summary?db=core;g=AAEL008961) |  | 0.235684 | -0.26213 | -0.11366 | -0.91509 |
| [AAEL009017](https://www.vectorbase.org/aedes_aegypti/Gene/Summary?db=core;g=AAEL009017) | glutathione transferase [Source:VB Community Annotation;Acc:AAEL009017] | -0.37047 | -0.347 | -0.40601 | -0.65586 |
| [AAEL009020](https://www.vectorbase.org/aedes_aegypti/Gene/Summary?db=core;g=AAEL009020) | glutathione transferase [Source:VB Community Annotation;Acc:AAEL009020] | -0.30818 | -0.47046 | -0.26104 | -0.73207 |
| [AAEL009029](https://www.vectorbase.org/aedes_aegypti/Gene/Summary?db=core;g=AAEL009029) | aldehyde dehydrogenase [Source:VB Community Annotation;Acc:AAEL009029] | -0.21372 | -0.18216 | -0.29637 | -0.58226 |
| [AAEL009036](https://www.vectorbase.org/aedes_aegypti/Gene/Summary?db=core;g=AAEL009036) |  | -0.35548 | -0.61047 | -0.58372 | -0.60449 |
| [AAEL009120](https://www.vectorbase.org/aedes_aegypti/Gene/Summary?db=core;g=AAEL009120) | cytochrome P450 [Source:VB Community Annotation;Acc:AAEL009120] | 0.211506 | 0.046154 | -0.23701 | -0.70309 |
| [AAEL009198](https://www.vectorbase.org/aedes_aegypti/Gene/Summary?db=core;g=AAEL009198) |  | -0.18421 | -0.30558 | -0.38184 | -0.72833 |
| [AAEL009206](https://www.vectorbase.org/aedes_aegypti/Gene/Summary?db=core;g=AAEL009206) | organic cation transporter [Source:VB External Description;Acc:AAEL009206] | -0.04713 | -0.1117 | -0.25107 | -0.77601 |
| [AAEL009208](https://www.vectorbase.org/aedes_aegypti/Gene/Summary?db=core;g=AAEL009208) |  | -0.13331 | -0.55862 | -0.38421 | -0.70664 |
| [AAEL009250](https://www.vectorbase.org/aedes_aegypti/Gene/Summary?db=core;g=AAEL009250) |  | -0.64674 | -1.233 | -1.02766 | -1.33875 |
| [AAEL009330](https://www.vectorbase.org/aedes_aegypti/Gene/Summary?db=core;g=AAEL009330) | carbonic anhydrase II, putative [Source:VB External Description;Acc:AAEL009330] | -0.175 | -0.40124 | -0.35987 | -0.87792 |
| [AAEL009342](https://www.vectorbase.org/aedes_aegypti/Gene/Summary?db=core;g=AAEL009342) |  | 0.060085 | -0.1115 | -0.28415 | -0.57274 |
| [AAEL009420](https://www.vectorbase.org/aedes_aegypti/Gene/Summary?db=core;g=AAEL009420) | Class B Scavenger Receptor (CD36 domain). [Source:VB Community Annotation;Acc:AAEL009420] | -0.08698 | -0.19662 | -0.28349 | -0.80227 |
| [AAEL009423](https://www.vectorbase.org/aedes_aegypti/Gene/Summary?db=core;g=AAEL009423) | Class B Scavenger Receptor (CD36 domain). [Source:VB Community Annotation;Acc:AAEL009423] | -0.01309 | -0.31393 | -0.18045 | -0.64915 |
| [AAEL009513](https://www.vectorbase.org/aedes_aegypti/Gene/Summary?db=core;g=AAEL009513) | adenylate cyclase [Source:VB External Description;Acc:AAEL009513] | -1.0321 | -0.56061 | -0.45289 | -1.63192 |
| [AAEL009588](https://www.vectorbase.org/aedes_aegypti/Gene/Summary?db=core;g=AAEL009588) | expressed protein (HR3) [Source:VB External Description;Acc:AAEL009588] | 0.498108 | -0.5818 | -4.91803 | -0.60465 |
| [AAEL009598](https://www.vectorbase.org/aedes_aegypti/Gene/Summary?db=core;g=AAEL009598) |  | -0.42176 | -0.82457 | -1.04115 | -0.40648 |
| [AAEL009664](https://www.vectorbase.org/aedes_aegypti/Gene/Summary?db=core;g=AAEL009664) | aldehyde dehydrogenase [Source:VB External Description;Acc:AAEL009664] | -0.16277 | 0.132832 | -0.34821 | -0.75569 |
| [AAEL009676](https://www.vectorbase.org/aedes_aegypti/Gene/Summary?db=core;g=AAEL009676) | glyoxylate/hydroxypyruvate reductase [Source:VB External Description;Acc:AAEL009676] | -0.36725 | -0.63196 | -0.3077 | -0.65038 |
| [AAEL009728](https://www.vectorbase.org/aedes_aegypti/Gene/Summary?db=core;g=AAEL009728) |  | -0.19802 | -0.85798 | -0.63898 | -1.08391 |
| [AAEL009731](https://www.vectorbase.org/aedes_aegypti/Gene/Summary?db=core;g=AAEL009731) | epsilon-trimethyllysine 2-oxoglutarate dioxygenase [Source:VB External Description;Acc:AAEL009731] | -0.11936 | -0.32525 | -0.13136 | -0.7139 |
| [AAEL009748](https://www.vectorbase.org/aedes_aegypti/Gene/Summary?db=core;g=AAEL009748) | 2-hydroxyphytanoyl-coa lyase [Source:VB External Description;Acc:AAEL009748] | -0.00645 | -0.05653 | -0.05913 | -1.07437 |
| [AAEL009756](https://www.vectorbase.org/aedes_aegypti/Gene/Summary?db=core;g=AAEL009756) |  | 0.183522 | 0.088624 | -0.33151 | -0.89982 |
| [AAEL009838](https://www.vectorbase.org/aedes_aegypti/Gene/Summary?db=core;g=AAEL009838) | glycogen debranching enzyme [Source:VB External Description;Acc:AAEL009838] | -0.03098 | 0.112251 | -0.22018 | -0.73323 |
| [AAEL009842](https://www.vectorbase.org/aedes_aegypti/Gene/Summary?db=core;g=AAEL009842) | galectin [Source:VB Community Annotation;Acc:AAEL009842] | -0.19583 | 0.436756 | 0.376725 | -0.80425 |
| [AAEL009881](https://www.vectorbase.org/aedes_aegypti/Gene/Summary?db=core;g=AAEL009881) | dynein heavy chain [Source:VB External Description;Acc:AAEL009881] | -0.75883 | -1.09124 | -0.98174 | -1.45132 |
| [AAEL009968](https://www.vectorbase.org/aedes_aegypti/Gene/Summary?db=core;g=AAEL009968) |  | -0.07355 | -0.53473 | -0.46148 | -1.02879 |
| [AAEL009985](https://www.vectorbase.org/aedes_aegypti/Gene/Summary?db=core;g=AAEL009985) |  | -2.70714 | -0.66894 | -4.31389 | -2.81368 |
| [AAEL010037](https://www.vectorbase.org/aedes_aegypti/Gene/Summary?db=core;g=AAEL010037) | phosphoglucomutase [Source:VB External Description;Acc:AAEL010037] | -0.35363 | -0.20734 | -0.80181 | -0.86786 |
| [AAEL010078](https://www.vectorbase.org/aedes_aegypti/Gene/Summary?db=core;g=AAEL010078) |  | 0.045794 | 0.14728 | -0.15556 | -0.52346 |
| [AAEL010154](https://www.vectorbase.org/aedes_aegypti/Gene/Summary?db=core;g=AAEL010154) | cytochrome P450 [Source:VB Community Annotation;Acc:AAEL010154] | 0.11745 | -0.07865 | -0.14147 | -0.54738 |
| [AAEL010284](https://www.vectorbase.org/aedes_aegypti/Gene/Summary?db=core;g=AAEL010284) | aliphatic nitrilase, putative [Source:VB External Description;Acc:AAEL010284] | -0.15855 | -0.40143 | -0.33074 | -0.97788 |
| [AAEL010337](https://www.vectorbase.org/aedes_aegypti/Gene/Summary?db=core;g=AAEL010337) | CRAL/TRIO domain-containing protein [Source:VB External Description;Acc:AAEL010337] | 0.097557 | -0.72747 | -0.30973 | -1.2862 |
| [AAEL010478](https://www.vectorbase.org/aedes_aegypti/Gene/Summary?db=core;g=AAEL010478) | sugar transporter [Source:VB External Description;Acc:AAEL010478] | -0.24226 | -1.46054 | -1.26192 | -1.46419 |
| [AAEL010590](https://www.vectorbase.org/aedes_aegypti/Gene/Summary?db=core;g=AAEL010590) | aldose-1-epimerase [Source:VB External Description;Acc:AAEL010590] | -0.01055 | -0.60468 | -0.37772 | -0.61958 |
| [AAEL010602](https://www.vectorbase.org/aedes_aegypti/Gene/Summary?db=core;g=AAEL010602) | starch branching enzyme ii [Source:VB External Description;Acc:AAEL010602] | -0.15837 | -0.28477 | -0.59344 | -0.74329 |
| [AAEL010619](https://www.vectorbase.org/aedes_aegypti/Gene/Summary?db=core;g=AAEL010619) |  | -0.21504 | -0.40414 | -0.55116 | -0.90426 |
| [AAEL010656](https://www.vectorbase.org/aedes_aegypti/Gene/Summary?db=core;g=AAEL010656) | leucine-rich immune protein (Short) [Source:VB Community Annotation;Acc:AAEL010656] | -0.57399 | -0.10298 | -1.03642 | -0.41052 |
| [AAEL010753](https://www.vectorbase.org/aedes_aegypti/Gene/Summary?db=core;g=AAEL010753) |  | 0.02612 | -0.43316 | -0.63234 | -0.92767 |
| [AAEL010795](https://www.vectorbase.org/aedes_aegypti/Gene/Summary?db=core;g=AAEL010795) |  | -0.00747 | -0.10232 | -0.20775 | -0.51882 |
| [AAEL010838](https://www.vectorbase.org/aedes_aegypti/Gene/Summary?db=core;g=AAEL010838) |  | 0.176705 | -0.29689 | -0.20872 | -0.80328 |
| [AAEL010946](https://www.vectorbase.org/aedes_aegypti/Gene/Summary?db=core;g=AAEL010946) | cytochrome P450 (CYP314A1) [Source:VB Community Annotation;Acc:AAEL010946] | -0.15788 | -0.41283 | -0.54693 | -1.18527 |
| [AAEL010960](https://www.vectorbase.org/aedes_aegypti/Gene/Summary?db=core;g=AAEL010960) | xaa-pro dipeptidase app(e.coli) [Source:VB External Description;Acc:AAEL010960] | -0.0715 | -0.62432 | -0.5309 | -1.04109 |
| [AAEL010986](https://www.vectorbase.org/aedes_aegypti/Gene/Summary?db=core;g=AAEL010986) | apyrase, putative [Source:VB External Description;Acc:AAEL010986] | -0.11166 | -0.24418 | 0.061899 | -0.59059 |
| [AAEL011050](https://www.vectorbase.org/aedes_aegypti/Gene/Summary?db=core;g=AAEL011050) |  | -0.01847 | -0.35484 | -0.39922 | -0.64035 |
| [AAEL011062](https://www.vectorbase.org/aedes_aegypti/Gene/Summary?db=core;g=AAEL011062) |  | -0.41412 | -0.51444 | -0.86097 | -0.41428 |
| [AAEL011082](https://www.vectorbase.org/aedes_aegypti/Gene/Summary?db=core;g=AAEL011082) |  | 0.175718 | -0.09496 | -0.19776 | -0.81898 |
| [AAEL011112](https://www.vectorbase.org/aedes_aegypti/Gene/Summary?db=core;g=AAEL011112) | alcohol dehydrogenase [Source:VB External Description;Acc:AAEL011112] | -0.46022 | -0.22034 | -0.58368 | -1.17522 |
| [AAEL011187](https://www.vectorbase.org/aedes_aegypti/Gene/Summary?db=core;g=AAEL011187) | U520 [Source:VB External Description;Acc:AAEL011187] | -0.19948 | -0.48739 | -0.51812 | -0.68026 |
| [AAEL011210](https://www.vectorbase.org/aedes_aegypti/Gene/Summary?db=core;g=AAEL011210) |  | -0.30312 | -0.80426 | -0.82229 | -0.83187 |
| [AAEL011213](https://www.vectorbase.org/aedes_aegypti/Gene/Summary?db=core;g=AAEL011213) |  | -0.28764 | -0.48884 | -0.46834 | -0.70591 |
| [AAEL011319](https://www.vectorbase.org/aedes_aegypti/Gene/Summary?db=core;g=AAEL011319) |  | -0.2502 | -0.57128 | -0.6379 | -1.27931 |
| [AAEL011323](https://www.vectorbase.org/aedes_aegypti/Gene/Summary?db=core;g=AAEL011323) | Hepatocyte nuclear factor 4 isoform A nuclear receptor [Source:VB Community Annotation;Acc:AAEL011323] | 0.00937 | 0.107251 | 0.050325 | -0.62767 |
| [AAEL011341](https://www.vectorbase.org/aedes_aegypti/Gene/Summary?db=core;g=AAEL011341) | apyrase, putative [Source:VB External Description;Acc:AAEL011341] | -0.13821 | -0.36616 | -0.0488 | -0.8618 |
| [AAEL011368](https://www.vectorbase.org/aedes_aegypti/Gene/Summary?db=core;g=AAEL011368) | sugar transporter [Source:VB External Description;Acc:AAEL011368] | 0.149619 | -0.52382 | -0.08306 | -0.90418 |
| [AAEL011369](https://www.vectorbase.org/aedes_aegypti/Gene/Summary?db=core;g=AAEL011369) | endothelin-converting enzyme [Source:VB External Description;Acc:AAEL011369] | 0.216157 | 0.106796 | -0.12031 | -0.72079 |
| [AAEL011412](https://www.vectorbase.org/aedes_aegypti/Gene/Summary?db=core;g=AAEL011412) |  | -0.18868 | -0.35197 | -0.55778 | -0.6893 |
| [AAEL011414](https://www.vectorbase.org/aedes_aegypti/Gene/Summary?db=core;g=AAEL011414) | high mobility group non-histone protein, putative [Source:VB External Description;Acc:AAEL011414] | 0.132054 | 0.122535 | -1.10977 | -0.93475 |
| [AAEL011519](https://www.vectorbase.org/aedes_aegypti/Gene/Summary?db=core;g=AAEL011519) | sucrose transport protein [Source:VB External Description;Acc:AAEL011519] | 0.27368 | 0.225674 | 0.306992 | -0.98411 |
| [AAEL011550](https://www.vectorbase.org/aedes_aegypti/Gene/Summary?db=core;g=AAEL011550) | metalloproteinase, putative [Source:VB External Description;Acc:AAEL011550] | -0.1622 | -0.62127 | -0.36802 | -0.7536 |
| [AAEL011636](https://www.vectorbase.org/aedes_aegypti/Gene/Summary?db=core;g=AAEL011636) |  | 0.080954 | -0.2878 | -0.13676 | -0.82902 |
| [AAEL011662](https://www.vectorbase.org/aedes_aegypti/Gene/Summary?db=core;g=AAEL011662) | plasma glutamate carboxypeptidase [Source:VB External Description;Acc:AAEL011662] | -0.30138 | -0.20085 | -0.22628 | -0.72184 |
| [AAEL011676](https://www.vectorbase.org/aedes_aegypti/Gene/Summary?db=core;g=AAEL011676) | AMP dependent coa ligase [Source:VB External Description;Acc:AAEL011676] | -0.02451 | -0.36857 | -0.20291 | -0.96593 |
| [AAEL011677](https://www.vectorbase.org/aedes_aegypti/Gene/Summary?db=core;g=AAEL011677) | AMP dependent coa ligase [Source:VB External Description;Acc:AAEL011677] | -0.03765 | -0.12103 | -0.41317 | -1.04913 |
| [AAEL011689](https://www.vectorbase.org/aedes_aegypti/Gene/Summary?db=core;g=AAEL011689) | acyl carrier protein precursor, putative [Source:VB External Description;Acc:AAEL011689] | -0.22886 | -0.06842 | -0.24536 | -0.65715 |
| [AAEL011799](https://www.vectorbase.org/aedes_aegypti/Gene/Summary?db=core;g=AAEL011799) |  | -0.35469 | -0.60738 | -0.46441 | -0.8487 |
| [AAEL011822](https://www.vectorbase.org/aedes_aegypti/Gene/Summary?db=core;g=AAEL011822) | gamma glutamyl transpeptidases [Source:VB External Description;Acc:AAEL011822] | -0.24312 | -0.84219 | -0.8173 | -1.72526 |
| [AAEL011824](https://www.vectorbase.org/aedes_aegypti/Gene/Summary?db=core;g=AAEL011824) | gamma glutamyl transpeptidases [Source:VB External Description;Acc:AAEL011824] | -0.21853 | -0.19847 | 0.049073 | -0.70682 |
| [AAEL011852](https://www.vectorbase.org/aedes_aegypti/Gene/Summary?db=core;g=AAEL011852) |  | -0.38966 | -0.66161 | -0.67416 | -0.81796 |
| [AAEL011853](https://www.vectorbase.org/aedes_aegypti/Gene/Summary?db=core;g=AAEL011853) |  | -0.12395 | -0.40588 | -0.54929 | -1.04147 |
| [AAEL011910](https://www.vectorbase.org/aedes_aegypti/Gene/Summary?db=core;g=AAEL011910) |  | 0.139337 | -0.03941 | -0.25997 | -0.85377 |
| [AAEL011913](https://www.vectorbase.org/aedes_aegypti/Gene/Summary?db=core;g=AAEL011913) | serine-type enodpeptidase, [Source:VB External Description;Acc:AAEL011913] | -0.54474 | -0.6687 | -0.6073 | -0.64417 |
| [AAEL011919](https://www.vectorbase.org/aedes_aegypti/Gene/Summary?db=core;g=AAEL011919) | serine-type enodpeptidase, [Source:VB External Description;Acc:AAEL011919] | -0.49475 | -0.74781 | -0.50905 | -0.57476 |
| [AAEL011928](https://www.vectorbase.org/aedes_aegypti/Gene/Summary?db=core;g=AAEL011928) |  | -0.20078 | -0.43559 | -0.7156 | -0.76056 |
| [AAEL011929](https://www.vectorbase.org/aedes_aegypti/Gene/Summary?db=core;g=AAEL011929) | serine-type enodpeptidase, [Source:VB External Description;Acc:AAEL011929] | -0.6834 | -0.7211 | -0.88884 | -0.45013 |
| [AAEL012031](https://www.vectorbase.org/aedes_aegypti/Gene/Summary?db=core;g=AAEL012031) |  | -0.03051 | -0.16172 | -0.53564 | -0.65298 |
| [AAEL012036](https://www.vectorbase.org/aedes_aegypti/Gene/Summary?db=core;g=AAEL012036) | sulphate transporter [Source:VB External Description;Acc:AAEL012036] | -0.06699 | -0.29016 | -0.50864 | -0.85464 |
| [AAEL012041](https://www.vectorbase.org/aedes_aegypti/Gene/Summary?db=core;g=AAEL012041) | sulphate transporter [Source:VB External Description;Acc:AAEL012041] | -0.49912 | -0.55185 | -0.92744 | -1.18886 |
| [AAEL012044](https://www.vectorbase.org/aedes_aegypti/Gene/Summary?db=core;g=AAEL012044) | sugar transporter [Source:VB External Description;Acc:AAEL012044] | -0.17967 | -0.23326 | -0.33584 | -0.9741 |
| [AAEL012046](https://www.vectorbase.org/aedes_aegypti/Gene/Summary?db=core;g=AAEL012046) | flotillin-1 [Source:VB External Description;Acc:AAEL012046] | -0.1288 | -0.76028 | -0.54069 | -1.29822 |
| [AAEL012117](https://www.vectorbase.org/aedes_aegypti/Gene/Summary?db=core;g=AAEL012117) | mitochondrial carrier protein [Source:VB External Description;Acc:AAEL012117] | -0.08586 | -0.14365 | -0.39203 | -0.73343 |
| [AAEL012138](https://www.vectorbase.org/aedes_aegypti/Gene/Summary?db=core;g=AAEL012138) | 24-dehydrocholesterol reductase [Source:VB External Description;Acc:AAEL012138] | -0.30414 | -0.50905 | -0.47431 | -0.73445 |
| [AAEL012178](https://www.vectorbase.org/aedes_aegypti/Gene/Summary?db=core;g=AAEL012178) |  | -0.87476 | -0.62535 | -1.53842 | -2.66798 |
| [AAEL012300](https://www.vectorbase.org/aedes_aegypti/Gene/Summary?db=core;g=AAEL012300) |  | 0.235897 | -0.26795 | -0.3229 | -0.73613 |
| [AAEL012305](https://www.vectorbase.org/aedes_aegypti/Gene/Summary?db=core;g=AAEL012305) | hect E3 ubiquitin ligase [Source:VB External Description;Acc:AAEL012305] | -0.04732 | -0.17152 | -0.53471 | -1.00705 |
| [AAEL012337](https://www.vectorbase.org/aedes_aegypti/Gene/Summary?db=core;g=AAEL012337) | goliath E3 ubiquitin ligase [Source:VB External Description;Acc:AAEL012337] | -0.28666 | -0.22831 | -0.29397 | -1.14811 |
| [AAEL012340](https://www.vectorbase.org/aedes_aegypti/Gene/Summary?db=core;g=AAEL012340) | lipase 1 precursor [Source:VB External Description;Acc:AAEL012340] | -3.57286 | -3.89265 | -3.5036 | -3.38367 |
| [AAEL012361](https://www.vectorbase.org/aedes_aegypti/Gene/Summary?db=core;g=AAEL012361) |  | -0.54709 | -0.36587 | 0.057164 | -1.14193 |
| [AAEL012398](https://www.vectorbase.org/aedes_aegypti/Gene/Summary?db=core;g=AAEL012398) |  | -0.25068 | -0.38905 | -0.32341 | -0.71548 |
| [AAEL012427](https://www.vectorbase.org/aedes_aegypti/Gene/Summary?db=core;g=AAEL012427) |  | -0.02253 | -0.19888 | -0.16174 | -0.57177 |
| [AAEL012474](https://www.vectorbase.org/aedes_aegypti/Gene/Summary?db=core;g=AAEL012474) |  | -0.22176 | 0.005257 | -0.23734 | -0.67667 |
| [AAEL012491](https://www.vectorbase.org/aedes_aegypti/Gene/Summary?db=core;g=AAEL012491) | cytochrome P450 (CYP6P12) [Source:VB Community Annotation;Acc:AAEL012491] | -0.25603 | -0.3015 | -0.73236 | -1.11764 |
| [AAEL012492](https://www.vectorbase.org/aedes_aegypti/Gene/Summary?db=core;g=AAEL012492) | cytochrome P450 (CYP6AA5) [Source:VB Community Annotation;Acc:AAEL012492] | 0.022179 | -0.43568 | -0.4835 | -0.89004 |
| [AAEL012494](https://www.vectorbase.org/aedes_aegypti/Gene/Summary?db=core;g=AAEL012494) | cytochrome P450 (CYP6BZ1) [Source:VB Community Annotation;Acc:AAEL012494] | -0.11684 | -0.47347 | -0.6898 | -0.99976 |
| [AAEL012553](https://www.vectorbase.org/aedes_aegypti/Gene/Summary?db=core;g=AAEL012553) | JAKSTAT pathway signalling Janus Kinase Hopscotch. [Source:VB Community Annotation;Acc:AAEL012553] | -0.01989 | -0.09501 | -0.42433 | -1.0486 |
| [AAEL012563](https://www.vectorbase.org/aedes_aegypti/Gene/Summary?db=core;g=AAEL012563) | lumbrokinase-3(1) precursor, putative [Source:VB External Description;Acc:AAEL012563] | 0.135819 | -0.28184 | 0.259168 | -0.73092 |
| [AAEL012616](https://www.vectorbase.org/aedes_aegypti/Gene/Summary?db=core;g=AAEL012616) | NADP transhydrogenase [Source:VB External Description;Acc:AAEL012616] | -0.20725 | -0.21217 | -0.67038 | -0.94842 |
| [AAEL012622](https://www.vectorbase.org/aedes_aegypti/Gene/Summary?db=core;g=AAEL012622) |  | -0.09429 | -0.23605 | -0.46004 | -0.65967 |
| [AAEL012644](https://www.vectorbase.org/aedes_aegypti/Gene/Summary?db=core;g=AAEL012644) |  | 0.336891 | -1.17416 | -0.87178 | -2.13166 |
| [AAEL012655](https://www.vectorbase.org/aedes_aegypti/Gene/Summary?db=core;g=AAEL012655) | sugar transporter [Source:VB External Description;Acc:AAEL012655] | -0.13247 | -0.03015 | -0.25063 | -0.83653 |
| [AAEL012783](https://www.vectorbase.org/aedes_aegypti/Gene/Summary?db=core;g=AAEL012783) | protease m1 zinc metalloprotease [Source:VB External Description;Acc:AAEL012783] | -0.3467 | -0.63623 | -0.78512 | -0.9323 |
| [AAEL012808](https://www.vectorbase.org/aedes_aegypti/Gene/Summary?db=core;g=AAEL012808) | glucose dehydrogenase [Source:VB External Description;Acc:AAEL012808] | -0.02093 | -0.17421 | -0.41355 | -0.63399 |
| [AAEL012893](https://www.vectorbase.org/aedes_aegypti/Gene/Summary?db=core;g=AAEL012893) | microsomal dipeptidase [Source:VB External Description;Acc:AAEL012893] | -0.26635 | 0.508149 | -0.00046 | -1.24215 |
| [AAEL012903](https://www.vectorbase.org/aedes_aegypti/Gene/Summary?db=core;g=AAEL012903) | sugar transporter [Source:VB External Description;Acc:AAEL012903] | -0.20385 | 0.010205 | -0.50319 | -1.73455 |
| [AAEL012938](https://www.vectorbase.org/aedes_aegypti/Gene/Summary?db=core;g=AAEL012938) | zinc finger protein [Source:VB External Description;Acc:AAEL012938] | -0.22071 | -0.55727 | -0.57324 | -0.8087 |
| [AAEL012955](https://www.vectorbase.org/aedes_aegypti/Gene/Summary?db=core;g=AAEL012955) | phosphatidylethanolamine-binding protein [Source:VB External Description;Acc:AAEL012955] | -0.46154 | -0.77352 | -1.30449 | -1.11756 |
| [AAEL012958](https://www.vectorbase.org/aedes_aegypti/Gene/Summary?db=core;g=AAEL012958) |  | -0.93261 | -1.38545 | -0.90724 | -1.57286 |
| [AAEL013083](https://www.vectorbase.org/aedes_aegypti/Gene/Summary?db=core;g=AAEL013083) |  | -0.1468 | -0.3078 | -0.31066 | -0.90966 |
| [AAEL013132](https://www.vectorbase.org/aedes_aegypti/Gene/Summary?db=core;g=AAEL013132) | 6-phosphofructo-2-kinase/fructose-2,6-bisphosphatase short form [Source:VB External Description;Acc:AAEL013132] | -0.17474 | -0.12868 | -0.54513 | -0.75382 |
| [AAEL013214](https://www.vectorbase.org/aedes_aegypti/Gene/Summary?db=core;g=AAEL013214) | cgmp-dependent protein kinase [Source:VB External Description;Acc:AAEL013214] | 0.019488 | -0.08728 | -0.01467 | -0.56954 |
| [AAEL013263](https://www.vectorbase.org/aedes_aegypti/Gene/Summary?db=core;g=AAEL013263) | high affinity copper transporter, putative [Source:VB External Description;Acc:AAEL013263] | -0.3833 | -0.74058 | -0.87211 | -0.87265 |
| [AAEL013316](https://www.vectorbase.org/aedes_aegypti/Gene/Summary?db=core;g=AAEL013316) | smile protein [Source:VB External Description;Acc:AAEL013316] | 0.016204 | -0.20248 | -0.24687 | -0.74846 |
| [AAEL013356](https://www.vectorbase.org/aedes_aegypti/Gene/Summary?db=core;g=AAEL013356) |  | 0.109677 | 0.00201 | -0.04289 | -0.69783 |
| [AAEL013407](https://www.vectorbase.org/aedes_aegypti/Gene/Summary?db=core;g=AAEL013407) | catalase [Source:VB Community Annotation;Acc:AAEL013407] | -0.13334 | 0.109396 | -0.09512 | -0.5481 |
| [AAEL013429](https://www.vectorbase.org/aedes_aegypti/Gene/Summary?db=core;g=AAEL013429) |  | 0.025125 | -0.0263 | -0.19384 | -0.65455 |
| [AAEL013510](https://www.vectorbase.org/aedes_aegypti/Gene/Summary?db=core;g=AAEL013510) | smaug protein [Source:VB External Description;Acc:AAEL013510] | 0.041594 | 0.098283 | -0.26556 | -0.98195 |
| [AAEL013554](https://www.vectorbase.org/aedes_aegypti/Gene/Summary?db=core;g=AAEL013554) | cytochrome P450 (CYP4J14) [Source:VB Community Annotation;Acc:AAEL013554] | 0.002622 | -0.1764 | -0.45801 | -0.91633 |
| [AAEL013687](https://www.vectorbase.org/aedes_aegypti/Gene/Summary?db=core;g=AAEL013687) |  | -0.27145 | -0.98616 | 0.016368 | -1.15111 |
| [AAEL013741](https://www.vectorbase.org/aedes_aegypti/Gene/Summary?db=core;g=AAEL013741) |  | -0.11652 | -0.63237 | -0.54385 | -1.09017 |
| [AAEL013756](https://www.vectorbase.org/aedes_aegypti/Gene/Summary?db=core;g=AAEL013756) | zinc/iron transporter [Source:VB External Description;Acc:AAEL013756] | -0.21512 | -0.49577 | -0.38011 | -0.70888 |
| [AAEL013760](https://www.vectorbase.org/aedes_aegypti/Gene/Summary?db=core;g=AAEL013760) | homeobox protein nk-2 [Source:VB External Description;Acc:AAEL013760] | -0.55106 | -0.77924 | -0.65363 | -1.75197 |
| [AAEL013783](https://www.vectorbase.org/aedes_aegypti/Gene/Summary?db=core;g=AAEL013783) | protein farnesyltransferase alpha subunit [Source:VB External Description;Acc:AAEL013783] | -0.32852 | -0.28587 | -0.10801 | -0.54874 |
| [AAEL013784](https://www.vectorbase.org/aedes_aegypti/Gene/Summary?db=core;g=AAEL013784) |  | 0.025225 | -0.25405 | -0.06623 | -0.85188 |
| [AAEL013785](https://www.vectorbase.org/aedes_aegypti/Gene/Summary?db=core;g=AAEL013785) |  | -0.50012 | -0.50205 | -0.26656 | -0.68781 |
| [AAEL013843](https://www.vectorbase.org/aedes_aegypti/Gene/Summary?db=core;g=AAEL013843) |  | 0.007145 | -0.02929 | -0.31145 | -0.66323 |
| [AAEL013883](https://www.vectorbase.org/aedes_aegypti/Gene/Summary?db=core;g=AAEL013883) |  | -0.23127 | -0.34809 | -0.50154 | -0.65394 |
| [AAEL013895](https://www.vectorbase.org/aedes_aegypti/Gene/Summary?db=core;g=AAEL013895) |  | -0.43687 | -0.33773 | -2.23741 | -0.81224 |
| [AAEL013905](https://www.vectorbase.org/aedes_aegypti/Gene/Summary?db=core;g=AAEL013905) |  | -0.12037 | -0.24638 | -0.49876 | -0.83095 |
| [AAEL013913](https://www.vectorbase.org/aedes_aegypti/Gene/Summary?db=core;g=AAEL013913) | stretch regulated skeletal muscle protein, putative [Source:VB External Description;Acc:AAEL013913] | -0.51243 | -0.39657 | -0.65501 | -0.84438 |
| [AAEL013966](https://www.vectorbase.org/aedes_aegypti/Gene/Summary?db=core;g=AAEL013966) | ATP-dependent clp protease ATP-binding subunit clpx [Source:VB External Description;Acc:AAEL013966] | -0.16259 | -0.23548 | -0.44866 | -0.62445 |
| [AAEL014045](https://www.vectorbase.org/aedes_aegypti/Gene/Summary?db=core;g=AAEL014045) | allantoicase [Source:VB External Description;Acc:AAEL014045] | -0.04566 | -0.41792 | -0.22211 | -0.78932 |
| [AAEL014098](https://www.vectorbase.org/aedes_aegypti/Gene/Summary?db=core;g=AAEL014098) | polycomb protein [Source:VB External Description;Acc:AAEL014098] | 0.057846 | -0.21125 | -0.08536 | -0.76925 |
| [AAEL014110](https://www.vectorbase.org/aedes_aegypti/Gene/Summary?db=core;g=AAEL014110) | sulfite reductase [Source:VB External Description;Acc:AAEL014110] | -0.18248 | -0.22335 | -0.31435 | -0.69026 |
| [AAEL014141](https://www.vectorbase.org/aedes_aegypti/Gene/Summary?db=core;g=AAEL014141) | Serine Protease Inhibitor (serpin) likely cleavage at S/M. [Source:VB Community Annotation;Acc:AAEL014141] | 0.377812 | 0.175532 | -0.8348 | -1.00734 |
| [AAEL014199](https://www.vectorbase.org/aedes_aegypti/Gene/Summary?db=core;g=AAEL014199) | dihydropyrimidine dehydrogenase [Source:VB External Description;Acc:AAEL014199] | -0.03323 | -0.60564 | -0.50476 | -1.02001 |
| [AAEL014214](https://www.vectorbase.org/aedes_aegypti/Gene/Summary?db=core;g=AAEL014214) |  | -1.08268 | -0.92852 | -0.58635 | -1.14369 |
| [AAEL014248](https://www.vectorbase.org/aedes_aegypti/Gene/Summary?db=core;g=AAEL014248) |  | -0.29102 | -0.66957 | -0.94804 | -0.9126 |
| [AAEL014309](https://www.vectorbase.org/aedes_aegypti/Gene/Summary?db=core;g=AAEL014309) |  | -0.47694 | -0.67266 | -0.85046 | -0.54127 |
| [AAEL014371](https://www.vectorbase.org/aedes_aegypti/Gene/Summary?db=core;g=AAEL014371) | glucosyl/glucuronosyl transferases [Source:VB External Description;Acc:AAEL014371] | -0.19447 | -0.28734 | -0.63385 | -1.09572 |
| [AAEL014374](https://www.vectorbase.org/aedes_aegypti/Gene/Summary?db=core;g=AAEL014374) |  | -0.39903 | -1.91631 | -1.41951 | -2.48178 |
| [AAEL014382](https://www.vectorbase.org/aedes_aegypti/Gene/Summary?db=core;g=AAEL014382) | C-Type Lectin (CTL) - mannose binding. [Source:VB Community Annotation;Acc:AAEL014382] | -0.57374 | -1.15633 | -1.71259 | -1.30814 |
| [AAEL014406](https://www.vectorbase.org/aedes_aegypti/Gene/Summary?db=core;g=AAEL014406) | predicted protein [Source:VB External Description;Acc:AAEL014406] | -0.36796 | -0.75398 | -0.32169 | -1.02798 |
| [AAEL014411](https://www.vectorbase.org/aedes_aegypti/Gene/Summary?db=core;g=AAEL014411) | cytochrome P450 [Source:VB Community Annotation;Acc:AAEL014411] | -0.24697 | -0.37956 | -0.27582 | -0.64666 |
| [AAEL014441](https://www.vectorbase.org/aedes_aegypti/Gene/Summary?db=core;g=AAEL014441) |  | -0.11629 | -0.36 | -0.42642 | -0.87068 |
| [AAEL014493](https://www.vectorbase.org/aedes_aegypti/Gene/Summary?db=core;g=AAEL014493) | aldehyde oxidase [Source:VB External Description;Acc:AAEL014493] | 0.088394 | 0.051694 | -0.06123 | -0.72323 |
| [AAEL014514](https://www.vectorbase.org/aedes_aegypti/Gene/Summary?db=core;g=AAEL014514) | metalloproteinase, putative [Source:VB External Description;Acc:AAEL014514] | 0.170812 | -0.07397 | 0.034963 | -1.06805 |
| [AAEL014603](https://www.vectorbase.org/aedes_aegypti/Gene/Summary?db=core;g=AAEL014603) | cytochrome P450 [Source:VB Community Annotation;Acc:AAEL014603] | -0.23912 | -0.49341 | -0.81141 | -1.34746 |
| [AAEL014607](https://www.vectorbase.org/aedes_aegypti/Gene/Summary?db=core;g=AAEL014607) | cytochrome P450 [Source:VB Community Annotation;Acc:AAEL014607] | -0.13049 | -0.28265 | -0.26725 | -0.69608 |
| [AAEL014609](https://www.vectorbase.org/aedes_aegypti/Gene/Summary?db=core;g=AAEL014609) | cytochrome P450 [Source:VB Community Annotation;Acc:AAEL014609] | -0.40552 | -0.4648 | -0.68359 | -1.27131 |
| [AAEL014610](https://www.vectorbase.org/aedes_aegypti/Gene/Summary?db=core;g=AAEL014610) | cytochrome P450 [Source:VB Community Annotation;Acc:AAEL014610] | 0.022417 | -0.75112 | -0.43089 | -1.18897 |
| [AAEL014613](https://www.vectorbase.org/aedes_aegypti/Gene/Summary?db=core;g=AAEL014613) | cytochrome P450 (CYP9J24) [Source:VB Community Annotation;Acc:AAEL014613] | -0.45282 | -0.97951 | -1.33714 | -0.83183 |
| [AAEL014614](https://www.vectorbase.org/aedes_aegypti/Gene/Summary?db=core;g=AAEL014614) | cytochrome P450 [Source:VB External Description;Acc:AAEL014614] | -0.17027 | -0.08056 | -0.25357 | -0.70303 |
| [AAEL014616](https://www.vectorbase.org/aedes_aegypti/Gene/Summary?db=core;g=AAEL014616) | cytochrome P450 (CYP9J27) [Source:VB Community Annotation;Acc:AAEL014616] | -0.36374 | -0.35467 | -0.4463 | -0.85411 |
| [AAEL014619](https://www.vectorbase.org/aedes_aegypti/Gene/Summary?db=core;g=AAEL014619) | cytochrome P450 (CYP9J22) [Source:VB Community Annotation;Acc:AAEL014619] | -0.10449 | 0.248865 | -0.27537 | -0.79295 |
| [AAEL014664](https://www.vectorbase.org/aedes_aegypti/Gene/Summary?db=core;g=AAEL014664) | AMP dependent coa ligase [Source:VB External Description;Acc:AAEL014664] | -0.39028 | -0.82211 | -0.6974 | -1.54497 |
| [AAEL014697](https://www.vectorbase.org/aedes_aegypti/Gene/Summary?db=core;g=AAEL014697) |  | -0.12282 | -0.28031 | -0.34278 | -0.79017 |
| [AAEL014699](https://www.vectorbase.org/aedes_aegypti/Gene/Summary?db=core;g=AAEL014699) | ABC transporter [Source:VB External Description;Acc:AAEL014699] | -0.10719 | -0.64512 | -0.6154 | -0.82568 |
| [AAEL014703](https://www.vectorbase.org/aedes_aegypti/Gene/Summary?db=core;g=AAEL014703) |  | -0.05918 | -0.67932 | -0.39221 | -0.60319 |
| [AAEL014733](https://www.vectorbase.org/aedes_aegypti/Gene/Summary?db=core;g=AAEL014733) | nuclear pore complex protein nup214 [Source:VB External Description;Acc:AAEL014733] | -0.02109 | -0.53876 | -0.67551 | -0.70833 |
| [AAEL014746](https://www.vectorbase.org/aedes_aegypti/Gene/Summary?db=core;g=AAEL014746) | o-linked n-acetylglucosamine transferase, ogt [Source:VB External Description;Acc:AAEL014746] | -0.05355 | -0.01518 | -0.35804 | -0.66592 |
| [AAEL014768](https://www.vectorbase.org/aedes_aegypti/Gene/Summary?db=core;g=AAEL014768) | glutamate synthase [Source:VB External Description;Acc:AAEL014768] | 0.14344 | -0.32412 | -0.59771 | -0.74595 |
| [AAEL014815](https://www.vectorbase.org/aedes_aegypti/Gene/Summary?db=core;g=AAEL014815) | vacuolar protein sorting-associated protein (vps13) [Source:VB External Description;Acc:AAEL014815] | -0.08195 | -0.31344 | 0.030159 | -0.80239 |
| [AAEL014842](https://www.vectorbase.org/aedes_aegypti/Gene/Summary?db=core;g=AAEL014842) | multiple inositol polyphosphate phosphatase [Source:VB External Description;Acc:AAEL014842] | -0.30452 | -0.79803 | -0.93571 | -1.47323 |
| [AAEL014890](https://www.vectorbase.org/aedes_aegypti/Gene/Summary?db=core;g=AAEL014890) | cytochrome P450 [Source:VB Community Annotation;Acc:AAEL014890] | -0.18496 | -0.37582 | -0.40591 | -0.69012 |
| [AAEL014891](https://www.vectorbase.org/aedes_aegypti/Gene/Summary?db=core;g=AAEL014891) | cytochrome P450 [Source:VB Community Annotation;Acc:AAEL014891] | -0.43774 | -0.57411 | -0.97725 | -1.395 |
| [AAEL014892](https://www.vectorbase.org/aedes_aegypti/Gene/Summary?db=core;g=AAEL014892) | cytochrome P450 [Source:VB External Description;Acc:AAEL014892] | -0.29355 | -0.20367 | -0.58842 | -0.75034 |
| [AAEL014893](https://www.vectorbase.org/aedes_aegypti/Gene/Summary?db=core;g=AAEL014893) | cytochrome P450 [Source:VB Community Annotation;Acc:AAEL014893] | -0.25142 | -0.19483 | -0.6186 | -1.59694 |
| [AAEL015014](https://www.vectorbase.org/aedes_aegypti/Gene/Summary?db=core;g=AAEL015014) |  | -0.1506 | -0.3419 | -0.00899 | -0.92205 |
| [AAEL015036](https://www.vectorbase.org/aedes_aegypti/Gene/Summary?db=core;g=AAEL015036) | protease S51 alpha-aspartyl dipeptidase [Source:VB External Description;Acc:AAEL015036] | -1.06319 | -0.77389 | -0.63522 | -0.92921 |
| [AAEL015040](https://www.vectorbase.org/aedes_aegypti/Gene/Summary?db=core;g=AAEL015040) | multiple inositol polyphosphate phosphatase [Source:VB External Description;Acc:AAEL015040] | -0.27698 | -1.03303 | -1.19308 | -1.41605 |
| [AAEL015044](https://www.vectorbase.org/aedes_aegypti/Gene/Summary?db=core;g=AAEL015044) |  | -0.17201 | -0.81007 | -1.16386 | -1.33543 |
| [AAEL015083](https://www.vectorbase.org/aedes_aegypti/Gene/Summary?db=core;g=AAEL015083) |  | -0.25093 | -0.30082 | -0.1778 | -0.69547 |
| [AAEL015136](https://www.vectorbase.org/aedes_aegypti/Gene/Summary?db=core;g=AAEL015136) | Niemann-Pick Type C-2, putative [Source:VB External Description;Acc:AAEL015136] | -0.67364 | -1.48805 | -0.53072 | -1.30886 |
| [AAEL015172](https://www.vectorbase.org/aedes_aegypti/Gene/Summary?db=core;g=AAEL015172) |  | -0.15597 | -0.81858 | -0.88552 | -0.97189 |
| [AAEL015249](https://www.vectorbase.org/aedes_aegypti/Gene/Summary?db=core;g=AAEL015249) | sulphate transporter [Source:VB External Description;Acc:AAEL015249] | -0.15276 | -0.37746 | -0.2088 | -0.65201 |
| [AAEL015304](https://www.vectorbase.org/aedes_aegypti/Gene/Summary?db=core;g=AAEL015304) |  | -0.21035 | -0.2798 | -0.13487 | -0.7973 |
| [AAEL015315](https://www.vectorbase.org/aedes_aegypti/Gene/Summary?db=core;g=AAEL015315) | malate synthase [Source:VB External Description;Acc:AAEL015315] | 0.079264 | -0.29767 | -0.33464 | -1.09632 |
| [AAEL015330](https://www.vectorbase.org/aedes_aegypti/Gene/Summary?db=core;g=AAEL015330) |  | 0.009688 | -0.30903 | -0.37661 | -0.73179 |
| [AAEL015391](https://www.vectorbase.org/aedes_aegypti/Gene/Summary?db=core;g=AAEL015391) | gamma glutamyl transpeptidases [Source:VB External Description;Acc:AAEL015391] | -0.38891 | -0.52033 | -0.42552 | -1.79375 |
| [AAEL015435](https://www.vectorbase.org/aedes_aegypti/Gene/Summary?db=core;g=AAEL015435) |  | -0.08899 | -0.10921 | -0.41015 | -0.97501 |
| [AAEL015603](https://www.vectorbase.org/aedes_aegypti/Gene/Summary?db=core;g=AAEL015603) | ceramide kinase [Source:VB External Description;Acc:AAEL015603] | 0.075238 | -0.03047 | -0.29607 | -0.77486 |
| [AAEL015641](https://www.vectorbase.org/aedes_aegypti/Gene/Summary?db=core;g=AAEL015641) | cytochrome P450 [Source:VB External Description;Acc:AAEL015641] | -0.28975 | -0.39034 | -0.26867 | -0.80934 |
| [AAEL017061](https://www.vectorbase.org/aedes_aegypti/Gene/Summary?db=core;g=AAEL017061) |  | -0.2563 | -0.43414 | -0.71166 | -0.90985 |
| [AAEL017229](https://www.vectorbase.org/aedes_aegypti/Gene/Summary?db=core;g=AAEL017229) |  | -0.23823 | -0.57384 | -0.53403 | -1.03668 |
| [AAEL017365](https://www.vectorbase.org/aedes_aegypti/Gene/Summary?db=core;g=AAEL017365) |  | 0.08675 | -0.58966 | -0.62936 | -0.68098 |
| [AAEL017440](https://www.vectorbase.org/aedes_aegypti/Gene/Summary?db=core;g=AAEL017440) |  | -0.03367 | -0.08795 | -0.50804 | -0.96705 |
| [AAEL017512](https://www.vectorbase.org/aedes_aegypti/Gene/Summary?db=core;g=AAEL017512) |  | -0.1983 | -0.18656 | -0.39789 | -0.6722 |
| [AAEL017523](https://www.vectorbase.org/aedes_aegypti/Gene/Summary?db=core;g=AAEL017523) | Toll-like receptor [Source:VB Community Annotation;Acc:AAEL017523] | -0.59305 | -0.88107 | -1.07007 | -1.09873 |
| [AAEL017539](https://www.vectorbase.org/aedes_aegypti/Gene/Summary?db=core;g=AAEL017539) | cytochrome P450 [Source:VB Community Annotation;Acc:AAEL017539] | 0.396703 | 0.126217 | 0.406618 | -0.76293 |
| [AAEL018037](https://www.vectorbase.org/aedes_aegypti/Gene/Summary?db=core;g=AAEL018037) |  | -0.22629 | -0.54283 | -0.71896 | -0.82105 |
| [AAEL018096](https://www.vectorbase.org/aedes_aegypti/Gene/Summary?db=core;g=AAEL018096) |  | -0.03978 | -0.1743 | -0.43934 | -0.64823 |
| [AAEL018178](https://www.vectorbase.org/aedes_aegypti/Gene/Summary?db=core;g=AAEL018178) |  | -0.11713 | -0.18273 | -0.52509 | -0.61481 |
| [AAEL018197](https://www.vectorbase.org/aedes_aegypti/Gene/Summary?db=core;g=AAEL018197) |  | -1.91341 | -0.56358 | -1.00856 | -3.49041 |
| [AAEL018295](https://www.vectorbase.org/aedes_aegypti/Gene/Summary?db=core;g=AAEL018295) |  | -0.16752 | -0.3233 | -0.07624 | -0.90813 |
| [AAEL018331](https://www.vectorbase.org/aedes_aegypti/Gene/Summary?db=core;g=AAEL018331) |  | -0.18839 | -0.37019 | -0.46883 | -0.70209 |
| [AAEL018345](https://www.vectorbase.org/aedes_aegypti/Gene/Summary?db=core;g=AAEL018345) |  | -0.53006 | -0.7223 | -0.55569 | -0.51351 |
| Down Regulated, Strong (clusterB) |  |  |  |  |  |
| [AAEL000777](https://www.vectorbase.org/aedes_aegypti/Gene/Summary?db=core;g=AAEL000777) | cecropin anti-microbial peptide [Source:VB Community Annotation;Acc:AAEL000777] | -3.74385 | -0.86441 | -7.07376 | -3.37919 |
| [AAEL003816](https://www.vectorbase.org/aedes_aegypti/Gene/Summary?db=core;g=AAEL003816) |  | -3.39492 | -1.61209 | -4.84423 | -3.49706 |
| [AAEL003841](https://www.vectorbase.org/aedes_aegypti/Gene/Summary?db=core;g=AAEL003841) | defensin anti-microbial peptide [Source:VB Community Annotation;Acc:AAEL003841] | -4.00806 | -2.64505 | -5.13315 | -2.58607 |
| [AAEL003843](https://www.vectorbase.org/aedes_aegypti/Gene/Summary?db=core;g=AAEL003843) |  | -1.89244 | -0.5836 | -2.68445 | -1.25618 |
| [AAEL003857](https://www.vectorbase.org/aedes_aegypti/Gene/Summary?db=core;g=AAEL003857) | defensin anti-microbial peptide [Source:VB Community Annotation;Acc:AAEL003857] | -2.84725 | -1.98896 | -3.80304 | -0.70731 |
| [AAEL004522](https://www.vectorbase.org/aedes_aegypti/Gene/Summary?db=core;g=AAEL004522) | gambicin anti-microbial peptide [Source:VB Community Annotation;Acc:AAEL004522] | -0.98378 | -0.92832 | -4.10854 | -2.20741 |
| [AAEL009888](https://www.vectorbase.org/aedes_aegypti/Gene/Summary?db=core;g=AAEL009888) | sodium-coupled cation-chloride cotransporter [Source:VB Community Annotation;Acc:AAEL009888] | -3.325 | -6.26475 | -6.16273 | -0.51778 |
| [AAEL010338](https://www.vectorbase.org/aedes_aegypti/Gene/Summary?db=core;g=AAEL010338) |  | -7.95125 | -2.5873 | -7.93606 | -5.81268 |
| [AAEL012852](https://www.vectorbase.org/aedes_aegypti/Gene/Summary?db=core;g=AAEL012852) | trypsin [Source:VB External Description;Acc:AAEL012852] | -5.68767 | -3.87477 | -3.56562 | -2.01283 |
| [AAEL017052](https://www.vectorbase.org/aedes_aegypti/Gene/Summary?db=core;g=AAEL017052) |  | -1.39629 | -1.60582 | -3.05878 | -1.19219 |
| Up regulated  (cluster C) |  |  |  |  |  |
| [AAEL000050](https://www.vectorbase.org/aedes_aegypti/Gene/Summary?db=core;g=AAEL000050) |  | -0.06729 | 0.224397 | 0.031387 | 0.652614 |
| [AAEL000080](https://www.vectorbase.org/aedes_aegypti/Gene/Summary?db=core;g=AAEL000080) | phosphoenolpyruvate carboxykinase [Source:VB External Description;Acc:AAEL000080] | 0.161159 | 0.572142 | 1.088328 | 1.686991 |
| [AAEL000184](https://www.vectorbase.org/aedes_aegypti/Gene/Summary?db=core;g=AAEL000184) |  | 0.324333 | 0.795301 | 0.689656 | 1.215566 |
| [AAEL000187](https://www.vectorbase.org/aedes_aegypti/Gene/Summary?db=core;g=AAEL000187) | lim-kinase1 [Source:VB External Description;Acc:AAEL000187] | 0.380827 | 0.70239 | 1.266431 | 1.568503 |
| [AAEL000199](https://www.vectorbase.org/aedes_aegypti/Gene/Summary?db=core;g=AAEL000199) | arginyl-tRNA synthetase [Source:VB External Description;Acc:AAEL000199] | 0.119373 | 0.141563 | 0.274888 | 0.659558 |
| [AAEL000214](https://www.vectorbase.org/aedes_aegypti/Gene/Summary?db=core;g=AAEL000214) | vacuolar protein sorting (vps33) [Source:VB External Description;Acc:AAEL000214] | 0.168592 | 0.478626 | 0.554897 | 1.020516 |
| [AAEL000321](https://www.vectorbase.org/aedes_aegypti/Gene/Summary?db=core;g=AAEL000321) | acetyl-coa synthetase [Source:VB External Description;Acc:AAEL000321] | -0.05396 | 0.203885 | 0.57803 | 1.071741 |
| [AAEL000339](https://www.vectorbase.org/aedes_aegypti/Gene/Summary?db=core;g=AAEL000339) | lim domain [Source:VB External Description;Acc:AAEL000339] | 0.070444 | 0.386423 | 0.427924 | 0.890165 |
| [AAEL000345](https://www.vectorbase.org/aedes_aegypti/Gene/Summary?db=core;g=AAEL000345) | membrane associated guanylate kinase inverted 1, magi1 [Source:VB External Description;Acc:AAEL000345] | 0.080715 | 0.43181 | 0.743912 | 1.087892 |
| [AAEL000378](https://www.vectorbase.org/aedes_aegypti/Gene/Summary?db=core;g=AAEL000378) | integrin-linked protein kinase 2 (ilk-2) [Source:VB External Description;Acc:AAEL000378] | -0.02119 | 0.204759 | 0.351313 | 0.748297 |
| [AAEL000399](https://www.vectorbase.org/aedes_aegypti/Gene/Summary?db=core;g=AAEL000399) |  | -0.05466 | 0.067294 | 0.309614 | 0.670354 |
| [AAEL000447](https://www.vectorbase.org/aedes_aegypti/Gene/Summary?db=core;g=AAEL000447) | V-1 protein, putative [Source:VB External Description;Acc:AAEL000447] | 0.246689 | 0.605997 | 0.514553 | 1.080589 |
| [AAEL000450](https://www.vectorbase.org/aedes_aegypti/Gene/Summary?db=core;g=AAEL000450) | ras GTPase activating protein [Source:VB External Description;Acc:AAEL000450] | 0.309145 | 0.647518 | 0.627543 | 0.961709 |
| [AAEL000488](https://www.vectorbase.org/aedes_aegypti/Gene/Summary?db=core;g=AAEL000488) |  | 0.233143 | 0.819321 | 1.719785 | 2.978892 |
| [AAEL000489](https://www.vectorbase.org/aedes_aegypti/Gene/Summary?db=core;g=AAEL000489) | P21-activated kinase, pak [Source:VB External Description;Acc:AAEL000489] | 0.079616 | 0.574201 | 0.580855 | 0.998268 |
| [AAEL000495](https://www.vectorbase.org/aedes_aegypti/Gene/Summary?db=core;g=AAEL000495) | glutathione peroxidase [Source:VB Community Annotation;Acc:AAEL000495] | 1.415331 | 1.51317 | 0.773365 | 1.746824 |
| [AAEL000505](https://www.vectorbase.org/aedes_aegypti/Gene/Summary?db=core;g=AAEL000505) |  | 0.075621 | 0.464861 | 0.612689 | 1.310515 |
| [AAEL000541](https://www.vectorbase.org/aedes_aegypti/Gene/Summary?db=core;g=AAEL000541) | fasciclin, putative [Source:VB External Description;Acc:AAEL000541] | -0.00855 | 0.280174 | 0.409745 | 1.03254 |
| [AAEL000551](https://www.vectorbase.org/aedes_aegypti/Gene/Summary?db=core;g=AAEL000551) |  | 0.26488 | -0.37229 | 0.200726 | 1.255846 |
| [AAEL000599](https://www.vectorbase.org/aedes_aegypti/Gene/Summary?db=core;g=AAEL000599) | wingless protein, putative [Source:VB External Description;Acc:AAEL000599] | 0.574253 | 0.75444 | 1.091013 | 0.972723 |
| [AAEL000605](https://www.vectorbase.org/aedes_aegypti/Gene/Summary?db=core;g=AAEL000605) | rab geranylgeranyl transferase alpha subunit [Source:VB External Description;Acc:AAEL000605] | -0.01896 | 0.386752 | 0.449602 | 0.908897 |
| [AAEL000611](https://www.vectorbase.org/aedes_aegypti/Gene/Summary?db=core;g=AAEL000611) | cecropin anti-microbial peptide [Source:VB Community Annotation;Acc:AAEL000611] | -0.29939 | 0.136686 | 0.180492 | 3.774946 |
| [AAEL000621](https://www.vectorbase.org/aedes_aegypti/Gene/Summary?db=core;g=AAEL000621) | cecropin anti-microbial peptide [Source:VB Community Annotation;Acc:AAEL000621] | -1.04176 | 0.024584 | 0.386132 | 4.087775 |
| [AAEL000634](https://www.vectorbase.org/aedes_aegypti/Gene/Summary?db=core;g=AAEL000634) | ceramide glucosyltransferase [Source:VB External Description;Acc:AAEL000634] | 0.252954 | 0.401217 | 0.802902 | 1.107822 |
| [AAEL000706](https://www.vectorbase.org/aedes_aegypti/Gene/Summary?db=core;g=AAEL000706) | serine palmitoyltransferase [Source:VB External Description;Acc:AAEL000706] | 0.043402 | 0.233497 | 0.581461 | 0.719511 |
| [AAEL000716](https://www.vectorbase.org/aedes_aegypti/Gene/Summary?db=core;g=AAEL000716) | chondroitin 4-sulfotransferase [Source:VB External Description;Acc:AAEL000716] | 0.167094 | 0.368029 | 0.624016 | 0.835462 |
| [AAEL000759](https://www.vectorbase.org/aedes_aegypti/Gene/Summary?db=core;g=AAEL000759) | gamma-glutamylcysteine synthetase, putative [Source:VB External Description;Acc:AAEL000759] | 0.138323 | 0.712308 | 0.397049 | 1.26984 |
| [AAEL000805](https://www.vectorbase.org/aedes_aegypti/Gene/Summary?db=core;g=AAEL000805) |  | -0.20034 | 0.364495 | 1.07695 | 1.337517 |
| [AAEL000807](https://www.vectorbase.org/aedes_aegypti/Gene/Summary?db=core;g=AAEL000807) | tetratricopeptide repeat protein, putative [Source:VB External Description;Acc:AAEL000807] | 0.109791 | 0.718501 | 0.616254 | 1.079092 |
| [AAEL000811](https://www.vectorbase.org/aedes_aegypti/Gene/Summary?db=core;g=AAEL000811) | GPCR Methuselah Family [Source:VB Community Annotation;Acc:AAEL000811] | 0.342844 | 0.63199 | 0.84327 | 1.502524 |
| [AAEL000813](https://www.vectorbase.org/aedes_aegypti/Gene/Summary?db=core;g=AAEL000813) | dimethylaniline monooxygenase [Source:VB External Description;Acc:AAEL000813] | 0.312569 | 0.7459 | 0.786744 | 1.546636 |
| [AAEL000819](https://www.vectorbase.org/aedes_aegypti/Gene/Summary?db=core;g=AAEL000819) |  | 0.411583 | 0.652381 | 0.790076 | 1.271151 |
| [AAEL000859](https://www.vectorbase.org/aedes_aegypti/Gene/Summary?db=core;g=AAEL000859) |  | -0.2301 | 0.188824 | 0.12708 | 1.339943 |
| [AAEL000898](https://www.vectorbase.org/aedes_aegypti/Gene/Summary?db=core;g=AAEL000898) |  | -0.08297 | 0.338283 | 0.617998 | 1.458473 |
| [AAEL000905](https://www.vectorbase.org/aedes_aegypti/Gene/Summary?db=core;g=AAEL000905) |  | -0.21889 | -0.05585 | 0.111695 | 1.460245 |
| [AAEL000934](https://www.vectorbase.org/aedes_aegypti/Gene/Summary?db=core;g=AAEL000934) | clathrin light chain [Source:VB External Description;Acc:AAEL000934] | 0.027591 | 0.399045 | 0.294919 | 0.818973 |
| [AAEL000999](https://www.vectorbase.org/aedes_aegypti/Gene/Summary?db=core;g=AAEL000999) | DNA replication licensing factor MCM7 [Source:VB External Description;Acc:AAEL000999] | 0.074756 | 0.310762 | -0.16973 | 0.83749 |
| [AAEL001005](https://www.vectorbase.org/aedes_aegypti/Gene/Summary?db=core;g=AAEL001005) | calreticulin [Source:VB External Description;Acc:AAEL001005] | 0.13874 | 0.852584 | 0.552556 | 1.156295 |
| [AAEL001011](https://www.vectorbase.org/aedes_aegypti/Gene/Summary?db=core;g=AAEL001011) |  | 0.511787 | 0.694455 | 0.602553 | 1.072022 |
| [AAEL001014](https://www.vectorbase.org/aedes_aegypti/Gene/Summary?db=core;g=AAEL001014) | vacuolar protein sorting-associated [Source:VB External Description;Acc:AAEL001014] | -0.0053 | 0.171156 | 0.302888 | 0.815339 |
| [AAEL001048](https://www.vectorbase.org/aedes_aegypti/Gene/Summary?db=core;g=AAEL001048) | short-chain dehydrogenase [Source:VB External Description;Acc:AAEL001048] | -0.07801 | 0.090365 | 0.201821 | 0.959186 |
| [AAEL001052](https://www.vectorbase.org/aedes_aegypti/Gene/Summary?db=core;g=AAEL001052) | heat shock protein, putative [Source:VB External Description;Acc:AAEL001052] | 0.22793 | 0.755252 | 0.389333 | 0.860404 |
| [AAEL001054](https://www.vectorbase.org/aedes_aegypti/Gene/Summary?db=core;g=AAEL001054) | glutathione S-transferase (GSTD4) [Source:VB Community Annotation;Acc:AAEL001054] | -0.10082 | 0.657207 | 0.880939 | 2.014055 |
| [AAEL001084](https://www.vectorbase.org/aedes_aegypti/Gene/Summary?db=core;g=AAEL001084) | Clip-Domain Serine Protease family B. Protease homologue. [Source:VB Community Annotation;Acc:AAEL001084] | -0.18461 | -0.71071 | 0.651405 | 1.270171 |
| [AAEL001089](https://www.vectorbase.org/aedes_aegypti/Gene/Summary?db=core;g=AAEL001089) |  | 0.250986 | 0.45482 | 0.450549 | 0.732686 |
| [AAEL001090](https://www.vectorbase.org/aedes_aegypti/Gene/Summary?db=core;g=AAEL001090) | glutathione S-transferase (GSTD7) [Source:VB Community Annotation;Acc:AAEL001090] | 0.593051 | 0.962554 | 1.924608 | 2.297277 |
| [AAEL001091](https://www.vectorbase.org/aedes_aegypti/Gene/Summary?db=core;g=AAEL001091) | malic enzyme [Source:VB External Description;Acc:AAEL001091] | 0.392489 | 0.726523 | 0.927176 | 1.10478 |
| [AAEL001094](https://www.vectorbase.org/aedes_aegypti/Gene/Summary?db=core;g=AAEL001094) |  | 0.305329 | 0.574177 | 0.573546 | 0.78611 |
| [AAEL001130](https://www.vectorbase.org/aedes_aegypti/Gene/Summary?db=core;g=AAEL001130) | alpha-amylase [Source:VB External Description;Acc:AAEL001130] | 0.404268 | 0.35926 | 0.60246 | 1.056258 |
| [AAEL001142](https://www.vectorbase.org/aedes_aegypti/Gene/Summary?db=core;g=AAEL001142) | rab gdp/GTP exchange factor [Source:VB External Description;Acc:AAEL001142] | 0.207581 | 0.386989 | 0.500235 | 0.806341 |
| [AAEL001166](https://www.vectorbase.org/aedes_aegypti/Gene/Summary?db=core;g=AAEL001166) | protein phosphatases pp1 regulatory subunit [Source:VB External Description;Acc:AAEL001166] | 0.05705 | 0.275459 | 0.282698 | 0.840376 |
| [AAEL001212](https://www.vectorbase.org/aedes_aegypti/Gene/Summary?db=core;g=AAEL001212) | phosphoinositide-binding protein, putative [Source:VB External Description;Acc:AAEL001212] | 0.124688 | 0.36144 | 0.31247 | 0.782495 |
| [AAEL001223](https://www.vectorbase.org/aedes_aegypti/Gene/Summary?db=core;g=AAEL001223) | engulfment and cell motility protein [Source:VB External Description;Acc:AAEL001223] | 0.10796 | 0.649125 | 0.699225 | 0.967695 |
| [AAEL001232](https://www.vectorbase.org/aedes_aegypti/Gene/Summary?db=core;g=AAEL001232) | tubulointerstitial nephritis antigen [Source:VB External Description;Acc:AAEL001232] | -0.04188 | 0.477245 | 1.258981 | 2.281512 |
| [AAEL001264](https://www.vectorbase.org/aedes_aegypti/Gene/Summary?db=core;g=AAEL001264) |  | 0.091519 | 0.328296 | 0.706746 | 1.101122 |
| [AAEL001307](https://www.vectorbase.org/aedes_aegypti/Gene/Summary?db=core;g=AAEL001307) | SEC14, putative [Source:VB External Description;Acc:AAEL001307] | 0.069028 | 0.012972 | 0.310691 | 0.701791 |
| [AAEL001308](https://www.vectorbase.org/aedes_aegypti/Gene/Summary?db=core;g=AAEL001308) | CRAL/TRIO domain-containing protein [Source:VB External Description;Acc:AAEL001308] | 0.02505 | 0.357872 | 0.910807 | 1.52032 |
| [AAEL001318](https://www.vectorbase.org/aedes_aegypti/Gene/Summary?db=core;g=AAEL001318) | CRAL/TRIO domain-containing protein [Source:VB External Description;Acc:AAEL001318] | 0.587087 | 1.32761 | 1.990739 | 2.476418 |
| [AAEL001331](https://www.vectorbase.org/aedes_aegypti/Gene/Summary?db=core;g=AAEL001331) | mannose-1-phosphate guanyltransferase [Source:VB External Description;Acc:AAEL001331] | -0.20309 | 0.10921 | 0.515251 | 1.226207 |
| [AAEL001336](https://www.vectorbase.org/aedes_aegypti/Gene/Summary?db=core;g=AAEL001336) | charged multivesicular body protein 2a [Source:VB External Description;Acc:AAEL001336] | 0.010759 | 0.23422 | 0.22727 | 0.836734 |
| [AAEL001356](https://www.vectorbase.org/aedes_aegypti/Gene/Summary?db=core;g=AAEL001356) | RNA-binding protein [Source:VB External Description;Acc:AAEL001356] | -0.22891 | 0.043104 | 0.425557 | 0.783867 |
| [AAEL001385](https://www.vectorbase.org/aedes_aegypti/Gene/Summary?db=core;g=AAEL001385) |  | 0.042664 | 0.197741 | 0.268202 | 0.71525 |
| [AAEL001411](https://www.vectorbase.org/aedes_aegypti/Gene/Summary?db=core;g=AAEL001411) | myosin heavy chain, nonmuscle or smooth muscle [Source:VB External Description;Acc:AAEL001411] | 0.009414 | 0.348833 | 0.420554 | 1.025099 |
| [AAEL001471](https://www.vectorbase.org/aedes_aegypti/Gene/Summary?db=core;g=AAEL001471) |  | 0.120824 | 0.126191 | 0.353782 | 0.911337 |
| [AAEL001473](https://www.vectorbase.org/aedes_aegypti/Gene/Summary?db=core;g=AAEL001473) | dynamin-associated protein [Source:VB External Description;Acc:AAEL001473] | -0.07154 | 0.270558 | 0.295181 | 0.678649 |
| [AAEL001511](https://www.vectorbase.org/aedes_aegypti/Gene/Summary?db=core;g=AAEL001511) |  | 0.341063 | 0.415633 | 1.516944 | 3.337406 |
| [AAEL001543](https://www.vectorbase.org/aedes_aegypti/Gene/Summary?db=core;g=AAEL001543) |  | 0.04057 | 0.915116 | 0.926841 | 0.938879 |
| [AAEL001565](https://www.vectorbase.org/aedes_aegypti/Gene/Summary?db=core;g=AAEL001565) | peptidyl-glycine alpha-amidating monooxygenase [Source:VB External Description;Acc:AAEL001565] | 0.057577 | 0.189197 | 0.115817 | 0.70804 |
| [AAEL001576](https://www.vectorbase.org/aedes_aegypti/Gene/Summary?db=core;g=AAEL001576) |  | 0.324002 | 0.815488 | 0.809175 | 1.272601 |
| [AAEL001604](https://www.vectorbase.org/aedes_aegypti/Gene/Summary?db=core;g=AAEL001604) | guanine nucleotide exchange factor [Source:VB External Description;Acc:AAEL001604] | 0.586309 | 0.848817 | 1.100428 | 1.436816 |
| [AAEL001606](https://www.vectorbase.org/aedes_aegypti/Gene/Summary?db=core;g=AAEL001606) | GPCR Melatonin Family [Source:VB Community Annotation;Acc:AAEL001606] | 0.038467 | 0.080539 | 0.544157 | 1.306127 |
| [AAEL001616](https://www.vectorbase.org/aedes_aegypti/Gene/Summary?db=core;g=AAEL001616) | vesicular-fusion protein nsf [Source:VB External Description;Acc:AAEL001616] | 0.262276 | 0.490456 | 0.858089 | 0.982346 |
| [AAEL001659](https://www.vectorbase.org/aedes_aegypti/Gene/Summary?db=core;g=AAEL001659) | misexpression suppressor of ras, putative [Source:VB External Description;Acc:AAEL001659] | 0.370076 | 0.590345 | 0.735909 | 1.457274 |
| [AAEL001698](https://www.vectorbase.org/aedes_aegypti/Gene/Summary?db=core;g=AAEL001698) | charged multivesicular body protein 4b [Source:VB External Description;Acc:AAEL001698] | 0.104604 | 0.315409 | 0.325523 | 0.7542 |
| [AAEL001731](https://www.vectorbase.org/aedes_aegypti/Gene/Summary?db=core;g=AAEL001731) | Dual specificity tyrosine-phosphorylation-regulated kinase [Source:VB External Description;Acc:AAEL001731] | 0.127369 | 1.096575 | 1.668855 | 1.795929 |
| [AAEL001749](https://www.vectorbase.org/aedes_aegypti/Gene/Summary?db=core;g=AAEL001749) | ventrhoid transmembrane protein, putative [Source:VB External Description;Acc:AAEL001749] | 0.629399 | 0.335785 | 1.123324 | 2.066469 |
| [AAEL001901](https://www.vectorbase.org/aedes_aegypti/Gene/Summary?db=core;g=AAEL001901) | MRAS2, putative [Source:VB External Description;Acc:AAEL001901] | 0.799931 | 0.713112 | 0.532596 | 2.094266 |
| [AAEL001914](https://www.vectorbase.org/aedes_aegypti/Gene/Summary?db=core;g=AAEL001914) | scavenger receptor, putative [Source:VB External Description;Acc:AAEL001914] | 0.158813 | 0.487717 | 0.618707 | 0.856214 |
| [AAEL001919](https://www.vectorbase.org/aedes_aegypti/Gene/Summary?db=core;g=AAEL001919) | protein tyrosine phosphatase, non-receptor type nt1 [Source:VB External Description;Acc:AAEL001919] | 0.083243 | 0.367463 | 0.581926 | 0.910359 |
| [AAEL001930](https://www.vectorbase.org/aedes_aegypti/Gene/Summary?db=core;g=AAEL001930) | pra1 protein [Source:VB External Description;Acc:AAEL001930] | -0.02424 | 0.079269 | 0.209276 | 0.970771 |
| [AAEL001935](https://www.vectorbase.org/aedes_aegypti/Gene/Summary?db=core;g=AAEL001935) | CTL-like protein 1 [Source:VB External Description;Acc:AAEL001935] | 0.121304 | 0.596424 | 0.5191 | 1.199743 |
| [AAEL002092](https://www.vectorbase.org/aedes_aegypti/Gene/Summary?db=core;g=AAEL002092) | cuticle protein, putative [Source:VB External Description;Acc:AAEL002092] | -0.63946 | -0.44263 | -1.04759 | 2.129781 |
| [AAEL002102](https://www.vectorbase.org/aedes_aegypti/Gene/Summary?db=core;g=AAEL002102) |  | -0.01557 | 0.492812 | 0.38023 | 0.854927 |
| [AAEL002108](https://www.vectorbase.org/aedes_aegypti/Gene/Summary?db=core;g=AAEL002108) | nucleolar essential protein 1 (nep1) [Source:VB External Description;Acc:AAEL002108] | -0.00925 | 0.051008 | 0.33067 | 1.19106 |
| [AAEL002109](https://www.vectorbase.org/aedes_aegypti/Gene/Summary?db=core;g=AAEL002109) |  | 0.37598 | -0.01844 | 0.811414 | 2.308533 |
| [AAEL002130](https://www.vectorbase.org/aedes_aegypti/Gene/Summary?db=core;g=AAEL002130) | ecdysone inducible protein L2, putative [Source:VB External Description;Acc:AAEL002130] | 0.082965 | 0.324108 | 0.348297 | 1.673522 |
| [AAEL002142](https://www.vectorbase.org/aedes_aegypti/Gene/Summary?db=core;g=AAEL002142) |  | 0.141494 | 0.528406 | 1.468431 | 1.919242 |
| [AAEL002184](https://www.vectorbase.org/aedes_aegypti/Gene/Summary?db=core;g=AAEL002184) | F-actin capping protein beta subunit [Source:VB External Description;Acc:AAEL002184] | 0.024927 | 0.3976 | 0.278644 | 0.781069 |
| [AAEL002217](https://www.vectorbase.org/aedes_aegypti/Gene/Summary?db=core;g=AAEL002217) |  | 0.188125 | 0.125742 | 0.960171 | 1.511106 |
| [AAEL002240](https://www.vectorbase.org/aedes_aegypti/Gene/Summary?db=core;g=AAEL002240) | signal transducing adapter molecule (stam) [Source:VB External Description;Acc:AAEL002240] | 0.255935 | 0.496674 | 0.850407 | 1.072897 |
| [AAEL002261](https://www.vectorbase.org/aedes_aegypti/Gene/Summary?db=core;g=AAEL002261) | GTP cyclohydrolase i [Source:VB External Description;Acc:AAEL002261] | -0.66651 | -0.36321 | -0.40749 | 2.130519 |
| [AAEL002263](https://www.vectorbase.org/aedes_aegypti/Gene/Summary?db=core;g=AAEL002263) |  | -0.25446 | 0.047518 | 0.371873 | 1.096379 |
| [AAEL002304](https://www.vectorbase.org/aedes_aegypti/Gene/Summary?db=core;g=AAEL002304) | porphobilinogen synthase [Source:VB External Description;Acc:AAEL002304] | -0.14855 | 0.201115 | 0.35973 | 0.963006 |
| [AAEL002309](https://www.vectorbase.org/aedes_aegypti/Gene/Summary?db=core;g=AAEL002309) | Thioredoxin Peroxidase. [Source:VB Community Annotation;Acc:AAEL002309] | -0.51465 | 0.539335 | 0.824725 | 2.237512 |
| [AAEL002329](https://www.vectorbase.org/aedes_aegypti/Gene/Summary?db=core;g=AAEL002329) | alpha-1,3-mannosyl-glycoprotein beta-1, 2-n-acetylglucosaminyltransferase [Source:VB External Description;Acc:AAEL002329] | 0.38129 | 0.757809 | 0.594895 | 0.733604 |
| [AAEL002332](https://www.vectorbase.org/aedes_aegypti/Gene/Summary?db=core;g=AAEL002332) |  | 0.454544 | 0.687068 | 0.824301 | 0.956582 |
| [AAEL002353](https://www.vectorbase.org/aedes_aegypti/Gene/Summary?db=core;g=AAEL002353) |  | 0.225569 | 0.575195 | 0.889648 | 0.945678 |
| [AAEL002499](https://www.vectorbase.org/aedes_aegypti/Gene/Summary?db=core;g=AAEL002499) |  | 0.681924 | 1.196837 | 1.110036 | 1.779433 |
| [AAEL002539](https://www.vectorbase.org/aedes_aegypti/Gene/Summary?db=core;g=AAEL002539) | fimbrin/plastin [Source:VB External Description;Acc:AAEL002539] | -0.00576 | 0.119028 | 0.510073 | 0.991121 |
| [AAEL002578](https://www.vectorbase.org/aedes_aegypti/Gene/Summary?db=core;g=AAEL002578) |  | -0.03436 | 0.111871 | 0.070874 | 0.875204 |
| [AAEL002589](https://www.vectorbase.org/aedes_aegypti/Gene/Summary?db=core;g=AAEL002589) |  | -0.04946 | 0.497621 | 0.623988 | 1.131785 |
| [AAEL002590](https://www.vectorbase.org/aedes_aegypti/Gene/Summary?db=core;g=AAEL002590) | serine protease, putative [Source:VB External Description;Acc:AAEL002590] | -0.22552 | 0.406029 | 0.746568 | 1.29695 |
| [AAEL002598](https://www.vectorbase.org/aedes_aegypti/Gene/Summary?db=core;g=AAEL002598) | odorant binding protein OBP15 [Source:VB Community Annotation;Acc:AAEL002598] | 0.120134 | 1.031603 | 1.377036 | 1.831538 |
| [AAEL002615](https://www.vectorbase.org/aedes_aegypti/Gene/Summary?db=core;g=AAEL002615) | leucine-rich transmembrane protein [Source:VB External Description;Acc:AAEL002615] | 0.381473 | 0.842786 | 0.942383 | 1.459093 |
| [AAEL002623](https://www.vectorbase.org/aedes_aegypti/Gene/Summary?db=core;g=AAEL002623) |  | -0.03451 | 0.364906 | 0.401486 | 0.965798 |
| [AAEL002630](https://www.vectorbase.org/aedes_aegypti/Gene/Summary?db=core;g=AAEL002630) |  | 0.438903 | 0.578686 | 0.420111 | 0.933995 |
| [AAEL002678](https://www.vectorbase.org/aedes_aegypti/Gene/Summary?db=core;g=AAEL002678) | ras suppressor protein 1, rsu1 [Source:VB External Description;Acc:AAEL002678] | 0.11214 | 0.421101 | 0.361362 | 0.691207 |
| [AAEL002701](https://www.vectorbase.org/aedes_aegypti/Gene/Summary?db=core;g=AAEL002701) | mannosyltransferase [Source:VB External Description;Acc:AAEL002701] | -0.05908 | 0.263552 | 0.283477 | 0.83717 |
| [AAEL002730](https://www.vectorbase.org/aedes_aegypti/Gene/Summary?db=core;g=AAEL002730) | Serine Protease Inhibitor (serpin) likely cleavage at R/V. [Source:VB Community Annotation;Acc:AAEL002730] | 0.366368 | 0.127393 | 0.004355 | 1.122763 |
| [AAEL002834](https://www.vectorbase.org/aedes_aegypti/Gene/Summary?db=core;g=AAEL002834) | myo-inositol-1 phosphate synthase [Source:VB External Description;Acc:AAEL002834] | 0.066768 | 0.439443 | 0.853334 | 0.820278 |
| [AAEL002848](https://www.vectorbase.org/aedes_aegypti/Gene/Summary?db=core;g=AAEL002848) | tubulin beta chain [Source:VB External Description;Acc:AAEL002848] | 0.118802 | 0.847118 | 0.916809 | 2.550217 |
| [AAEL002851](https://www.vectorbase.org/aedes_aegypti/Gene/Summary?db=core;g=AAEL002851) | tubulin beta chain [Source:VB External Description;Acc:AAEL002851] | 0.129507 | 0.465238 | 0.617302 | 1.180284 |
| [AAEL002875](https://www.vectorbase.org/aedes_aegypti/Gene/Summary?db=core;g=AAEL002875) |  | -0.08742 | 0.301683 | 0.858483 | 1.370269 |
| [AAEL002899](https://www.vectorbase.org/aedes_aegypti/Gene/Summary?db=core;g=AAEL002899) |  | 0.594748 | 1.504886 | 2.041105 | 1.863484 |
| [AAEL002900](https://www.vectorbase.org/aedes_aegypti/Gene/Summary?db=core;g=AAEL002900) |  | -0.06013 | -0.09317 | 0.242076 | 0.865822 |
| [AAEL002903](https://www.vectorbase.org/aedes_aegypti/Gene/Summary?db=core;g=AAEL002903) | phosphatidylinositol 3-kinase catalytic subunit alpha, beta, delta [Source:VB External Description;Acc:AAEL002903] | 0.387633 | 0.947219 | 1.714536 | 1.782666 |
| [AAEL002913](https://www.vectorbase.org/aedes_aegypti/Gene/Summary?db=core;g=AAEL002913) | peroxisomal membrane protein 70 abcd3 [Source:VB External Description;Acc:AAEL002913] | -0.00915 | 0.047331 | 0.116661 | 0.68916 |
| [AAEL002918](https://www.vectorbase.org/aedes_aegypti/Gene/Summary?db=core;g=AAEL002918) | centaurin beta [Source:VB External Description;Acc:AAEL002918] | 0.403143 | 1.093924 | 1.594698 | 1.880973 |
| [AAEL002935](https://www.vectorbase.org/aedes_aegypti/Gene/Summary?db=core;g=AAEL002935) |  | 0.358285 | 0.576576 | 0.602394 | 0.739541 |
| [AAEL002938](https://www.vectorbase.org/aedes_aegypti/Gene/Summary?db=core;g=AAEL002938) | skd/vacuolar sorting [Source:VB External Description;Acc:AAEL002938] | 0.158217 | 0.33091 | 0.483894 | 0.684461 |
| [AAEL002944](https://www.vectorbase.org/aedes_aegypti/Gene/Summary?db=core;g=AAEL002944) |  | 0.167878 | 0.4922 | 0.815368 | 1.332423 |
| [AAEL002963](https://www.vectorbase.org/aedes_aegypti/Gene/Summary?db=core;g=AAEL002963) |  | 0.404796 | 1.412598 | 1.531424 | 2.05142 |
| [AAEL002967](https://www.vectorbase.org/aedes_aegypti/Gene/Summary?db=core;g=AAEL002967) |  | 0.400555 | 1.511271 | 1.648698 | 1.948976 |
| [AAEL002979](https://www.vectorbase.org/aedes_aegypti/Gene/Summary?db=core;g=AAEL002979) |  | 0.524428 | 1.360475 | 1.667554 | 1.731687 |
| [AAEL003051](https://www.vectorbase.org/aedes_aegypti/Gene/Summary?db=core;g=AAEL003051) |  | 0.220401 | 1.149622 | 1.799256 | 2.378022 |
| [AAEL003075](https://www.vectorbase.org/aedes_aegypti/Gene/Summary?db=core;g=AAEL003075) |  | 0.275477 | 0.482728 | 0.51411 | 0.876643 |
| [AAEL003092](https://www.vectorbase.org/aedes_aegypti/Gene/Summary?db=core;g=AAEL003092) |  | 0.238782 | 0.07685 | 0.490305 | 0.896227 |
| [AAEL003157](https://www.vectorbase.org/aedes_aegypti/Gene/Summary?db=core;g=AAEL003157) | Protein SMG8 (Protein smg-8 homolog) [Source:VB External Description;Acc:AAEL003157] | 0.065536 | 0.165202 | 0.135044 | 0.636661 |
| [AAEL003190](https://www.vectorbase.org/aedes_aegypti/Gene/Summary?db=core;g=AAEL003190) |  | -0.27803 | 0.526894 | 0.749935 | 1.917367 |
| [AAEL003201](https://www.vectorbase.org/aedes_aegypti/Gene/Summary?db=core;g=AAEL003201) | Carboxy/choline esterase Alpha Esterase [Source:VB Community Annotation;Acc:AAEL003201] | 0.428857 | 1.053947 | 1.107123 | 0.868929 |
| [AAEL003246](https://www.vectorbase.org/aedes_aegypti/Gene/Summary?db=core;g=AAEL003246) | deoxyribose-phosphate aldolase [Source:VB External Description;Acc:AAEL003246] | -0.11239 | 0.291744 | 0.397866 | 1.002895 |
| [AAEL003261](https://www.vectorbase.org/aedes_aegypti/Gene/Summary?db=core;g=AAEL003261) |  | 0.571605 | 1.006845 | 0.925429 | 1.425554 |
| [AAEL003281](https://www.vectorbase.org/aedes_aegypti/Gene/Summary?db=core;g=AAEL003281) | ADP-ribosylation factor, arf [Source:VB External Description;Acc:AAEL003281] | -0.07537 | 0.131493 | 0.281274 | 0.913081 |
| [AAEL003343](https://www.vectorbase.org/aedes_aegypti/Gene/Summary?db=core;g=AAEL003343) |  | -0.53892 | 0.426119 | 0.977356 | 1.38487 |
| [AAEL003415](https://www.vectorbase.org/aedes_aegypti/Gene/Summary?db=core;g=AAEL003415) | lamin [Source:VB External Description;Acc:AAEL003415] | 0.123541 | 0.448411 | 0.414482 | 0.862474 |
| [AAEL003416](https://www.vectorbase.org/aedes_aegypti/Gene/Summary?db=core;g=AAEL003416) | sorting nexin [Source:VB External Description;Acc:AAEL003416] | 0.131022 | 0.14033 | 0.363644 | 0.86467 |
| [AAEL003439](https://www.vectorbase.org/aedes_aegypti/Gene/Summary?db=core;g=AAEL003439) | Caspase (Short). [Source:VB Community Annotation;Acc:AAEL003439] | 0.436939 | 0.68496 | 0.83708 | 2.302188 |
| [AAEL003462](https://www.vectorbase.org/aedes_aegypti/Gene/Summary?db=core;g=AAEL003462) | aromatic amino acid decarboxylase [Source:VB External Description;Acc:AAEL003462] | 0.691032 | 1.178337 | 1.586353 | 2.165528 |
| [AAEL003493](https://www.vectorbase.org/aedes_aegypti/Gene/Summary?db=core;g=AAEL003493) | GDI interacting protein, putative [Source:VB External Description;Acc:AAEL003493] | 0.249212 | 0.298232 | 0.356201 | 0.69256 |
| [AAEL003505](https://www.vectorbase.org/aedes_aegypti/Gene/Summary?db=core;g=AAEL003505) | jun [Source:VB External Description;Acc:AAEL003505] | 0.695565 | 1.221148 | 1.331151 | 1.841655 |
| [AAEL003567](https://www.vectorbase.org/aedes_aegypti/Gene/Summary?db=core;g=AAEL003567) |  | -0.12657 | 0.593984 | 0.944832 | 2.687152 |
| [AAEL003569](https://www.vectorbase.org/aedes_aegypti/Gene/Summary?db=core;g=AAEL003569) | acyl-coa thioesterase [Source:VB External Description;Acc:AAEL003569] | 0.121269 | 0.64263 | 0.733703 | 1.370294 |
| [AAEL003632](https://www.vectorbase.org/aedes_aegypti/Gene/Summary?db=core;g=AAEL003632) | Clip-Domain Serine Protease family B. [Source:VB Community Annotation;Acc:AAEL003632] | 0.267463 | 0.242755 | 1.690431 | 2.067175 |
| [AAEL003645](https://www.vectorbase.org/aedes_aegypti/Gene/Summary?db=core;g=AAEL003645) | stearoyl-coa desaturase [Source:VB External Description;Acc:AAEL003645] | -0.03812 | 0.444646 | 0.499766 | 1.036566 |
| [AAEL003664](https://www.vectorbase.org/aedes_aegypti/Gene/Summary?db=core;g=AAEL003664) | lupus la ribonucleoprotein [Source:VB External Description;Acc:AAEL003664] | 0.214771 | 0.40633 | 0.231808 | 0.762551 |
| [AAEL003709](https://www.vectorbase.org/aedes_aegypti/Gene/Summary?db=core;g=AAEL003709) | crotonobetainyl-CoA-hydratase, putative [Source:VB External Description;Acc:AAEL003709] | 0.04968 | 0.598925 | 0.582032 | 0.76792 |
| [AAEL003713](https://www.vectorbase.org/aedes_aegypti/Gene/Summary?db=core;g=AAEL003713) | leucine-rich transmembrane protein [Source:VB External Description;Acc:AAEL003713] | 0.122427 | 0.414906 | 0.588481 | 1.084293 |
| [AAEL003728](https://www.vectorbase.org/aedes_aegypti/Gene/Summary?db=core;g=AAEL003728) |  | 1.261204 | 0.735597 | 0.219058 | 1.603921 |
| [AAEL003754](https://www.vectorbase.org/aedes_aegypti/Gene/Summary?db=core;g=AAEL003754) | actin binding [Source:VB External Description;Acc:AAEL003754] | 0.178765 | 0.423995 | 0.423202 | 0.884976 |
| [AAEL003758](https://www.vectorbase.org/aedes_aegypti/Gene/Summary?db=core;g=AAEL003758) | sorting nexin [Source:VB External Description;Acc:AAEL003758] | 0.002775 | 0.495326 | 1.008805 | 1.45542 |
| [AAEL003817](https://www.vectorbase.org/aedes_aegypti/Gene/Summary?db=core;g=AAEL003817) | kappa b-ras [Source:VB External Description;Acc:AAEL003817] | 0.18935 | 0.561089 | 0.846281 | 1.32051 |
| [AAEL003883](https://www.vectorbase.org/aedes_aegypti/Gene/Summary?db=core;g=AAEL003883) |  | 0.030734 | -0.02072 | 0.259149 | 0.757573 |
| [AAEL003887](https://www.vectorbase.org/aedes_aegypti/Gene/Summary?db=core;g=AAEL003887) | vacuolar membrane protein pep11 [Source:VB External Description;Acc:AAEL003887] | 0.134406 | 0.324827 | 0.520171 | 0.97582 |
| [AAEL003951](https://www.vectorbase.org/aedes_aegypti/Gene/Summary?db=core;g=AAEL003951) |  | 0.552609 | 0.145219 | 0.897153 | 1.307882 |
| [AAEL003974](https://www.vectorbase.org/aedes_aegypti/Gene/Summary?db=core;g=AAEL003974) |  | 0.470436 | 0.052015 | 0.945451 | 1.286958 |
| [AAEL004010](https://www.vectorbase.org/aedes_aegypti/Gene/Summary?db=core;g=AAEL004010) | splicing factor, putative [Source:VB External Description;Acc:AAEL004010] | 0.321241 | 0.880493 | 0.554477 | 0.965402 |
| [AAEL004070](https://www.vectorbase.org/aedes_aegypti/Gene/Summary?db=core;g=AAEL004070) |  | 0.233384 | 0.433294 | 0.407747 | 0.821683 |
| [AAEL004090](https://www.vectorbase.org/aedes_aegypti/Gene/Summary?db=core;g=AAEL004090) |  | 0.347171 | 0.760026 | 1.850292 | 1.80256 |
| [AAEL004108](https://www.vectorbase.org/aedes_aegypti/Gene/Summary?db=core;g=AAEL004108) |  | 0.142194 | 0.473173 | 0.796127 | 1.300862 |
| [AAEL004112](https://www.vectorbase.org/aedes_aegypti/Gene/Summary?db=core;g=AAEL004112) | Thioredoxin Peroxidase. [Source:VB Community Annotation;Acc:AAEL004112] | 0.193601 | -0.07585 | -0.15655 | 1.300477 |
| [AAEL004147](https://www.vectorbase.org/aedes_aegypti/Gene/Summary?db=core;g=AAEL004147) | tetraspanin, putative [Source:VB External Description;Acc:AAEL004147] | 0.098838 | 0.345018 | 0.517991 | 1.199925 |
| [AAEL004209](https://www.vectorbase.org/aedes_aegypti/Gene/Summary?db=core;g=AAEL004209) | opioid-binding protein/cell adhesion molecule, putative [Source:VB External Description;Acc:AAEL004209] | -0.09067 | -0.09076 | 0.237507 | 1.92811 |
| [AAEL004214](https://www.vectorbase.org/aedes_aegypti/Gene/Summary?db=core;g=AAEL004214) |  | 0.356582 | 0.565321 | 1.155008 | 1.437878 |
| [AAEL004235](https://www.vectorbase.org/aedes_aegypti/Gene/Summary?db=core;g=AAEL004235) | kinesin-like protein Klp10A [Source:VB External Description;Acc:AAEL004235] | 0.169354 | 0.248746 | 0.727603 | 1.196513 |
| [AAEL004237](https://www.vectorbase.org/aedes_aegypti/Gene/Summary?db=core;g=AAEL004237) | vacuolar protein sorting 18 (deep orange protein) [Source:VB External Description;Acc:AAEL004237] | 0.0463 | 0.260111 | 0.330595 | 0.734762 |
| [AAEL004277](https://www.vectorbase.org/aedes_aegypti/Gene/Summary?db=core;g=AAEL004277) |  | 0.198215 | 0.442076 | 0.244582 | 0.716136 |
| [AAEL004311](https://www.vectorbase.org/aedes_aegypti/Gene/Summary?db=core;g=AAEL004311) |  | -0.0387 | 0.196312 | 0.242268 | 0.710669 |
| [AAEL004337](https://www.vectorbase.org/aedes_aegypti/Gene/Summary?db=core;g=AAEL004337) |  | -0.05299 | 0.030965 | 0.819392 | 1.268038 |
| [AAEL004345](https://www.vectorbase.org/aedes_aegypti/Gene/Summary?db=core;g=AAEL004345) | cysteinyl-tRNA synthetase [Source:VB External Description;Acc:AAEL004345] | -0.00098 | 0.129174 | 0.619431 | 1.179151 |
| [AAEL004455](https://www.vectorbase.org/aedes_aegypti/Gene/Summary?db=core;g=AAEL004455) |  | 0.258511 | 0.498598 | 0.306007 | 0.882801 |
| [AAEL004473](https://www.vectorbase.org/aedes_aegypti/Gene/Summary?db=core;g=AAEL004473) |  | 0.127281 | 0.323382 | 0.363624 | 0.85112 |
| [AAEL004486](https://www.vectorbase.org/aedes_aegypti/Gene/Summary?db=core;g=AAEL004486) | valacyclovir hydrolase [Source:VB External Description;Acc:AAEL004486] | 0.376357 | 0.768289 | 0.630264 | 0.909117 |
| [AAEL004488](https://www.vectorbase.org/aedes_aegypti/Gene/Summary?db=core;g=AAEL004488) |  | 0.060006 | 0.090816 | 0.516288 | 1.141463 |
| [AAEL004490](https://www.vectorbase.org/aedes_aegypti/Gene/Summary?db=core;g=AAEL004490) |  | 0.049525 | -0.21125 | 0.010806 | 0.776865 |
| [AAEL004504](https://www.vectorbase.org/aedes_aegypti/Gene/Summary?db=core;g=AAEL004504) | valacyclovir hydrolase [Source:VB External Description;Acc:AAEL004504] | 0.526115 | 0.614339 | 0.624421 | 1.123629 |
| [AAEL004513](https://www.vectorbase.org/aedes_aegypti/Gene/Summary?db=core;g=AAEL004513) | neurotransmitter gated ion channel [Source:VB External Description;Acc:AAEL004513] | -0.14117 | 0.219276 | 0.359227 | 0.879909 |
| [AAEL004549](https://www.vectorbase.org/aedes_aegypti/Gene/Summary?db=core;g=AAEL004549) | synapsin [Source:VB External Description;Acc:AAEL004549] | 0.584548 | 2.023988 | 1.886723 | 2.681324 |
| [AAEL004553](https://www.vectorbase.org/aedes_aegypti/Gene/Summary?db=core;g=AAEL004553) |  | 0.338284 | 0.706737 | 0.603395 | 0.846343 |
| [AAEL004589](https://www.vectorbase.org/aedes_aegypti/Gene/Summary?db=core;g=AAEL004589) | small calcium-binding mitochondrial carrier, putative [Source:VB External Description;Acc:AAEL004589] | 0.424057 | 1.351516 | 1.417607 | 2.227751 |
| [AAEL004668](https://www.vectorbase.org/aedes_aegypti/Gene/Summary?db=core;g=AAEL004668) | septin [Source:VB External Description;Acc:AAEL004668] | 0.208808 | 0.71569 | 0.874343 | 1.382462 |
| [AAEL004694](https://www.vectorbase.org/aedes_aegypti/Gene/Summary?db=core;g=AAEL004694) | munc13-4 [Source:VB External Description;Acc:AAEL004694] | 0.306031 | 0.707849 | 1.411337 | 1.853195 |
| [AAEL004738](https://www.vectorbase.org/aedes_aegypti/Gene/Summary?db=core;g=AAEL004738) | methionine aminopeptidase [Source:VB External Description;Acc:AAEL004738] | 0.080365 | 0.217316 | 0.376759 | 0.793546 |
| [AAEL004743](https://www.vectorbase.org/aedes_aegypti/Gene/Summary?db=core;g=AAEL004743) | multidrug resistance protein 2 (ATP-binding cassette protein c) [Source:VB External Description;Acc:AAEL004743] | 0.197056 | 0.48068 | 0.388505 | 0.926845 |
| [AAEL004778](https://www.vectorbase.org/aedes_aegypti/Gene/Summary?db=core;g=AAEL004778) | acyl-coa dehydrogenase [Source:VB External Description;Acc:AAEL004778] | -0.01132 | 0.205862 | 0.503166 | 0.791322 |
| [AAEL004786](https://www.vectorbase.org/aedes_aegypti/Gene/Summary?db=core;g=AAEL004786) |  | 0.198952 | 0.669017 | 0.650051 | 1.083118 |
| [AAEL004802](https://www.vectorbase.org/aedes_aegypti/Gene/Summary?db=core;g=AAEL004802) |  | 0.031963 | 0.207839 | 0.298834 | 0.647052 |
| [AAEL004803](https://www.vectorbase.org/aedes_aegypti/Gene/Summary?db=core;g=AAEL004803) |  | -0.36436 | -0.13431 | 0.410624 | 1.000424 |
| [AAEL004858](https://www.vectorbase.org/aedes_aegypti/Gene/Summary?db=core;g=AAEL004858) |  | 0.584682 | 0.897569 | 0.813334 | 1.422578 |
| [AAEL004874](https://www.vectorbase.org/aedes_aegypti/Gene/Summary?db=core;g=AAEL004874) | limd1 [Source:VB External Description;Acc:AAEL004874] | -0.04884 | 0.05481 | 0.37672 | 0.924303 |
| [AAEL004900](https://www.vectorbase.org/aedes_aegypti/Gene/Summary?db=core;g=AAEL004900) | geranylgeranyl pyrophosphate synthase [Source:VB External Description;Acc:AAEL004900] | 0.042596 | 0.187562 | 1.000132 | 1.607342 |
| [AAEL004964](https://www.vectorbase.org/aedes_aegypti/Gene/Summary?db=core;g=AAEL004964) |  | 0.144852 | 0.335545 | -0.08039 | 1.210243 |
| [AAEL004981](https://www.vectorbase.org/aedes_aegypti/Gene/Summary?db=core;g=AAEL004981) | cation-transporting ATPase [Source:VB External Description;Acc:AAEL004981] | 0.247595 | 0.364727 | 0.583431 | 1.018718 |
| [AAEL004993](https://www.vectorbase.org/aedes_aegypti/Gene/Summary?db=core;g=AAEL004993) |  | -0.01622 | 0.277388 | 0.1671 | 0.659993 |
| [AAEL005013](https://www.vectorbase.org/aedes_aegypti/Gene/Summary?db=core;g=AAEL005013) |  | -0.14714 | 0.165638 | 0.46131 | 0.725342 |
| [AAEL005016](https://www.vectorbase.org/aedes_aegypti/Gene/Summary?db=core;g=AAEL005016) |  | 0.499592 | 1.349836 | 1.0497 | 1.389924 |
| [AAEL005017](https://www.vectorbase.org/aedes_aegypti/Gene/Summary?db=core;g=AAEL005017) |  | 0.359047 | 0.582237 | 0.508076 | 0.79219 |
| [AAEL005026](https://www.vectorbase.org/aedes_aegypti/Gene/Summary?db=core;g=AAEL005026) | ATP-dependent bile acid permease [Source:VB External Description;Acc:AAEL005026] | 0.655366 | 0.724891 | 0.368181 | 1.292808 |
| [AAEL005114](https://www.vectorbase.org/aedes_aegypti/Gene/Summary?db=core;g=AAEL005114) | RNA and export factor binding protein [Source:VB External Description;Acc:AAEL005114] | 0.173144 | 0.444462 | 0.221393 | 0.696273 |
| [AAEL005156](https://www.vectorbase.org/aedes_aegypti/Gene/Summary?db=core;g=AAEL005156) |  | -0.14064 | 0.333539 | 0.679011 | 1.04425 |
| [AAEL005159](https://www.vectorbase.org/aedes_aegypti/Gene/Summary?db=core;g=AAEL005159) | latent nuclear antigen, putative [Source:VB External Description;Acc:AAEL005159] | -0.07344 | 0.803353 | 1.509493 | 1.917578 |
| [AAEL005193](https://www.vectorbase.org/aedes_aegypti/Gene/Summary?db=core;g=AAEL005193) |  | 0.555265 | 0.987758 | 0.834226 | 1.195279 |
| [AAEL005201](https://www.vectorbase.org/aedes_aegypti/Gene/Summary?db=core;g=AAEL005201) | hydroxymethylglutaryl-coa synthase [Source:VB External Description;Acc:AAEL005201] | -0.00784 | 0.128752 | 0.6605 | 0.771933 |
| [AAEL005254](https://www.vectorbase.org/aedes_aegypti/Gene/Summary?db=core;g=AAEL005254) | dynactin P62 subunit [Source:VB External Description;Acc:AAEL005254] | -0.01639 | 0.01177 | 0.20997 | 0.726011 |
| [AAEL005270](https://www.vectorbase.org/aedes_aegypti/Gene/Summary?db=core;g=AAEL005270) |  | -0.14634 | 0.496754 | 0.391967 | 1.180943 |
| [AAEL005271](https://www.vectorbase.org/aedes_aegypti/Gene/Summary?db=core;g=AAEL005271) |  | 0.331104 | 0.409866 | 0.754118 | 1.060656 |
| [AAEL005293](https://www.vectorbase.org/aedes_aegypti/Gene/Summary?db=core;g=AAEL005293) | galectin [Source:VB Community Annotation;Acc:AAEL005293] | -0.41212 | -0.5272 | 0.052556 | 0.933711 |
| [AAEL005294](https://www.vectorbase.org/aedes_aegypti/Gene/Summary?db=core;g=AAEL005294) | galectin [Source:VB Community Annotation;Acc:AAEL005294] | -0.07541 | 0.737771 | 1.014142 | 1.921213 |
| [AAEL005311](https://www.vectorbase.org/aedes_aegypti/Gene/Summary?db=core;g=AAEL005311) |  | -0.2298 | 0.070564 | 0.230436 | 0.858958 |
| [AAEL005331](https://www.vectorbase.org/aedes_aegypti/Gene/Summary?db=core;g=AAEL005331) |  | 0.268437 | 0.320211 | 0.466282 | 1.389664 |
| [AAEL005332](https://www.vectorbase.org/aedes_aegypti/Gene/Summary?db=core;g=AAEL005332) |  | 0.354305 | 0.276327 | 0.022223 | 1.505836 |
| [AAEL005339](https://www.vectorbase.org/aedes_aegypti/Gene/Summary?db=core;g=AAEL005339) | hepatocyte growth factor-regulated tyrosine kinase substrate (hgs) [Source:VB External Description;Acc:AAEL005339] | 0.187694 | 0.482721 | 0.629575 | 1.2587 |
| [AAEL005341](https://www.vectorbase.org/aedes_aegypti/Gene/Summary?db=core;g=AAEL005341) | Protein hook [Source:VB Community Annotation;Acc:AAEL005341] | -0.08801 | 0.11142 | 0.133534 | 0.608071 |
| [AAEL005384](https://www.vectorbase.org/aedes_aegypti/Gene/Summary?db=core;g=AAEL005384) | phosphoribosylformylglycinamidine synthase, putative [Source:VB External Description;Acc:AAEL005384] | 0.073613 | 0.172262 | 0.21203 | 1.258068 |
| [AAEL005407](https://www.vectorbase.org/aedes_aegypti/Gene/Summary?db=core;g=AAEL005407) | annexin x [Source:VB External Description;Acc:AAEL005407] | 0.190724 | 0.764603 | 1.70942 | 2.114246 |
| [AAEL005408](https://www.vectorbase.org/aedes_aegypti/Gene/Summary?db=core;g=AAEL005408) | annexin x [Source:VB External Description;Acc:AAEL005408] | -0.04275 | 0.837284 | 1.28038 | 2.116035 |
| [AAEL005417](https://www.vectorbase.org/aedes_aegypti/Gene/Summary?db=core;g=AAEL005417) | annexin x [Source:VB External Description;Acc:AAEL005417] | 0.304599 | 0.462213 | 0.792124 | 1.167593 |
| [AAEL005426](https://www.vectorbase.org/aedes_aegypti/Gene/Summary?db=core;g=AAEL005426) | annexin x [Source:VB External Description;Acc:AAEL005426] | 0.300189 | 0.617102 | 0.705768 | 0.964455 |
| [AAEL005428](https://www.vectorbase.org/aedes_aegypti/Gene/Summary?db=core;g=AAEL005428) |  | -0.36685 | 0.088331 | 0.304143 | 1.0615 |
| [AAEL005432](https://www.vectorbase.org/aedes_aegypti/Gene/Summary?db=core;g=AAEL005432) |  | 0.103778 | 0.341686 | 0.410963 | 0.906529 |
| [AAEL005446](https://www.vectorbase.org/aedes_aegypti/Gene/Summary?db=core;g=AAEL005446) | 6-phosphofructo-2-kinase/fructose-2,6-bisphosphatase [Source:VB External Description;Acc:AAEL005446] | 0.248179 | 0.584003 | 0.75193 | 0.460375 |
| [AAEL005472](https://www.vectorbase.org/aedes_aegypti/Gene/Summary?db=core;g=AAEL005472) | calpain [Source:VB External Description;Acc:AAEL005472] | 0.080265 | 0.365676 | 0.514682 | 1.035995 |
| [AAEL005491](https://www.vectorbase.org/aedes_aegypti/Gene/Summary?db=core;g=AAEL005491) | ABC transporter [Source:VB External Description;Acc:AAEL005491] | -0.0964 | -0.32522 | 1.576679 | 2.932877 |
| [AAEL005493](https://www.vectorbase.org/aedes_aegypti/Gene/Summary?db=core;g=AAEL005493) | septin [Source:VB External Description;Acc:AAEL005493] | 0.275023 | 0.534857 | 0.580555 | 0.884912 |
| [AAEL005513](https://www.vectorbase.org/aedes_aegypti/Gene/Summary?db=core;g=AAEL005513) | mothers against dpp protein [Source:VB External Description;Acc:AAEL005513] | 0.255978 | 0.927009 | 1.386332 | 1.709333 |
| [AAEL005600](https://www.vectorbase.org/aedes_aegypti/Gene/Summary?db=core;g=AAEL005600) |  | 0.346474 | 0.725148 | 0.716857 | 0.837884 |
| [AAEL005655](https://www.vectorbase.org/aedes_aegypti/Gene/Summary?db=core;g=AAEL005655) | sorting nexin [Source:VB External Description;Acc:AAEL005655] | 0.343204 | 1.126876 | 1.619333 | 2.077251 |
| [AAEL005701](https://www.vectorbase.org/aedes_aegypti/Gene/Summary?db=core;g=AAEL005701) | retinaldehyde binding protein [Source:VB External Description;Acc:AAEL005701] | 0.0928 | 0.139159 | 0.439958 | 0.750681 |
| [AAEL005704](https://www.vectorbase.org/aedes_aegypti/Gene/Summary?db=core;g=AAEL005704) |  | 0.122311 | 0.553154 | 0.586521 | 0.878982 |
| [AAEL005706](https://www.vectorbase.org/aedes_aegypti/Gene/Summary?db=core;g=AAEL005706) | triacylglycerol lipase [Source:VB External Description;Acc:AAEL005706] | 0.362907 | 0.253734 | 0.491414 | 0.97046 |
| [AAEL005739](https://www.vectorbase.org/aedes_aegypti/Gene/Summary?db=core;g=AAEL005739) |  | 0.114324 | 0.238759 | 0.446142 | 1.324952 |
| [AAEL005770](https://www.vectorbase.org/aedes_aegypti/Gene/Summary?db=core;g=AAEL005770) | odorant binding protein OBP21 [Source:VB Community Annotation;Acc:AAEL005770] | -0.12516 | 0.066152 | 0.074726 | 0.764662 |
| [AAEL005790](https://www.vectorbase.org/aedes_aegypti/Gene/Summary?db=core;g=AAEL005790) | malic enzyme [Source:VB External Description;Acc:AAEL005790] | 0.09663 | 0.587209 | 0.751351 | 1.531404 |
| [AAEL005872](https://www.vectorbase.org/aedes_aegypti/Gene/Summary?db=core;g=AAEL005872) |  | -0.02175 | 0.256086 | 0.428932 | 0.762018 |
| [AAEL005874](https://www.vectorbase.org/aedes_aegypti/Gene/Summary?db=core;g=AAEL005874) |  | -0.01724 | 0.103271 | 0.36431 | 0.910908 |
| [AAEL005879](https://www.vectorbase.org/aedes_aegypti/Gene/Summary?db=core;g=AAEL005879) | vinculin [Source:VB External Description;Acc:AAEL005879] | 0.051132 | 0.347345 | 0.297673 | 0.727931 |
| [AAEL005881](https://www.vectorbase.org/aedes_aegypti/Gene/Summary?db=core;g=AAEL005881) | autocrine motility factor receptor, amfr [Source:VB External Description;Acc:AAEL005881] | -0.07225 | 0.237814 | 0.271417 | 0.911534 |
| [AAEL005882](https://www.vectorbase.org/aedes_aegypti/Gene/Summary?db=core;g=AAEL005882) |  | 0.096934 | 0.562319 | 0.615294 | 1.023414 |
| [AAEL005910](https://www.vectorbase.org/aedes_aegypti/Gene/Summary?db=core;g=AAEL005910) | programmed cell death protein [Source:VB External Description;Acc:AAEL005910] | 0.244901 | 0.358843 | 0.371286 | 0.791211 |
| [AAEL005955](https://www.vectorbase.org/aedes_aegypti/Gene/Summary?db=core;g=AAEL005955) | caspase [Source:VB Community Annotation;Acc:AAEL005955] | 0.466622 | 1.041685 | 1.11355 | 1.726965 |
| [AAEL005968](https://www.vectorbase.org/aedes_aegypti/Gene/Summary?db=core;g=AAEL005968) | Ubiquitin-fold modifier-conjugating enzyme 1 (Ufm1-conjugating enzyme 1) [Source:VB External Description;Acc:AAEL005968] | -0.14057 | 0.095782 | 0.112142 | 0.669647 |
| [AAEL006001](https://www.vectorbase.org/aedes_aegypti/Gene/Summary?db=core;g=AAEL006001) |  | -0.23063 | -0.05754 | 0.279867 | 1.041698 |
| [AAEL006028](https://www.vectorbase.org/aedes_aegypti/Gene/Summary?db=core;g=AAEL006028) |  | -0.00231 | 0.113086 | 0.212238 | 0.986722 |
| [AAEL006029](https://www.vectorbase.org/aedes_aegypti/Gene/Summary?db=core;g=AAEL006029) | Probable dynactin subunit 2 [Source:VB Community Annotation;Acc:AAEL006029] | 0.209221 | 0.408627 | 0.290764 | 1.010404 |
| [AAEL006069](https://www.vectorbase.org/aedes_aegypti/Gene/Summary?db=core;g=AAEL006069) |  | 0.164726 | 0.426935 | 1.176708 | 1.865716 |
| [AAEL006097](https://www.vectorbase.org/aedes_aegypti/Gene/Summary?db=core;g=AAEL006097) |  | 0.113639 | 0.805275 | 1.107859 | 1.486231 |
| [AAEL006101](https://www.vectorbase.org/aedes_aegypti/Gene/Summary?db=core;g=AAEL006101) |  | 0.166779 | 0.867109 | 1.037068 | 1.335553 |
| [AAEL006133](https://www.vectorbase.org/aedes_aegypti/Gene/Summary?db=core;g=AAEL006133) | cofactor A, putative [Source:VB External Description;Acc:AAEL006133] | 0.074603 | 0.316497 | 0.332494 | 0.872967 |
| [AAEL006155](https://www.vectorbase.org/aedes_aegypti/Gene/Summary?db=core;g=AAEL006155) |  | 0.160308 | 0.433789 | 0.497458 | 0.795568 |
| [AAEL006231](https://www.vectorbase.org/aedes_aegypti/Gene/Summary?db=core;g=AAEL006231) | paraflagellar rod protein, putative [Source:VB External Description;Acc:AAEL006231] | 0.067675 | 0.216395 | 0.301218 | 0.697829 |
| [AAEL006233](https://www.vectorbase.org/aedes_aegypti/Gene/Summary?db=core;g=AAEL006233) |  | 0.101079 | 0.171701 | 0.152151 | 1.028762 |
| [AAEL006247](https://www.vectorbase.org/aedes_aegypti/Gene/Summary?db=core;g=AAEL006247) |  | 0.3788 | 0.37038 | 0.970268 | 1.024357 |
| [AAEL006276](https://www.vectorbase.org/aedes_aegypti/Gene/Summary?db=core;g=AAEL006276) | maltose phosphorylase [Source:VB External Description;Acc:AAEL006276] | -0.0259 | 0.415036 | 0.986557 | 1.656734 |
| [AAEL006321](https://www.vectorbase.org/aedes_aegypti/Gene/Summary?db=core;g=AAEL006321) | 1-acylglycerol-3-phosphate acyltransferase [Source:VB External Description;Acc:AAEL006321] | 0.534168 | 1.13656 | 1.426015 | 2.040185 |
| [AAEL006370](https://www.vectorbase.org/aedes_aegypti/Gene/Summary?db=core;g=AAEL006370) | amsh [Source:VB External Description;Acc:AAEL006370] | 0.213119 | 0.49407 | 0.596725 | 0.71236 |
| [AAEL006493](https://www.vectorbase.org/aedes_aegypti/Gene/Summary?db=core;g=AAEL006493) |  | 1.164185 | 1.766068 | 0.886335 | 4.236867 |
| [AAEL006523](https://www.vectorbase.org/aedes_aegypti/Gene/Summary?db=core;g=AAEL006523) | crk [Source:VB External Description;Acc:AAEL006523] | 0.009794 | 0.731138 | 1.199738 | 1.999761 |
| [AAEL006609](https://www.vectorbase.org/aedes_aegypti/Gene/Summary?db=core;g=AAEL006609) | zinc finger protein [Source:VB External Description;Acc:AAEL006609] | 0.016401 | 0.366769 | 0.481571 | 1.179203 |
| [AAEL006642](https://www.vectorbase.org/aedes_aegypti/Gene/Summary?db=core;g=AAEL006642) | tubulin alpha chain [Source:VB External Description;Acc:AAEL006642] | -0.12319 | 0.198057 | 0.592737 | 1.361467 |
| [AAEL006717](https://www.vectorbase.org/aedes_aegypti/Gene/Summary?db=core;g=AAEL006717) | ABC transporter [Source:VB External Description;Acc:AAEL006717] | -0.12635 | 0.154278 | 0.215691 | 0.68453 |
| [AAEL006786](https://www.vectorbase.org/aedes_aegypti/Gene/Summary?db=core;g=AAEL006786) | GTPase_rho [Source:VB External Description;Acc:AAEL006786] | 0.134009 | 0.344842 | 0.358049 | 0.73627 |
| [AAEL006795](https://www.vectorbase.org/aedes_aegypti/Gene/Summary?db=core;g=AAEL006795) | cytochrome P450 [Source:VB Community Annotation;Acc:AAEL006795] | 0.192721 | 0.308833 | 0.458833 | 0.820113 |
| [AAEL006824](https://www.vectorbase.org/aedes_aegypti/Gene/Summary?db=core;g=AAEL006824) | cytochrome P450 [Source:VB External Description;Acc:AAEL006824] | 1.190025 | 1.370392 | 0.460738 | 2.246169 |
| [AAEL006831](https://www.vectorbase.org/aedes_aegypti/Gene/Summary?db=core;g=AAEL006831) | achaete-scute complex protein T3, putative [Source:VB External Description;Acc:AAEL006831] | 1.063428 | 1.288179 | 0.986731 | 1.920145 |
| [AAEL006922](https://www.vectorbase.org/aedes_aegypti/Gene/Summary?db=core;g=AAEL006922) | calponin/transgelin [Source:VB External Description;Acc:AAEL006922] | 0.100043 | 0.432007 | 0.163655 | 0.827119 |
| [AAEL006968](https://www.vectorbase.org/aedes_aegypti/Gene/Summary?db=core;g=AAEL006968) |  | -0.98782 | -1.9286 | 1.048415 | 2.030535 |
| [AAEL006971](https://www.vectorbase.org/aedes_aegypti/Gene/Summary?db=core;g=AAEL006971) |  | 0.555839 | -1.12574 | 1.534965 | 3.631791 |
| [AAEL006987](https://www.vectorbase.org/aedes_aegypti/Gene/Summary?db=core;g=AAEL006987) |  | 0.365386 | 0.210377 | 0.579174 | 1.626103 |
| [AAEL007002](https://www.vectorbase.org/aedes_aegypti/Gene/Summary?db=core;g=AAEL007002) | calsenilin [Source:VB External Description;Acc:AAEL007002] | -0.24086 | 1.459384 | 1.44219 | 2.579855 |
| [AAEL007018](https://www.vectorbase.org/aedes_aegypti/Gene/Summary?db=core;g=AAEL007018) | udp-glucose 4-epimerase [Source:VB External Description;Acc:AAEL007018] | 0.115257 | 0.247374 | 0.68112 | 0.776967 |
| [AAEL007179](https://www.vectorbase.org/aedes_aegypti/Gene/Summary?db=core;g=AAEL007179) |  | 0.085067 | 0.568503 | 0.845729 | 1.278289 |
| [AAEL007209](https://www.vectorbase.org/aedes_aegypti/Gene/Summary?db=core;g=AAEL007209) | ADP-ribosylation factor, putative [Source:VB External Description;Acc:AAEL007209] | 0.108588 | 0.356015 | 0.396471 | 0.705896 |
| [AAEL007213](https://www.vectorbase.org/aedes_aegypti/Gene/Summary?db=core;g=AAEL007213) | delta(9)-desaturase, putative [Source:VB External Description;Acc:AAEL007213] | -1.76445 | 0.959466 | 1.940906 | 2.71683 |
| [AAEL007238](https://www.vectorbase.org/aedes_aegypti/Gene/Summary?db=core;g=AAEL007238) |  | -0.01209 | 0.436351 | 0.358701 | 1.14909 |
| [AAEL007246](https://www.vectorbase.org/aedes_aegypti/Gene/Summary?db=core;g=AAEL007246) |  | -0.06055 | 0.039055 | 0.296844 | 0.844531 |
| [AAEL007284](https://www.vectorbase.org/aedes_aegypti/Gene/Summary?db=core;g=AAEL007284) | serine/threonine-protein kinase pk61c [Source:VB External Description;Acc:AAEL007284] | 0.407222 | 0.690023 | 0.788048 | 1.092489 |
| [AAEL007309](https://www.vectorbase.org/aedes_aegypti/Gene/Summary?db=core;g=AAEL007309) |  | 0.207096 | 0.562158 | 0.194839 | 0.784996 |
| [AAEL007322](https://www.vectorbase.org/aedes_aegypti/Gene/Summary?db=core;g=AAEL007322) | phosphatidate phosphatase [Source:VB External Description;Acc:AAEL007322] | 0.130568 | 0.424436 | 0.709873 | 0.759096 |
| [AAEL007324](https://www.vectorbase.org/aedes_aegypti/Gene/Summary?db=core;g=AAEL007324) |  | 0.006766 | 0.462435 | 0.586563 | 1.212952 |
| [AAEL007344](https://www.vectorbase.org/aedes_aegypti/Gene/Summary?db=core;g=AAEL007344) |  | -0.0677 | 0.326301 | 1.013345 | 1.673248 |
| [AAEL007349](https://www.vectorbase.org/aedes_aegypti/Gene/Summary?db=core;g=AAEL007349) |  | -0.1785 | 0.055928 | -0.08138 | 0.701813 |
| [AAEL007387](https://www.vectorbase.org/aedes_aegypti/Gene/Summary?db=core;g=AAEL007387) |  | -0.13637 | -0.18024 | 1.114249 | 1.87109 |
| [AAEL007509](https://www.vectorbase.org/aedes_aegypti/Gene/Summary?db=core;g=AAEL007509) | neuroendocrine differentiation factor [Source:VB External Description;Acc:AAEL007509] | -0.02744 | 0.204607 | 0.098064 | 0.646611 |
| [AAEL007510](https://www.vectorbase.org/aedes_aegypti/Gene/Summary?db=core;g=AAEL007510) |  | 0.924146 | 0.374141 | 1.041441 | 1.328129 |
| [AAEL007522](https://www.vectorbase.org/aedes_aegypti/Gene/Summary?db=core;g=AAEL007522) | goodpasture antigen-binding protein [Source:VB External Description;Acc:AAEL007522] | 0.374314 | 0.742974 | 0.506129 | 0.760795 |
| [AAEL007537](https://www.vectorbase.org/aedes_aegypti/Gene/Summary?db=core;g=AAEL007537) |  | -0.0318 | 0.235047 | 0.476263 | 1.112573 |
| [AAEL007616](https://www.vectorbase.org/aedes_aegypti/Gene/Summary?db=core;g=AAEL007616) |  | -0.08608 | 0.164988 | 0.418128 | 0.801876 |
| [AAEL007624](https://www.vectorbase.org/aedes_aegypti/Gene/Summary?db=core;g=AAEL007624) | IMD pathway signalling NF-kappaB Relish-like transcription factor [Source:VB Community Annotation;Acc:AAEL007624] | 0.262366 | 0.686995 | 0.739058 | 1.139259 |
| [AAEL007637](https://www.vectorbase.org/aedes_aegypti/Gene/Summary?db=core;g=AAEL007637) | AAA ATPase [Source:VB External Description;Acc:AAEL007637] | -0.04566 | 0.271092 | 0.299365 | 0.642949 |
| [AAEL007642](https://www.vectorbase.org/aedes_aegypti/Gene/Summary?db=core;g=AAEL007642) | TOLL pathway signalling. [Source:VB Community Annotation;Acc:AAEL007642] | 0.096639 | 0.386321 | 0.662123 | 1.063838 |
| [AAEL007643](https://www.vectorbase.org/aedes_aegypti/Gene/Summary?db=core;g=AAEL007643) | Molybdopterin synthase sulfur carrier subunit (Sulfur carrier protein MOCS2A)(Molybdenum cofactor synthesis protein 2A)(MOCS2A)(Molybdenum cofactor synthesis protein 2 small subunit) [Source:VB External Description;Acc:AAEL007643] | -0.12793 | -0.23677 | 0.141767 | 0.784284 |
| [AAEL007693](https://www.vectorbase.org/aedes_aegypti/Gene/Summary?db=core;g=AAEL007693) | organic anion transporter [Source:VB External Description;Acc:AAEL007693] | -0.08862 | -0.02745 | 0.338012 | 0.754888 |
| [AAEL007758](https://www.vectorbase.org/aedes_aegypti/Gene/Summary?db=core;g=AAEL007758) |  | 0.020937 | 0.673577 | 0.540382 | 1.63008 |
| [AAEL007765](https://www.vectorbase.org/aedes_aegypti/Gene/Summary?db=core;g=AAEL007765) | Serine Protease Inhibitor (serpin) likely cleavage at K/R. Transcript A. [Source:VB Community Annotation;Acc:AAEL007765] | -0.10046 | 0.096781 | 0.590708 | 1.236238 |
| [AAEL007773](https://www.vectorbase.org/aedes_aegypti/Gene/Summary?db=core;g=AAEL007773) |  | 0.126908 | 0.654813 | 0.677893 | 1.370564 |
| [AAEL007799](https://www.vectorbase.org/aedes_aegypti/Gene/Summary?db=core;g=AAEL007799) | regulator of chromosome condensation [Source:VB External Description;Acc:AAEL007799] | 0.308542 | 0.522984 | 0.481309 | 1.085401 |
| [AAEL007905](https://www.vectorbase.org/aedes_aegypti/Gene/Summary?db=core;g=AAEL007905) |  | 0.323249 | 0.66582 | 1.193347 | 1.584326 |
| [AAEL007909](https://www.vectorbase.org/aedes_aegypti/Gene/Summary?db=core;g=AAEL007909) | branched-chain amino acid aminotransferase [Source:VB External Description;Acc:AAEL007909] | 0.226466 | 0.592066 | 1.239965 | 1.533616 |
| [AAEL007920](https://www.vectorbase.org/aedes_aegypti/Gene/Summary?db=core;g=AAEL007920) |  | 0.294386 | 0.599662 | 0.41663 | 0.713619 |
| [AAEL007923](https://www.vectorbase.org/aedes_aegypti/Gene/Summary?db=core;g=AAEL007923) | eukaryotic translation initiation factor 4 gamma [Source:VB External Description;Acc:AAEL007923] | 0.311753 | 0.603736 | 0.958742 | 1.250502 |
| [AAEL007927](https://www.vectorbase.org/aedes_aegypti/Gene/Summary?db=core;g=AAEL007927) |  | 0.34165 | 0.623839 | 1.219717 | 1.319272 |
| [AAEL007928](https://www.vectorbase.org/aedes_aegypti/Gene/Summary?db=core;g=AAEL007928) | eukaryotic translation initiation factor 4 gamma [Source:VB External Description;Acc:AAEL007928] | 0.090172 | 0.406678 | 0.938595 | 1.558642 |
| [AAEL007993](https://www.vectorbase.org/aedes_aegypti/Gene/Summary?db=core;g=AAEL007993) | Clip-Domain Serine Protease family B. [Source:VB Community Annotation;Acc:AAEL007993] | 0.081832 | 0.013604 | -0.18076 | 2.016154 |
| [AAEL008039](https://www.vectorbase.org/aedes_aegypti/Gene/Summary?db=core;g=AAEL008039) |  | 0.081627 | 0.0895 | 0.094784 | 0.925406 |
| [AAEL008065](https://www.vectorbase.org/aedes_aegypti/Gene/Summary?db=core;g=AAEL008065) |  | 0.135857 | 0.536519 | 0.430377 | 0.819976 |
| [AAEL008079](https://www.vectorbase.org/aedes_aegypti/Gene/Summary?db=core;g=AAEL008079) | trypsin-alpha, putative [Source:VB External Description;Acc:AAEL008079] | -0.31566 | 0.411064 | 0.160788 | 0.864412 |
| [AAEL008106](https://www.vectorbase.org/aedes_aegypti/Gene/Summary?db=core;g=AAEL008106) |  | 0.692127 | 0.197397 | 1.361515 | 2.28 |
| [AAEL008139](https://www.vectorbase.org/aedes_aegypti/Gene/Summary?db=core;g=AAEL008139) |  | 0.182691 | 0.312482 | 0.329035 | 0.778504 |
| [AAEL008176](https://www.vectorbase.org/aedes_aegypti/Gene/Summary?db=core;g=AAEL008176) |  | -0.09568 | 0.104192 | 0.836564 | 1.270256 |
| [AAEL008181](https://www.vectorbase.org/aedes_aegypti/Gene/Summary?db=core;g=AAEL008181) | alpha6-fucosyltransferase, putative [Source:VB External Description;Acc:AAEL008181] | -0.04425 | 0.209292 | 0.251283 | 0.610742 |
| [AAEL008185](https://www.vectorbase.org/aedes_aegypti/Gene/Summary?db=core;g=AAEL008185) |  | 0.339739 | 0.677904 | 0.757782 | 1.23172 |
| [AAEL008267](https://www.vectorbase.org/aedes_aegypti/Gene/Summary?db=core;g=AAEL008267) | GPCR Neurokinin/Tachykinin Family [Source:VB Community Annotation;Acc:AAEL008267] | 0.162294 | 0.059505 | 0.703622 | 0.918377 |
| [AAEL008346](https://www.vectorbase.org/aedes_aegypti/Gene/Summary?db=core;g=AAEL008346) | achaete-scute complex protein T3, putative [Source:VB External Description;Acc:AAEL008346] | 0.823844 | 0.728066 | 0.761191 | 1.648857 |
| [AAEL008388](https://www.vectorbase.org/aedes_aegypti/Gene/Summary?db=core;g=AAEL008388) | ATP-binding cassette sub-family A member 3, putative [Source:VB External Description;Acc:AAEL008388] | 0.459776 | 0.911707 | 1.08374 | 1.442835 |
| [AAEL008393](https://www.vectorbase.org/aedes_aegypti/Gene/Summary?db=core;g=AAEL008393) | phosphatidylserine synthase [Source:VB External Description;Acc:AAEL008393] | 0.233249 | 0.692842 | 0.606468 | 0.745413 |
| [AAEL008411](https://www.vectorbase.org/aedes_aegypti/Gene/Summary?db=core;g=AAEL008411) | serine collagenase 1 precursor, putative [Source:VB External Description;Acc:AAEL008411] | -0.27055 | 0.058475 | 0.225897 | 1.227846 |
| [AAEL008473](https://www.vectorbase.org/aedes_aegypti/Gene/Summary?db=core;g=AAEL008473) | cysteine-rich venom protein, putative [Source:VB External Description;Acc:AAEL008473] | 0.064654 | 0.400669 | 0.614861 | 1.556409 |
| [AAEL008480](https://www.vectorbase.org/aedes_aegypti/Gene/Summary?db=core;g=AAEL008480) |  | 0.36173 | 0.545375 | 0.417905 | 0.793799 |
| [AAEL008497](https://www.vectorbase.org/aedes_aegypti/Gene/Summary?db=core;g=AAEL008497) |  | 0.399293 | 0.765334 | 0.789945 | 1.704355 |
| [AAEL008528](https://www.vectorbase.org/aedes_aegypti/Gene/Summary?db=core;g=AAEL008528) | protein tyrosine phosphatase n11 (shp2) [Source:VB External Description;Acc:AAEL008528] | -0.01454 | 0.330014 | 0.786963 | 1.798148 |
| [AAEL008551](https://www.vectorbase.org/aedes_aegypti/Gene/Summary?db=core;g=AAEL008551) |  | -0.11662 | 0.183862 | 1.207107 | 2.204418 |
| [AAEL008596](https://www.vectorbase.org/aedes_aegypti/Gene/Summary?db=core;g=AAEL008596) | spaetzle-like cytokine [Source:VB Community Annotation;Acc:AAEL008596] | -1.34434 | 1.609271 | 1.774767 | 3.71255 |
| [AAEL008622](https://www.vectorbase.org/aedes_aegypti/Gene/Summary?db=core;g=AAEL008622) | jnk [Source:VB External Description;Acc:AAEL008622] | 0.738011 | 1.381365 | 2.441457 | 2.475478 |
| [AAEL008630](https://www.vectorbase.org/aedes_aegypti/Gene/Summary?db=core;g=AAEL008630) | GTP-binding protein alpha subunit, gna [Source:VB External Description;Acc:AAEL008630] | 0.233041 | 0.966308 | 1.174832 | 1.631784 |
| [AAEL008651](https://www.vectorbase.org/aedes_aegypti/Gene/Summary?db=core;g=AAEL008651) |  | -0.08882 | 0.354642 | 0.118563 | 1.513414 |
| [AAEL008671](https://www.vectorbase.org/aedes_aegypti/Gene/Summary?db=core;g=AAEL008671) |  | 0.234201 | 0.535332 | 0.388962 | 0.914378 |
| [AAEL008677](https://www.vectorbase.org/aedes_aegypti/Gene/Summary?db=core;g=AAEL008677) | grb2-associated binder, gab [Source:VB External Description;Acc:AAEL008677] | 0.036773 | 0.537338 | 1.251772 | 1.779009 |
| [AAEL008686](https://www.vectorbase.org/aedes_aegypti/Gene/Summary?db=core;g=AAEL008686) |  | 0.120504 | 0.276624 | 0.675922 | 1.058933 |
| [AAEL008687](https://www.vectorbase.org/aedes_aegypti/Gene/Summary?db=core;g=AAEL008687) | loquacious [Source:VB Community Annotation;Acc:AAEL008687] | 0.279368 | 0.793761 | 0.548789 | 0.930765 |
| [AAEL008688](https://www.vectorbase.org/aedes_aegypti/Gene/Summary?db=core;g=AAEL008688) | G-protein signalling modulator [Source:VB External Description;Acc:AAEL008688] | 0.18104 | 0.551863 | 0.61719 | 0.833617 |
| [AAEL008849](https://www.vectorbase.org/aedes_aegypti/Gene/Summary?db=core;g=AAEL008849) | selenophosphate synthase [Source:VB External Description;Acc:AAEL008849] | 0.173406 | 0.762773 | 0.443304 | 0.868517 |
| [AAEL008862](https://www.vectorbase.org/aedes_aegypti/Gene/Summary?db=core;g=AAEL008862) |  | 0.114736 | 0.270236 | 0.295506 | 0.753236 |
| [AAEL008921](https://www.vectorbase.org/aedes_aegypti/Gene/Summary?db=core;g=AAEL008921) | myosin regulatory light chain 2 smooth muscle [Source:VB External Description;Acc:AAEL008921] | -0.11104 | -0.01696 | 0.195584 | 0.911551 |
| [AAEL008953](https://www.vectorbase.org/aedes_aegypti/Gene/Summary?db=core;g=AAEL008953) |  | 0.464054 | 0.996671 | 1.225796 | 1.759231 |
| [AAEL008954](https://www.vectorbase.org/aedes_aegypti/Gene/Summary?db=core;g=AAEL008954) |  | 0.174663 | 0.434119 | 0.441734 | 0.757305 |
| [AAEL009037](https://www.vectorbase.org/aedes_aegypti/Gene/Summary?db=core;g=AAEL009037) | GTP-binding protein (i) alpha subunit, gnai [Source:VB External Description;Acc:AAEL009037] | -0.02188 | 0.344094 | 0.685023 | 1.170358 |
| [AAEL009048](https://www.vectorbase.org/aedes_aegypti/Gene/Summary?db=core;g=AAEL009048) |  | 0.269346 | 0.658969 | 0.666339 | 0.785389 |
| [AAEL009055](https://www.vectorbase.org/aedes_aegypti/Gene/Summary?db=core;g=AAEL009055) | gliotactin [Source:VB External Description;Acc:AAEL009055] | 0.314391 | 0.676527 | 0.67965 | 0.855736 |
| [AAEL009059](https://www.vectorbase.org/aedes_aegypti/Gene/Summary?db=core;g=AAEL009059) | arp2/3 complex 16 kd subunit (P16-arc) [Source:VB External Description;Acc:AAEL009059] | 0.001955 | 0.151238 | 0.064483 | 0.72581 |
| [AAEL009074](https://www.vectorbase.org/aedes_aegypti/Gene/Summary?db=core;g=AAEL009074) | Inhibitor of Apoptosis (IAP) containing Baculoviral IAP Repeat(s) (BIR domains). [Source:VB Community Annotation;Acc:AAEL009074] | 0.043645 | 0.170993 | 0.5557 | 0.824326 |
| [AAEL009079](https://www.vectorbase.org/aedes_aegypti/Gene/Summary?db=core;g=AAEL009079) |  | 0.110062 | 0.009119 | 0.400298 | 0.732166 |
| [AAEL009082](https://www.vectorbase.org/aedes_aegypti/Gene/Summary?db=core;g=AAEL009082) |  | 0.377793 | 0.459786 | 0.799636 | 1.033458 |
| [AAEL009085](https://www.vectorbase.org/aedes_aegypti/Gene/Summary?db=core;g=AAEL009085) | rho/rac/cdc GTPase-activating protein [Source:VB External Description;Acc:AAEL009085] | 0.255687 | 0.524716 | 0.588567 | 0.86305 |
| [AAEL009094](https://www.vectorbase.org/aedes_aegypti/Gene/Summary?db=core;g=AAEL009094) | WSCD family member AAEL009094 [Source:VB External Description;Acc:AAEL009094] | 0.19319 | 0.117724 | 0.986712 | 1.795576 |
| [AAEL009154](https://www.vectorbase.org/aedes_aegypti/Gene/Summary?db=core;g=AAEL009154) | glutathione synthetase [Source:VB External Description;Acc:AAEL009154] | 0.113691 | 0.493864 | 0.258088 | 0.768367 |
| [AAEL009224](https://www.vectorbase.org/aedes_aegypti/Gene/Summary?db=core;g=AAEL009224) |  | 0.133832 | 0.483791 | 0.382793 | 0.923214 |
| [AAEL009249](https://www.vectorbase.org/aedes_aegypti/Gene/Summary?db=core;g=AAEL009249) | coronin [Source:VB External Description;Acc:AAEL009249] | 0.09758 | 0.489058 | 0.629427 | 1.060738 |
| [AAEL009273](https://www.vectorbase.org/aedes_aegypti/Gene/Summary?db=core;g=AAEL009273) | inosine-5-monophosphate dehydrogenase [Source:VB External Description;Acc:AAEL009273] | 0.502167 | 0.682398 | 0.424222 | 0.953728 |
| [AAEL009280](https://www.vectorbase.org/aedes_aegypti/Gene/Summary?db=core;g=AAEL009280) |  | 0.198371 | 0.647493 | 0.493761 | 1.005877 |
| [AAEL009295](https://www.vectorbase.org/aedes_aegypti/Gene/Summary?db=core;g=AAEL009295) | lachesin [Source:VB External Description;Acc:AAEL009295] | 0.434938 | 0.752119 | 1.036482 | 1.318726 |
| [AAEL009302](https://www.vectorbase.org/aedes_aegypti/Gene/Summary?db=core;g=AAEL009302) | Protein CLP1 homolog [Source:VB External Description;Acc:AAEL009302] | -0.27708 | 0.039699 | 0.107135 | 0.88324 |
| [AAEL009317](https://www.vectorbase.org/aedes_aegypti/Gene/Summary?db=core;g=AAEL009317) | rab11 [Source:VB External Description;Acc:AAEL009317] | 0.158847 | 0.523792 | 0.469812 | 0.780834 |
| [AAEL009371](https://www.vectorbase.org/aedes_aegypti/Gene/Summary?db=core;g=AAEL009371) |  | -0.05196 | 0.020881 | 0.367383 | 1.084115 |
| [AAEL009483](https://www.vectorbase.org/aedes_aegypti/Gene/Summary?db=core;g=AAEL009483) |  | 0.025334 | 0.456771 | 0.495581 | 1.0693 |
| [AAEL009509](https://www.vectorbase.org/aedes_aegypti/Gene/Summary?db=core;g=AAEL009509) | zinc finger protein [Source:VB External Description;Acc:AAEL009509] | 0.118107 | 0.575376 | 0.742245 | 0.818823 |
| [AAEL009510](https://www.vectorbase.org/aedes_aegypti/Gene/Summary?db=core;g=AAEL009510) | glucosamine-fructose-6-phosphate aminotransferase [Source:VB External Description;Acc:AAEL009510] | -0.01592 | 0.283411 | 0.559267 | 0.784458 |
| [AAEL009536](https://www.vectorbase.org/aedes_aegypti/Gene/Summary?db=core;g=AAEL009536) |  | 0.482886 | 0.596834 | 0.627615 | 0.868476 |
| [AAEL009537](https://www.vectorbase.org/aedes_aegypti/Gene/Summary?db=core;g=AAEL009537) |  | -0.04454 | 0.39475 | 0.676097 | 1.170578 |
| [AAEL009574](https://www.vectorbase.org/aedes_aegypti/Gene/Summary?db=core;g=AAEL009574) | elongase, putative [Source:VB External Description;Acc:AAEL009574] | 0.154183 | 0.743778 | 1.567481 | 1.860606 |
| [AAEL009677](https://www.vectorbase.org/aedes_aegypti/Gene/Summary?db=core;g=AAEL009677) | membrane-associated protein gex-3 [Source:VB External Description;Acc:AAEL009677] | 0.370301 | 0.690684 | 0.465101 | 0.795766 |
| [AAEL009759](https://www.vectorbase.org/aedes_aegypti/Gene/Summary?db=core;g=AAEL009759) |  | 0.091163 | 0.599047 | 0.849674 | 1.087109 |
| [AAEL009832](https://www.vectorbase.org/aedes_aegypti/Gene/Summary?db=core;g=AAEL009832) | exocyst complex protein exo70 [Source:VB External Description;Acc:AAEL009832] | 0.047515 | 0.261647 | 0.311753 | 0.734048 |
| [AAEL009850](https://www.vectorbase.org/aedes_aegypti/Gene/Summary?db=core;g=AAEL009850) | galectin [Source:VB Community Annotation;Acc:AAEL009850] | 0.41964 | 1.00216 | 1.081757 | 1.751982 |
| [AAEL009877](https://www.vectorbase.org/aedes_aegypti/Gene/Summary?db=core;g=AAEL009877) | amyloid binding protein [Source:VB External Description;Acc:AAEL009877] | 0.152106 | 0.393784 | 0.914052 | 0.998213 |
| [AAEL009894](https://www.vectorbase.org/aedes_aegypti/Gene/Summary?db=core;g=AAEL009894) | leucine-rich immune protein (Coil-less) [Source:VB Community Annotation;Acc:AAEL009894] | 0.29028 | 0.494629 | 0.262622 | 0.852932 |
| [AAEL009931](https://www.vectorbase.org/aedes_aegypti/Gene/Summary?db=core;g=AAEL009931) | arsenite inducuble RNA associated protein aip-1 [Source:VB External Description;Acc:AAEL009931] | 0.130882 | 0.592676 | 1.297902 | 1.854178 |
| [AAEL009949](https://www.vectorbase.org/aedes_aegypti/Gene/Summary?db=core;g=AAEL009949) | homeotic antennapedia protein, putative [Source:VB External Description;Acc:AAEL009949] | 0.219098 | 0.144254 | 0.852274 | 1.314342 |
| [AAEL009952](https://www.vectorbase.org/aedes_aegypti/Gene/Summary?db=core;g=AAEL009952) |  | 0.481257 | 0.861773 | 0.820575 | 1.083661 |
| [AAEL010000](https://www.vectorbase.org/aedes_aegypti/Gene/Summary?db=core;g=AAEL010000) | ganglioside induced differentiation associated protein [Source:VB External Description;Acc:AAEL010000] | 0.303344 | 0.500881 | 0.614416 | 0.968721 |
| [AAEL010065](https://www.vectorbase.org/aedes_aegypti/Gene/Summary?db=core;g=AAEL010065) | protein disulfide-isomerase A6 precursor [Source:VB External Description;Acc:AAEL010065] | -0.10479 | 0.322855 | 0.45842 | 0.910915 |
| [AAEL010070](https://www.vectorbase.org/aedes_aegypti/Gene/Summary?db=core;g=AAEL010070) | guanine nucleotide-binding protein beta 5 (g protein beta5) [Source:VB External Description;Acc:AAEL010070] | 0.158376 | 0.489897 | 0.28388 | 0.701975 |
| [AAEL010248](https://www.vectorbase.org/aedes_aegypti/Gene/Summary?db=core;g=AAEL010248) | fibrillarin [Source:VB External Description;Acc:AAEL010248] | 0.349457 | 0.592089 | 0.237762 | 0.750364 |
| [AAEL010301](https://www.vectorbase.org/aedes_aegypti/Gene/Summary?db=core;g=AAEL010301) |  | 0.574226 | 0.155644 | 0.9644 | 1.239279 |
| [AAEL010321](https://www.vectorbase.org/aedes_aegypti/Gene/Summary?db=core;g=AAEL010321) | porphobilinogen deaminase [Source:VB External Description;Acc:AAEL010321] | 0.163679 | 0.471129 | 0.381027 | 0.696457 |
| [AAEL010360](https://www.vectorbase.org/aedes_aegypti/Gene/Summary?db=core;g=AAEL010360) | Cytosolic Fe-S cluster assembly factor NUBP1 homolog [Source:VB External Description;Acc:AAEL010360] | -0.28912 | -0.37522 | 0.327391 | 1.051319 |
| [AAEL010411](https://www.vectorbase.org/aedes_aegypti/Gene/Summary?db=core;g=AAEL010411) | dual specificity protein phosphatase [Source:VB External Description;Acc:AAEL010411] | 0.334949 | 0.792875 | 0.824353 | 1.082561 |
| [AAEL010432](https://www.vectorbase.org/aedes_aegypti/Gene/Summary?db=core;g=AAEL010432) | exocyst complex-subunit protein, 84kD-subunit, putative [Source:VB External Description;Acc:AAEL010432] | 0.063413 | 0.068452 | 0.420135 | 0.907892 |
| [AAEL010449](https://www.vectorbase.org/aedes_aegypti/Gene/Summary?db=core;g=AAEL010449) | huntingtin interacting protein [Source:VB External Description;Acc:AAEL010449] | 0.02394 | 0.368235 | 0.680451 | 1.067826 |
| [AAEL010504](https://www.vectorbase.org/aedes_aegypti/Gene/Summary?db=core;g=AAEL010504) |  | 0.294296 | 0.768647 | 0.753365 | 1.256209 |
| [AAEL010516](https://www.vectorbase.org/aedes_aegypti/Gene/Summary?db=core;g=AAEL010516) | autophagy related gene [Source:VB Community Annotation;Acc:AAEL010516] | 0.246613 | 0.348141 | 0.503055 | 0.73649 |
| [AAEL010543](https://www.vectorbase.org/aedes_aegypti/Gene/Summary?db=core;g=AAEL010543) |  | -0.00612 | 0.146759 | 0.70111 | 0.845565 |
| [AAEL010558](https://www.vectorbase.org/aedes_aegypti/Gene/Summary?db=core;g=AAEL010558) |  | 0.088283 | 0.305914 | 0.303877 | 1.168582 |
| [AAEL010581](https://www.vectorbase.org/aedes_aegypti/Gene/Summary?db=core;g=AAEL010581) | zinc finger protein, putative [Source:VB External Description;Acc:AAEL010581] | -0.07637 | -0.04304 | 0.542022 | 1.32429 |
| [AAEL010584](https://www.vectorbase.org/aedes_aegypti/Gene/Summary?db=core;g=AAEL010584) | vesicular mannose-binding lectin [Source:VB External Description;Acc:AAEL010584] | 0.110145 | 0.277492 | 0.589933 | 0.913763 |
| [AAEL010610](https://www.vectorbase.org/aedes_aegypti/Gene/Summary?db=core;g=AAEL010610) | serine palmitoyltransferase i [Source:VB External Description;Acc:AAEL010610] | 0.098823 | 0.457198 | 0.606063 | 1.186958 |
| [AAEL010652](https://www.vectorbase.org/aedes_aegypti/Gene/Summary?db=core;g=AAEL010652) |  | -0.01303 | -0.24761 | -0.00416 | 1.227099 |
| [AAEL010661](https://www.vectorbase.org/aedes_aegypti/Gene/Summary?db=core;g=AAEL010661) | phospholipid scramblase 1, [Source:VB External Description;Acc:AAEL010661] | 0.118812 | 0.456907 | 0.211544 | 0.853429 |
| [AAEL010667](https://www.vectorbase.org/aedes_aegypti/Gene/Summary?db=core;g=AAEL010667) | lethal(2)essential for life protein, l2efl [Source:VB External Description;Acc:AAEL010667] | 2.968056 | 2.360656 | 3.833695 | 5.171847 |
| [AAEL010668](https://www.vectorbase.org/aedes_aegypti/Gene/Summary?db=core;g=AAEL010668) | quinone oxidoreductase [Source:VB External Description;Acc:AAEL010668] | 0.047445 | -0.13468 | 0.106179 | 1.202761 |
| [AAEL010670](https://www.vectorbase.org/aedes_aegypti/Gene/Summary?db=core;g=AAEL010670) | lethal(2)essential for life protein, l2efl [Source:VB External Description;Acc:AAEL010670] | 1.211831 | 0.258623 | 0.636642 | 1.891161 |
| [AAEL010675](https://www.vectorbase.org/aedes_aegypti/Gene/Summary?db=core;g=AAEL010675) |  | 0.112207 | 0.071686 | 0.123403 | 1.198674 |
| [AAEL010695](https://www.vectorbase.org/aedes_aegypti/Gene/Summary?db=core;g=AAEL010695) |  | 0.43203 | 0.880808 | 0.605127 | 0.764342 |
| [AAEL010724](https://www.vectorbase.org/aedes_aegypti/Gene/Summary?db=core;g=AAEL010724) |  | -0.19688 | 0.271316 | 0.150499 | 1.144099 |
| [AAEL010731](https://www.vectorbase.org/aedes_aegypti/Gene/Summary?db=core;g=AAEL010731) |  | 0.275472 | 0.480844 | 0.266749 | 1.263107 |
| [AAEL010751](https://www.vectorbase.org/aedes_aegypti/Gene/Summary?db=core;g=AAEL010751) | methylenetetrahydrofolate dehydrogenase [Source:VB External Description;Acc:AAEL010751] | 0.321703 | 0.423874 | 0.573886 | 1.120435 |
| [AAEL010844](https://www.vectorbase.org/aedes_aegypti/Gene/Summary?db=core;g=AAEL010844) | ubiquitin-conjugating enzyme E2 j2 [Source:VB External Description;Acc:AAEL010844] | 0.201096 | 0.49244 | 0.581016 | 0.858719 |
| [AAEL010905](https://www.vectorbase.org/aedes_aegypti/Gene/Summary?db=core;g=AAEL010905) |  | 0.00712 | 0.197666 | 0.336708 | 0.875495 |
| [AAEL010937](https://www.vectorbase.org/aedes_aegypti/Gene/Summary?db=core;g=AAEL010937) |  | 0.063632 | 0.528245 | 0.561483 | 1.294054 |
| [AAEL010939](https://www.vectorbase.org/aedes_aegypti/Gene/Summary?db=core;g=AAEL010939) | group ii plp decarboxylase [Source:VB External Description;Acc:AAEL010939] | 0.125612 | 0.311974 | 0.367489 | 0.809837 |
| [AAEL010945](https://www.vectorbase.org/aedes_aegypti/Gene/Summary?db=core;g=AAEL010945) |  | 0.045012 | 0.366495 | 0.308176 | 0.785324 |
| [AAEL010996](https://www.vectorbase.org/aedes_aegypti/Gene/Summary?db=core;g=AAEL010996) |  | 0.378756 | 0.546787 | 0.550587 | 1.060154 |
| [AAEL011017](https://www.vectorbase.org/aedes_aegypti/Gene/Summary?db=core;g=AAEL011017) |  | -0.07561 | 0.153727 | 0.276484 | 0.774334 |
| [AAEL011038](https://www.vectorbase.org/aedes_aegypti/Gene/Summary?db=core;g=AAEL011038) | integrin alpha-ps [Source:VB External Description;Acc:AAEL011038] | 0.122767 | 0.731315 | 0.950434 | 1.534926 |
| [AAEL011081](https://www.vectorbase.org/aedes_aegypti/Gene/Summary?db=core;g=AAEL011081) |  | 0.164425 | 0.290068 | 0.357516 | 0.664722 |
| [AAEL011116](https://www.vectorbase.org/aedes_aegypti/Gene/Summary?db=core;g=AAEL011116) | 14-3-3 protein sigma, gamma, zeta, beta/alpha [Source:VB External Description;Acc:AAEL011116] | 0.089976 | 0.567088 | 0.406074 | 0.730327 |
| [AAEL011151](https://www.vectorbase.org/aedes_aegypti/Gene/Summary?db=core;g=AAEL011151) |  | 0.613415 | 0.365896 | 0.44253 | 1.936736 |
| [AAEL011153](https://www.vectorbase.org/aedes_aegypti/Gene/Summary?db=core;g=AAEL011153) |  | 0.267051 | 0.765293 | 0.599528 | 1.674835 |
| [AAEL011154](https://www.vectorbase.org/aedes_aegypti/Gene/Summary?db=core;g=AAEL011154) |  | 0.058528 | 0.442062 | 0.55046 | 1.466879 |
| [AAEL011168](https://www.vectorbase.org/aedes_aegypti/Gene/Summary?db=core;g=AAEL011168) | GTP-binding protein (i) alpha subunit, gnai [Source:VB External Description;Acc:AAEL011168] | 0.212863 | 0.617847 | 0.932631 | 1.272986 |
| [AAEL011180](https://www.vectorbase.org/aedes_aegypti/Gene/Summary?db=core;g=AAEL011180) |  | -0.14237 | -0.01196 | 0.38296 | 0.679427 |
| [AAEL011197](https://www.vectorbase.org/aedes_aegypti/Gene/Summary?db=core;g=AAEL011197) | actin [Source:VB External Description;Acc:AAEL011197] | -0.05431 | 0.251848 | 0.402287 | 0.842895 |
| [AAEL011203](https://www.vectorbase.org/aedes_aegypti/Gene/Summary?db=core;g=AAEL011203) |  | 0.043522 | 0.481636 | 0.657786 | 0.955669 |
| [AAEL011216](https://www.vectorbase.org/aedes_aegypti/Gene/Summary?db=core;g=AAEL011216) |  | 0.302826 | 0.686039 | 0.480679 | 0.764456 |
| [AAEL011242](https://www.vectorbase.org/aedes_aegypti/Gene/Summary?db=core;g=AAEL011242) | udp-glucose 6-dehydrogenase [Source:VB External Description;Acc:AAEL011242] | 0.264511 | 0.248706 | 0.794184 | 1.258778 |
| [AAEL011245](https://www.vectorbase.org/aedes_aegypti/Gene/Summary?db=core;g=AAEL011245) | deoxyhypusine synthase [Source:VB External Description;Acc:AAEL011245] | 0.209067 | 0.555052 | 0.357379 | 0.869227 |
| [AAEL011250](https://www.vectorbase.org/aedes_aegypti/Gene/Summary?db=core;g=AAEL011250) |  | -0.03359 | 0.430265 | 0.780733 | 1.440647 |
| [AAEL011271](https://www.vectorbase.org/aedes_aegypti/Gene/Summary?db=core;g=AAEL011271) | programmed cell death 6-interacting protein [Source:VB External Description;Acc:AAEL011271] | 0.224314 | 0.421961 | 0.373544 | 0.812519 |
| [AAEL011274](https://www.vectorbase.org/aedes_aegypti/Gene/Summary?db=core;g=AAEL011274) | 1-phosphatidylinositol-4-phosphate 5-kinase, putative [Source:VB External Description;Acc:AAEL011274] | 0.22166 | 0.446782 | 0.356783 | 0.639459 |
| [AAEL011302](https://www.vectorbase.org/aedes_aegypti/Gene/Summary?db=core;g=AAEL011302) | annexin [Source:VB External Description;Acc:AAEL011302] | -0.28021 | -0.11053 | 0.33179 | 0.968835 |
| [AAEL011307](https://www.vectorbase.org/aedes_aegypti/Gene/Summary?db=core;g=AAEL011307) | RAD51C protein, putative [Source:VB External Description;Acc:AAEL011307] | 0.262625 | 0.666612 | 0.752895 | 1.241605 |
| [AAEL011316](https://www.vectorbase.org/aedes_aegypti/Gene/Summary?db=core;g=AAEL011316) | zinc finger protein [Source:VB External Description;Acc:AAEL011316] | -0.22885 | 0.196474 | 0.668864 | 1.219236 |
| [AAEL011371](https://www.vectorbase.org/aedes_aegypti/Gene/Summary?db=core;g=AAEL011371) |  | 1.044582 | 1.196346 | 1.4931 | 1.979347 |
| [AAEL011441](https://www.vectorbase.org/aedes_aegypti/Gene/Summary?db=core;g=AAEL011441) | calcium/calmodulin-dependent protein kinase type 1 (camki) [Source:VB External Description;Acc:AAEL011441] | 0.295581 | 0.414551 | 0.471291 | 0.689216 |
| [AAEL011525](https://www.vectorbase.org/aedes_aegypti/Gene/Summary?db=core;g=AAEL011525) |  | 0.688408 | 0.952901 | 1.209857 | 1.606998 |
| [AAEL011527](https://www.vectorbase.org/aedes_aegypti/Gene/Summary?db=core;g=AAEL011527) | eukaryotic translation initiation factor [Source:VB External Description;Acc:AAEL011527] | 0.073199 | 0.417015 | 0.326753 | 0.714361 |
| [AAEL011536](https://www.vectorbase.org/aedes_aegypti/Gene/Summary?db=core;g=AAEL011536) | phosphoglucomutase [Source:VB External Description;Acc:AAEL011536] | -0.10352 | 0.458686 | 0.703982 | 1.143444 |
| [AAEL011551](https://www.vectorbase.org/aedes_aegypti/Gene/Summary?db=core;g=AAEL011551) |  | -0.11846 | -0.07175 | 0.10308 | 0.775383 |
| [AAEL011564](https://www.vectorbase.org/aedes_aegypti/Gene/Summary?db=core;g=AAEL011564) | choline-phosphate cytidylyltransferase a, b [Source:VB External Description;Acc:AAEL011564] | 0.222673 | 0.676384 | 1.365257 | 1.817696 |
| [AAEL011641](https://www.vectorbase.org/aedes_aegypti/Gene/Summary?db=core;g=AAEL011641) | transferrin [Source:VB External Description;Acc:AAEL011641] | -0.07147 | 0.042019 | 0.343668 | 1.032318 |
| [AAEL011648](https://www.vectorbase.org/aedes_aegypti/Gene/Summary?db=core;g=AAEL011648) | cyclin d [Source:VB External Description;Acc:AAEL011648] | 0.620851 | 1.08969 | 0.369061 | 1.756443 |
| [AAEL011704](https://www.vectorbase.org/aedes_aegypti/Gene/Summary?db=core;g=AAEL011704) | heat shock protein [Source:VB External Description;Acc:AAEL011704] | 0.288731 | 0.581706 | 0.383502 | 0.910466 |
| [AAEL011773](https://www.vectorbase.org/aedes_aegypti/Gene/Summary?db=core;g=AAEL011773) | calreticulin [Source:VB External Description;Acc:AAEL011773] | 0.074835 | 0.595557 | 0.473587 | 1.048358 |
| [AAEL011778](https://www.vectorbase.org/aedes_aegypti/Gene/Summary?db=core;g=AAEL011778) | WD-repeat protein [Source:VB External Description;Acc:AAEL011778] | -0.08077 | 0.276129 | 0.531625 | 1.272486 |
| [AAEL011821](https://www.vectorbase.org/aedes_aegypti/Gene/Summary?db=core;g=AAEL011821) | adenylyl cyclase-associated protein [Source:VB External Description;Acc:AAEL011821] | 0.383351 | 0.809697 | 0.636239 | 1.260619 |
| [AAEL011867](https://www.vectorbase.org/aedes_aegypti/Gene/Summary?db=core;g=AAEL011867) |  | -0.45247 | -0.13921 | 0.373315 | 1.078256 |
| [AAEL011912](https://www.vectorbase.org/aedes_aegypti/Gene/Summary?db=core;g=AAEL011912) | mannose-1-phosphate guanyltransferase [Source:VB External Description;Acc:AAEL011912] | -0.17016 | -0.19151 | 0.08034 | 0.648622 |
| [AAEL011953](https://www.vectorbase.org/aedes_aegypti/Gene/Summary?db=core;g=AAEL011953) | oxysterol-binding protein related protein (ORP9) [Source:VB Community Annotation;Acc:AAEL011953] | 0.243937 | 0.364596 | 0.429682 | 0.866454 |
| [AAEL011957](https://www.vectorbase.org/aedes_aegypti/Gene/Summary?db=core;g=AAEL011957) | elongase, putative [Source:VB External Description;Acc:AAEL011957] | 1.579256 | 1.85635 | 1.797018 | 2.353284 |
| [AAEL012003](https://www.vectorbase.org/aedes_aegypti/Gene/Summary?db=core;g=AAEL012003) | galectin [Source:VB Community Annotation;Acc:AAEL012003] | 0.070524 | 0.491824 | 0.484914 | 1.425892 |
| [AAEL012011](https://www.vectorbase.org/aedes_aegypti/Gene/Summary?db=core;g=AAEL012011) | nedd8-conjugating enzyme nce2 [Source:VB External Description;Acc:AAEL012011] | 0.007801 | 0.621765 | 0.270867 | 0.82382 |
| [AAEL012014](https://www.vectorbase.org/aedes_aegypti/Gene/Summary?db=core;g=AAEL012014) | l-lactate dehydrogenase [Source:VB External Description;Acc:AAEL012014] | -0.29148 | 0.115805 | -0.01077 | 1.584012 |
| [AAEL012017](https://www.vectorbase.org/aedes_aegypti/Gene/Summary?db=core;g=AAEL012017) |  | 0.803765 | 1.31145 | 0.810835 | 2.354989 |
| [AAEL012097](https://www.vectorbase.org/aedes_aegypti/Gene/Summary?db=core;g=AAEL012097) | app binding protein [Source:VB External Description;Acc:AAEL012097] | 0.666708 | 0.849011 | 0.719092 | 1.10177 |
| [AAEL012099](https://www.vectorbase.org/aedes_aegypti/Gene/Summary?db=core;g=AAEL012099) | protease m1 zinc metalloprotease [Source:VB External Description;Acc:AAEL012099] | 0.174081 | 0.684683 | 1.136967 | 2.06512 |
| [AAEL012116](https://www.vectorbase.org/aedes_aegypti/Gene/Summary?db=core;g=AAEL012116) | glutaredoxin, putative [Source:VB External Description;Acc:AAEL012116] | -0.05522 | 0.541074 | 0.016657 | 1.180069 |
| [AAEL012184](https://www.vectorbase.org/aedes_aegypti/Gene/Summary?db=core;g=AAEL012184) | glutaminyl-peptide cyclotransferase [Source:VB External Description;Acc:AAEL012184] | -0.29531 | -0.00767 | 0.935019 | 2.297263 |
| [AAEL012240](https://www.vectorbase.org/aedes_aegypti/Gene/Summary?db=core;g=AAEL012240) | protein transport protein sec13 [Source:VB External Description;Acc:AAEL012240] | -0.17856 | -0.0259 | 0.092583 | 0.677858 |
| [AAEL012247](https://www.vectorbase.org/aedes_aegypti/Gene/Summary?db=core;g=AAEL012247) |  | 0.218444 | 0.318326 | 0.335642 | 0.689762 |
| [AAEL012279](https://www.vectorbase.org/aedes_aegypti/Gene/Summary?db=core;g=AAEL012279) | Eukaryotic translation initiation factor 3 subunit J (eIF3j) [Source:VB External Description;Acc:AAEL012279] | 0.330748 | 0.778296 | 0.582693 | 0.87158 |
| [AAEL012280](https://www.vectorbase.org/aedes_aegypti/Gene/Summary?db=core;g=AAEL012280) | pef protein with a long n-terminal hydrophobic domain (peflin) [Source:VB External Description;Acc:AAEL012280] | 0.0752 | 0.255237 | 0.695577 | 1.332723 |
| [AAEL012283](https://www.vectorbase.org/aedes_aegypti/Gene/Summary?db=core;g=AAEL012283) | diaphanous [Source:VB External Description;Acc:AAEL012283] | 0.183298 | 0.669461 | 0.371809 | 1.090778 |
| [AAEL012313](https://www.vectorbase.org/aedes_aegypti/Gene/Summary?db=core;g=AAEL012313) | charged multivesicular body protein 5 [Source:VB External Description;Acc:AAEL012313] | -0.13203 | 0.05013 | 0.168133 | 0.653774 |
| [AAEL012341](https://www.vectorbase.org/aedes_aegypti/Gene/Summary?db=core;g=AAEL012341) | lysosomal acid lipase, putative [Source:VB External Description;Acc:AAEL012341] | -0.08285 | -0.0423 | 0.520872 | 1.126758 |
| [AAEL012408](https://www.vectorbase.org/aedes_aegypti/Gene/Summary?db=core;g=AAEL012408) | mitochondrial ribosomal protein, L53, putative [Source:VB Community Annotation;Acc:AAEL012408] | 0.170582 | 0.006369 | 9.09871470321932e-05 | 1.171743 |
| [AAEL012413](https://www.vectorbase.org/aedes_aegypti/Gene/Summary?db=core;g=AAEL012413) | n-acetyltransferase mak3 [Source:VB External Description;Acc:AAEL012413] | 0.095416 | 0.441785 | 0.375635 | 0.808453 |
| [AAEL012419](https://www.vectorbase.org/aedes_aegypti/Gene/Summary?db=core;g=AAEL012419) | 26S proteasome subunit S9 [Source:VB External Description;Acc:AAEL012419] | 0.102371 | 0.4282 | 0.081821 | 0.745808 |
| [AAEL012425](https://www.vectorbase.org/aedes_aegypti/Gene/Summary?db=core;g=AAEL012425) | sterol desaturase [Source:VB External Description;Acc:AAEL012425] | -0.27641 | -0.19829 | 0.881924 | 1.748532 |
| [AAEL012466](https://www.vectorbase.org/aedes_aegypti/Gene/Summary?db=core;g=AAEL012466) | integrin beta subunit [Source:VB External Description;Acc:AAEL012466] | 0.004249 | 0.211239 | 0.211763 | 0.90061 |
| [AAEL012471](https://www.vectorbase.org/aedes_aegypti/Gene/Summary?db=core;g=AAEL012471) | JAKSTAT pathway signalling Transmembrane Receptor Domeless. [Source:VB Community Annotation;Acc:AAEL012471] | 0.088533 | 0.36014 | 0.961318 | 1.98936 |
| [AAEL012512](https://www.vectorbase.org/aedes_aegypti/Gene/Summary?db=core;g=AAEL012512) | Inhibitor of Apoptosis (IAP) containing Baculoviral IAP Repeat(s) (BIR domains). [Source:VB Community Annotation;Acc:AAEL012512] | 0.295999 | 0.211276 | 1.458135 | 2.816818 |
| [AAEL012515](https://www.vectorbase.org/aedes_aegypti/Gene/Summary?db=core;g=AAEL012515) | tumor suppressor protein, putative [Source:VB External Description;Acc:AAEL012515] | -0.13096 | 0.025925 | 0.162606 | 0.737112 |
| [AAEL012546](https://www.vectorbase.org/aedes_aegypti/Gene/Summary?db=core;g=AAEL012546) | DNA replication licensing factor MCM6 [Source:VB External Description;Acc:AAEL012546] | 0.057911 | 0.417432 | -0.13125 | 0.876017 |
| [AAEL012554](https://www.vectorbase.org/aedes_aegypti/Gene/Summary?db=core;g=AAEL012554) | maltose phosphorylase [Source:VB External Description;Acc:AAEL012554] | 0.273264 | 0.765347 | 0.795433 | 1.72599 |
| [AAEL012600](https://www.vectorbase.org/aedes_aegypti/Gene/Summary?db=core;g=AAEL012600) |  | 0.023072 | 0.270731 | 0.500742 | 0.834518 |
| [AAEL012602](https://www.vectorbase.org/aedes_aegypti/Gene/Summary?db=core;g=AAEL012602) |  | 0.242607 | 0.415945 | 0.535694 | 1.402656 |
| [AAEL012611](https://www.vectorbase.org/aedes_aegypti/Gene/Summary?db=core;g=AAEL012611) |  | 0.091869 | 0.111484 | 0.28657 | 0.589736 |
| [AAEL012619](https://www.vectorbase.org/aedes_aegypti/Gene/Summary?db=core;g=AAEL012619) |  | 0.140414 | 0.726104 | 0.838609 | 1.509926 |
| [AAEL012702](https://www.vectorbase.org/aedes_aegypti/Gene/Summary?db=core;g=AAEL012702) | ATP-binding cassette sub-family A member 3, putative [Source:VB External Description;Acc:AAEL012702] | 0.117997 | 0.396104 | 0.773 | 1.268627 |
| [AAEL012784](https://www.vectorbase.org/aedes_aegypti/Gene/Summary?db=core;g=AAEL012784) |  | 0.04623 | -0.01168 | 0.16326 | 0.836156 |
| [AAEL012790](https://www.vectorbase.org/aedes_aegypti/Gene/Summary?db=core;g=AAEL012790) | lipase [Source:VB External Description;Acc:AAEL012790] | 0.141154 | 1.242455 | 1.121168 | 2.472693 |
| [AAEL012827](https://www.vectorbase.org/aedes_aegypti/Gene/Summary?db=core;g=AAEL012827) | endoplasmin [Source:VB External Description;Acc:AAEL012827] | 0.091237 | 0.652152 | 0.446413 | 0.798398 |
| [AAEL012835](https://www.vectorbase.org/aedes_aegypti/Gene/Summary?db=core;g=AAEL012835) | 85 kda calcium-independent phospholipase A2 (ipla2) [Source:VB External Description;Acc:AAEL012835] | 0.024423 | 0.079252 | 0.473542 | 0.784133 |
| [AAEL012856](https://www.vectorbase.org/aedes_aegypti/Gene/Summary?db=core;g=AAEL012856) |  | 0.108188 | -0.47326 | 0.837698 | 1.429791 |
| [AAEL012911](https://www.vectorbase.org/aedes_aegypti/Gene/Summary?db=core;g=AAEL012911) | leucine-rich immune protein (Coil-less) [Source:VB Community Annotation;Acc:AAEL012911] | 0.264495 | 0.602295 | 0.83828 | 1.387499 |
| [AAEL012939](https://www.vectorbase.org/aedes_aegypti/Gene/Summary?db=core;g=AAEL012939) | gamma-subunit,methylmalonyl-CoA decarboxylase, putative [Source:VB External Description;Acc:AAEL012939] | -0.05958 | 0.594522 | 0.71197 | 1.366693 |
| [AAEL012949](https://www.vectorbase.org/aedes_aegypti/Gene/Summary?db=core;g=AAEL012949) | transferrin [Source:VB External Description;Acc:AAEL012949] | 0.336818 | -0.34371 | 0.04532 | 1.931416 |
| [AAEL012978](https://www.vectorbase.org/aedes_aegypti/Gene/Summary?db=core;g=AAEL012978) |  | 0.038811 | -0.07667 | 0.430108 | 0.629339 |
| [AAEL013005](https://www.vectorbase.org/aedes_aegypti/Gene/Summary?db=core;g=AAEL013005) |  | -0.12907 | 0.341271 | 0.221462 | 0.849004 |
| [AAEL013047](https://www.vectorbase.org/aedes_aegypti/Gene/Summary?db=core;g=AAEL013047) | sphingolipid delta 4 desaturase/c-4 hydroxylase protein des2 [Source:VB External Description;Acc:AAEL013047] | 0.091891 | 0.499287 | 0.683226 | 1.193767 |
| [AAEL013065](https://www.vectorbase.org/aedes_aegypti/Gene/Summary?db=core;g=AAEL013065) | thiamin pyrophosphokinase [Source:VB External Description;Acc:AAEL013065] | 0.23593 | 0.598726 | 0.839355 | 1.1606 |
| [AAEL013074](https://www.vectorbase.org/aedes_aegypti/Gene/Summary?db=core;g=AAEL013074) | adenylyl cyclase-associated protein [Source:VB External Description;Acc:AAEL013074] | 0.003003 | 0.160256 | 0.408421 | 0.930026 |
| [AAEL013093](https://www.vectorbase.org/aedes_aegypti/Gene/Summary?db=core;g=AAEL013093) | valacyclovir hydrolase [Source:VB External Description;Acc:AAEL013093] | 0.219925 | 0.485917 | 0.58988 | 1.264866 |
| [AAEL013114](https://www.vectorbase.org/aedes_aegypti/Gene/Summary?db=core;g=AAEL013114) | DNA-J, putative [Source:VB External Description;Acc:AAEL013114] | 0.006115 | 0.455688 | 0.28198 | 0.919942 |
| [AAEL013119](https://www.vectorbase.org/aedes_aegypti/Gene/Summary?db=core;g=AAEL013119) | charged multivesicular body protein [Source:VB External Description;Acc:AAEL013119] | -0.0109 | 0.270928 | 0.160464 | 0.70943 |
| [AAEL013128](https://www.vectorbase.org/aedes_aegypti/Gene/Summary?db=core;g=AAEL013128) | elongase, putative [Source:VB External Description;Acc:AAEL013128] | 0.339132 | 0.672233 | 1.139023 | 1.159622 |
| [AAEL013197](https://www.vectorbase.org/aedes_aegypti/Gene/Summary?db=core;g=AAEL013197) |  | 0.192057 | -0.16026 | 0.563369 | 1.937503 |
| [AAEL013208](https://www.vectorbase.org/aedes_aegypti/Gene/Summary?db=core;g=AAEL013208) |  | 0.031088 | 0.24508 | 0.370829 | 0.785215 |
| [AAEL013220](https://www.vectorbase.org/aedes_aegypti/Gene/Summary?db=core;g=AAEL013220) |  | -0.02647 | 0.153885 | -0.00689 | 0.674227 |
| [AAEL013229](https://www.vectorbase.org/aedes_aegypti/Gene/Summary?db=core;g=AAEL013229) | tubulin alpha chain [Source:VB External Description;Acc:AAEL013229] | -0.00581 | 0.34294 | 0.688628 | 1.523921 |
| [AAEL013230](https://www.vectorbase.org/aedes_aegypti/Gene/Summary?db=core;g=AAEL013230) | coatomer delta subunit [Source:VB External Description;Acc:AAEL013230] | 0.011086 | 0.225727 | 0.27712 | 0.651598 |
| [AAEL013252](https://www.vectorbase.org/aedes_aegypti/Gene/Summary?db=core;g=AAEL013252) |  | 0.267749 | 0.637235 | 0.49256 | 0.869094 |
| [AAEL013262](https://www.vectorbase.org/aedes_aegypti/Gene/Summary?db=core;g=AAEL013262) |  | -0.07939 | 0.038614 | 0.220126 | 1.460294 |
| [AAEL013307](https://www.vectorbase.org/aedes_aegypti/Gene/Summary?db=core;g=AAEL013307) |  | 0.205876 | 0.804188 | 0.517112 | 1.10972 |
| [AAEL013314](https://www.vectorbase.org/aedes_aegypti/Gene/Summary?db=core;g=AAEL013314) | calicylin binding protein [Source:VB External Description;Acc:AAEL013314] | 0.241182 | 0.604457 | 0.37359 | 0.707448 |
| [AAEL013341](https://www.vectorbase.org/aedes_aegypti/Gene/Summary?db=core;g=AAEL013341) | lethal(2)essential for life protein, l2efl [Source:VB External Description;Acc:AAEL013341] | 0.156775 | 0.248524 | 0.748064 | 1.053099 |
| [AAEL013344](https://www.vectorbase.org/aedes_aegypti/Gene/Summary?db=core;g=AAEL013344) | lethal(2)essential for life protein, l2efl [Source:VB External Description;Acc:AAEL013344] | 1.04229 | 0.512564 | 1.317792 | 1.767601 |
| [AAEL013346](https://www.vectorbase.org/aedes_aegypti/Gene/Summary?db=core;g=AAEL013346) | lethal(2)essential for life protein, l2efl [Source:VB External Description;Acc:AAEL013346] | 0.410351 | 1.273891 | 1.226997 | 2.319326 |
| [AAEL013347](https://www.vectorbase.org/aedes_aegypti/Gene/Summary?db=core;g=AAEL013347) | lethal(2)essential for life protein, l2efl [Source:VB External Description;Acc:AAEL013347] | 0.579665 | 0.50992 | 1.28555 | 2.432112 |
| [AAEL013348](https://www.vectorbase.org/aedes_aegypti/Gene/Summary?db=core;g=AAEL013348) | lethal(2)essential for life protein, l2efl [Source:VB External Description;Acc:AAEL013348] | 0.45444 | 0.703502 | 2.33658 | 3.752691 |
| [AAEL013352](https://www.vectorbase.org/aedes_aegypti/Gene/Summary?db=core;g=AAEL013352) | lethal(2)essential for life protein, l2efl [Source:VB External Description;Acc:AAEL013352] | 0.355976 | 0.581981 | 1.374949 | 3.03239 |
| [AAEL013381](https://www.vectorbase.org/aedes_aegypti/Gene/Summary?db=core;g=AAEL013381) | integrin alpha-ps [Source:VB External Description;Acc:AAEL013381] | 0.052601 | 0.392126 | 0.7884 | 1.0664 |
| [AAEL013525](https://www.vectorbase.org/aedes_aegypti/Gene/Summary?db=core;g=AAEL013525) | Timp-3, putative [Source:VB External Description;Acc:AAEL013525] | 0.31721 | 0.099692 | 0.487573 | 1.034988 |
| [AAEL013581](https://www.vectorbase.org/aedes_aegypti/Gene/Summary?db=core;g=AAEL013581) | valacyclovir hydrolase [Source:VB External Description;Acc:AAEL013581] | 0.084027 | 0.526908 | 1.188519 | 1.109611 |
| [AAEL013629](https://www.vectorbase.org/aedes_aegypti/Gene/Summary?db=core;g=AAEL013629) | trypsin-alpha, putative [Source:VB External Description;Acc:AAEL013629] | 0.609388 | 1.278028 | 0.82771 | 1.601766 |
| [AAEL013661](https://www.vectorbase.org/aedes_aegypti/Gene/Summary?db=core;g=AAEL013661) | actin binding protein, putative [Source:VB External Description;Acc:AAEL013661] | 0.509048 | 0.952866 | 0.584499 | 1.069093 |
| [AAEL013679](https://www.vectorbase.org/aedes_aegypti/Gene/Summary?db=core;g=AAEL013679) | coilin-interacting nulcear ATPase protein, putative [Source:VB External Description;Acc:AAEL013679] | 0.06883 | 0.302361 | 0.570082 | 0.901345 |
| [AAEL013710](https://www.vectorbase.org/aedes_aegypti/Gene/Summary?db=core;g=AAEL013710) |  | 0.040426 | 0.077118 | 0.213192 | 0.639287 |
| [AAEL013780](https://www.vectorbase.org/aedes_aegypti/Gene/Summary?db=core;g=AAEL013780) |  | -0.14452 | 0.046779 | 0.095362 | 0.491706 |
| [AAEL013786](https://www.vectorbase.org/aedes_aegypti/Gene/Summary?db=core;g=AAEL013786) | growth factor receptor-bound protein [Source:VB External Description;Acc:AAEL013786] | 0.305162 | 0.924467 | 0.633552 | 1.075827 |
| [AAEL013812](https://www.vectorbase.org/aedes_aegypti/Gene/Summary?db=core;g=AAEL013812) |  | 0.692089 | 0.631445 | 0.949898 | 1.658824 |
| [AAEL013875](https://www.vectorbase.org/aedes_aegypti/Gene/Summary?db=core;g=AAEL013875) | tetraspanin, putative [Source:VB External Description;Acc:AAEL013875] | 0.064435 | 0.380745 | 0.305391 | 0.743953 |
| [AAEL013996](https://www.vectorbase.org/aedes_aegypti/Gene/Summary?db=core;g=AAEL013996) |  | -0.2158 | 0.428 | 0.422041 | 1.091704 |
| [AAEL014003](https://www.vectorbase.org/aedes_aegypti/Gene/Summary?db=core;g=AAEL014003) |  | 0.346773 | 0.667145 | 0.996193 | 1.693605 |
| [AAEL014018](https://www.vectorbase.org/aedes_aegypti/Gene/Summary?db=core;g=AAEL014018) |  | -0.03752 | 0.25891 | 0.582217 | 1.201761 |
| [AAEL014022](https://www.vectorbase.org/aedes_aegypti/Gene/Summary?db=core;g=AAEL014022) |  | 0.084723 | 0.44595 | 0.830416 | 1.246482 |
| [AAEL014035](https://www.vectorbase.org/aedes_aegypti/Gene/Summary?db=core;g=AAEL014035) | suppressor of actin (sac) [Source:VB External Description;Acc:AAEL014035] | 0.091561 | 0.465609 | 0.696557 | 1.190309 |
| [AAEL014064](https://www.vectorbase.org/aedes_aegypti/Gene/Summary?db=core;g=AAEL014064) | glutaredoxin, putative [Source:VB External Description;Acc:AAEL014064] | -0.09177 | 0.000673 | 0.244624 | 0.73549 |
| [AAEL014111](https://www.vectorbase.org/aedes_aegypti/Gene/Summary?db=core;g=AAEL014111) | tubulin-specific chaperone, putative [Source:VB External Description;Acc:AAEL014111] | 0.139157 | 0.32915 | 0.596492 | 0.792191 |
| [AAEL014158](https://www.vectorbase.org/aedes_aegypti/Gene/Summary?db=core;g=AAEL014158) |  | 0.102614 | 0.29411 | 0.373981 | 0.799419 |
| [AAEL014160](https://www.vectorbase.org/aedes_aegypti/Gene/Summary?db=core;g=AAEL014160) |  | -0.04123 | 0.175629 | 0.440692 | 0.842321 |
| [AAEL014204](https://www.vectorbase.org/aedes_aegypti/Gene/Summary?db=core;g=AAEL014204) | 6-phosphogluconolactonase [Source:VB External Description;Acc:AAEL014204] | -0.03996 | -0.01214 | 0.264938 | 0.748551 |
| [AAEL014268](https://www.vectorbase.org/aedes_aegypti/Gene/Summary?db=core;g=AAEL014268) |  | 0.038253 | 0.243613 | 0.285237 | 0.96552 |
| [AAEL014310](https://www.vectorbase.org/aedes_aegypti/Gene/Summary?db=core;g=AAEL014310) |  | 0.018367 | 0.227985 | 0.313052 | 0.660858 |
| [AAEL014319](https://www.vectorbase.org/aedes_aegypti/Gene/Summary?db=core;g=AAEL014319) |  | 0.416048 | 1.105112 | 0.919841 | 1.715621 |
| [AAEL014344](https://www.vectorbase.org/aedes_aegypti/Gene/Summary?db=core;g=AAEL014344) | adam (a disintegrin and metalloprotease) [Source:VB External Description;Acc:AAEL014344] | -0.35092 | 0.05522 | 0.323781 | 1.269863 |
| [AAEL014348](https://www.vectorbase.org/aedes_aegypti/Gene/Summary?db=core;g=AAEL014348) | caspase (short) [Source:VB Community Annotation;Acc:AAEL014348] | 0.370355 | 0.52055 | 0.569325 | 1.592769 |
| [AAEL014355](https://www.vectorbase.org/aedes_aegypti/Gene/Summary?db=core;g=AAEL014355) | symbol, putative [Source:VB External Description;Acc:AAEL014355] | 0.493828 | 0.820363 | 0.749553 | 0.892081 |
| [AAEL014375](https://www.vectorbase.org/aedes_aegypti/Gene/Summary?db=core;g=AAEL014375) | clathrin coat assembly protein ap17 [Source:VB External Description;Acc:AAEL014375] | 0.078897 | 0.297216 | 0.190092 | 0.661224 |
| [AAEL014539](https://www.vectorbase.org/aedes_aegypti/Gene/Summary?db=core;g=AAEL014539) | maltose phosphorylase [Source:VB External Description;Acc:AAEL014539] | -0.36582 | 0.129295 | 0.932347 | 1.343031 |
| [AAEL014541](https://www.vectorbase.org/aedes_aegypti/Gene/Summary?db=core;g=AAEL014541) | maltose phosphorylase [Source:VB External Description;Acc:AAEL014541] | -0.31088 | -0.15151 | 0.328318 | 0.997395 |
| [AAEL014549](https://www.vectorbase.org/aedes_aegypti/Gene/Summary?db=core;g=AAEL014549) |  | -0.06199 | -0.06264 | 0.18783 | 0.660614 |
| [AAEL014564](https://www.vectorbase.org/aedes_aegypti/Gene/Summary?db=core;g=AAEL014564) |  | 0.299275 | 0.576353 | 0.426635 | 0.979008 |
| [AAEL014598](https://www.vectorbase.org/aedes_aegypti/Gene/Summary?db=core;g=AAEL014598) | huntingtin interacting protein [Source:VB External Description;Acc:AAEL014598] | -0.03846 | 0.34064 | 0.757953 | 1.033291 |
| [AAEL014722](https://www.vectorbase.org/aedes_aegypti/Gene/Summary?db=core;g=AAEL014722) | coilin-interacting nulcear ATPase protein, putative [Source:VB External Description;Acc:AAEL014722] | -0.19838 | 0.394295 | 0.607689 | 1.368671 |
| [AAEL014749](https://www.vectorbase.org/aedes_aegypti/Gene/Summary?db=core;g=AAEL014749) | ral [Source:VB External Description;Acc:AAEL014749] | -0.05267 | 0.492609 | 0.515808 | 1.169643 |
| [AAEL014763](https://www.vectorbase.org/aedes_aegypti/Gene/Summary?db=core;g=AAEL014763) | tak1 binding protein-1 [Source:VB External Description;Acc:AAEL014763] | -0.093 | -0.1238 | -0.00941 | 0.655253 |
| [AAEL014847](https://www.vectorbase.org/aedes_aegypti/Gene/Summary?db=core;g=AAEL014847) | innexin [Source:VB Community Annotation;Acc:AAEL014847] | 0.482591 | 0.852902 | 0.571312 | 0.96528 |
| [AAEL014931](https://www.vectorbase.org/aedes_aegypti/Gene/Summary?db=core;g=AAEL014931) | sarm1 [Source:VB External Description;Acc:AAEL014931] | 0.358965 | 0.285732 | 0.487124 | 0.900104 |
| [AAEL014996](https://www.vectorbase.org/aedes_aegypti/Gene/Summary?db=core;g=AAEL014996) | mitochondrial ribosomal protein, L53, putative [Source:VB Community Annotation;Acc:AAEL014996] | 0.010959 | 0.111957 | 0.004845 | 1.087038 |
| [AAEL015001](https://www.vectorbase.org/aedes_aegypti/Gene/Summary?db=core;g=AAEL015001) | coatomer [Source:VB External Description;Acc:AAEL015001] | 0.054215 | 0.303154 | 0.192538 | 0.721902 |
| [AAEL015010](https://www.vectorbase.org/aedes_aegypti/Gene/Summary?db=core;g=AAEL015010) | acetyl-coa synthetase [Source:VB External Description;Acc:AAEL015010] | -0.10947 | 0.308245 | 0.383982 | 1.162749 |
| [AAEL015090](https://www.vectorbase.org/aedes_aegypti/Gene/Summary?db=core;g=AAEL015090) | heat shock protein [Source:VB External Description;Acc:AAEL015090] | 0.025406 | 0.063915 | 0.328853 | 0.842518 |
| [AAEL015100](https://www.vectorbase.org/aedes_aegypti/Gene/Summary?db=core;g=AAEL015100) | calnexin [Source:VB External Description;Acc:AAEL015100] | 0.131717 | 0.524622 | 0.473858 | 0.908021 |
| [AAEL015379](https://www.vectorbase.org/aedes_aegypti/Gene/Summary?db=core;g=AAEL015379) |  | -0.08122 | 0.199024 | 0.601192 | 1.384603 |
| [AAEL015384](https://www.vectorbase.org/aedes_aegypti/Gene/Summary?db=core;g=AAEL015384) | grb2-associated binder, gab [Source:VB External Description;Acc:AAEL015384] | 0.281555 | 0.387534 | 1.38401 | 1.737513 |
| [AAEL015493](https://www.vectorbase.org/aedes_aegypti/Gene/Summary?db=core;g=AAEL015493) |  | 0.143058 | 0.696359 | 0.694884 | 1.427197 |
| [AAEL015584](https://www.vectorbase.org/aedes_aegypti/Gene/Summary?db=core;g=AAEL015584) | molybdopterin synthase catalytic subunit 2 [Source:VB External Description;Acc:AAEL015584] | 0.039638 | 0.492855 | 0.674745 | 1.198909 |
| [AAEL015595](https://www.vectorbase.org/aedes_aegypti/Gene/Summary?db=core;g=AAEL015595) |  | -0.40762 | -0.19953 | 0.361586 | 0.841736 |
| [AAEL016975](https://www.vectorbase.org/aedes_aegypti/Gene/Summary?db=core;g=AAEL016975) |  | 0.044277 | 0.158641 | 1.183927 | 2.062773 |
| [AAEL016988](https://www.vectorbase.org/aedes_aegypti/Gene/Summary?db=core;g=AAEL016988) |  | 0.039399 | 0.402414 | 0.926005 | 1.43771 |
| [AAEL017049](https://www.vectorbase.org/aedes_aegypti/Gene/Summary?db=core;g=AAEL017049) |  | 0.394731 | 0.826572 | 0.769871 | 1.265568 |
| [AAEL017098](https://www.vectorbase.org/aedes_aegypti/Gene/Summary?db=core;g=AAEL017098) |  | -0.05729 | 0.106805 | 0.466348 | 2.267013 |
| [AAEL017121](https://www.vectorbase.org/aedes_aegypti/Gene/Summary?db=core;g=AAEL017121) |  | 0.36831 | 0.958101 | 0.91072 | 1.393873 |
| [AAEL017127](https://www.vectorbase.org/aedes_aegypti/Gene/Summary?db=core;g=AAEL017127) |  | -0.11081 | 0.472284 | 1.350507 | 1.812324 |
| [AAEL017139](https://www.vectorbase.org/aedes_aegypti/Gene/Summary?db=core;g=AAEL017139) |  | -1.17789 | 0.171159 | -0.0479 | 2.236254 |
| [AAEL017223](https://www.vectorbase.org/aedes_aegypti/Gene/Summary?db=core;g=AAEL017223) |  | -0.08819 | -0.01187 | 0.04099 | 0.671729 |
| [AAEL017248](https://www.vectorbase.org/aedes_aegypti/Gene/Summary?db=core;g=AAEL017248) |  | 0.766204 | 1.227534 | 1.358807 | 1.690352 |
| [AAEL017275](https://www.vectorbase.org/aedes_aegypti/Gene/Summary?db=core;g=AAEL017275) |  | 0.079714 | 0.309037 | 0.246106 | 0.920697 |
| [AAEL017322](https://www.vectorbase.org/aedes_aegypti/Gene/Summary?db=core;g=AAEL017322) |  | 0.343999 | 0.720549 | 0.647801 | 1.178778 |
| [AAEL017332](https://www.vectorbase.org/aedes_aegypti/Gene/Summary?db=core;g=AAEL017332) |  | 0.22678 | 0.351806 | 0.609542 | 0.864397 |
| [AAEL017339](https://www.vectorbase.org/aedes_aegypti/Gene/Summary?db=core;g=AAEL017339) |  | 0.031234 | 0.34773 | 0.694711 | 1.048986 |
| [AAEL017417](https://www.vectorbase.org/aedes_aegypti/Gene/Summary?db=core;g=AAEL017417) |  | 0.260375 | 0.754603 | 0.78817 | 1.366791 |
| [AAEL017445](https://www.vectorbase.org/aedes_aegypti/Gene/Summary?db=core;g=AAEL017445) |  | -0.01616 | 0.61187 | 1.367301 | 1.524563 |
| [AAEL017462](https://www.vectorbase.org/aedes_aegypti/Gene/Summary?db=core;g=AAEL017462) |  | 0.294406 | 0.788555 | 0.532227 | 0.906173 |
| [AAEL017483](https://www.vectorbase.org/aedes_aegypti/Gene/Summary?db=core;g=AAEL017483) |  | 0.186821 | 0.510465 | 0.449888 | 0.785352 |
| [AAEL017519](https://www.vectorbase.org/aedes_aegypti/Gene/Summary?db=core;g=AAEL017519) |  | -0.2159 | -0.01314 | 0.257838 | 0.702403 |
| [AAEL017545](https://www.vectorbase.org/aedes_aegypti/Gene/Summary?db=core;g=AAEL017545) |  | 0.452921 | 0.830992 | 0.521085 | 0.744482 |
| [AAEL017552](https://www.vectorbase.org/aedes_aegypti/Gene/Summary?db=core;g=AAEL017552) |  | 0.189686 | 0.612343 | 0.63243 | 1.122923 |
| [AAEL018061](https://www.vectorbase.org/aedes_aegypti/Gene/Summary?db=core;g=AAEL018061) |  | 0.220122 | 0.944353 | 0.61641 | 1.04381 |
| [AAEL018117](https://www.vectorbase.org/aedes_aegypti/Gene/Summary?db=core;g=AAEL018117) |  | 0.077728 | 0.489459 | 1.061021 | 1.578403 |
| [AAEL018174](https://www.vectorbase.org/aedes_aegypti/Gene/Summary?db=core;g=AAEL018174) |  | 0.132449 | 1.009986 | 0.652684 | 1.006248 |
| [AAEL018205](https://www.vectorbase.org/aedes_aegypti/Gene/Summary?db=core;g=AAEL018205) |  | 0.134508 | 0.4632 | 0.614315 | 1.110426 |
| [AAEL018211](https://www.vectorbase.org/aedes_aegypti/Gene/Summary?db=core;g=AAEL018211) |  | 0.462203 | 0.849315 | 0.901264 | 1.053192 |
| [AAEL018241](https://www.vectorbase.org/aedes_aegypti/Gene/Summary?db=core;g=AAEL018241) |  | 0.60004 | 1.05123 | 1.353205 | 1.894435 |
| [AAEL018245](https://www.vectorbase.org/aedes_aegypti/Gene/Summary?db=core;g=AAEL018245) |  | 0.119778 | 0.096276 | 0.022096 | 0.893879 |
| [AAEL018299](https://www.vectorbase.org/aedes_aegypti/Gene/Summary?db=core;g=AAEL018299) |  | 0.035954 | 0.181333 | 0.599905 | 0.977901 |
| [AAEL018301](https://www.vectorbase.org/aedes_aegypti/Gene/Summary?db=core;g=AAEL018301) |  | 0.130112 | 0.296632 | 0.69786 | 0.874948 |
| [AAEL018349](https://www.vectorbase.org/aedes_aegypti/Gene/Summary?db=core;g=AAEL018349) | cecropin, anti-microbial peptide [Source:VB Community Annotation;Acc:AAEL018349] | -0.57286 | -0.06959 | 0.039142 | 3.063062 |
| [AAEL018351](https://www.vectorbase.org/aedes_aegypti/Gene/Summary?db=core;g=AAEL018351) |  | 0.054565 | 0.274771 | 0.375823 | 0.888606 |
| [AAEL018354](https://www.vectorbase.org/aedes_aegypti/Gene/Summary?db=core;g=AAEL018354) | oxysterol-binding protein [Source:VB Community Annotation;Acc:AAEL018354] | -0.06166 | 0.476734 | 0.412226 | 0.9152 |
| Up regulated, strong  (cluster D) |  |  |  |  |  |
| [AAEL000393](https://www.vectorbase.org/aedes_aegypti/Gene/Summary?db=core;g=AAEL000393) | suppressors of cytokine signalling [Source:VB External Description;Acc:AAEL000393] | 0.231205 | 0.959896 | 1.802994 | 2.673787 |
| [AAEL000400](https://www.vectorbase.org/aedes_aegypti/Gene/Summary?db=core;g=AAEL000400) |  | 0.37197 | 1.048188 | 1.742548 | 2.635038 |
| [AAEL000566](https://www.vectorbase.org/aedes_aegypti/Gene/Summary?db=core;g=AAEL000566) |  | 0.575766 | 1.889668 | 2.016349 | 4.217391 |
| [AAEL001492](https://www.vectorbase.org/aedes_aegypti/Gene/Summary?db=core;g=AAEL001492) | cgmp-dependent 3,5-cyclic phosphodiesterase [Source:VB External Description;Acc:AAEL001492] | 0.374801 | 1.069658 | 1.627488 | 2.387877 |
| [AAEL001523](https://www.vectorbase.org/aedes_aegypti/Gene/Summary?db=core;g=AAEL001523) | secretory Phospholipase A2, putative [Source:VB External Description;Acc:AAEL001523] | 0.387872 | 1.594735 | 2.563681 | 2.919094 |
| [AAEL001528](https://www.vectorbase.org/aedes_aegypti/Gene/Summary?db=core;g=AAEL001528) |  | 0.325654 | 1.260216 | 2.623418 | 2.923282 |
| [AAEL001969](https://www.vectorbase.org/aedes_aegypti/Gene/Summary?db=core;g=AAEL001969) | protein serine/threonine kinase, putative [Source:VB External Description;Acc:AAEL001969] | 0.373628 | 0.074658 | 1.155509 | 2.048284 |
| [AAEL002661](https://www.vectorbase.org/aedes_aegypti/Gene/Summary?db=core;g=AAEL002661) | matrix metalloproteinase [Source:VB External Description;Acc:AAEL002661] | 0.635146 | 0.972168 | 2.036681 | 4.389958 |
| [AAEL002853](https://www.vectorbase.org/aedes_aegypti/Gene/Summary?db=core;g=AAEL002853) | ccaat/enhancer binding protein [Source:VB External Description;Acc:AAEL002853] | 1.195917 | 2.150462 | 1.94056 | 2.617796 |
| [AAEL005561](https://www.vectorbase.org/aedes_aegypti/Gene/Summary?db=core;g=AAEL005561) | plasma membrane calcium-transporting ATPase 3 (pmca3) [Source:VB External Description;Acc:AAEL005561] | 0.141447 | 0.88431 | 1.780157 | 2.398027 |
| [AAEL005992](https://www.vectorbase.org/aedes_aegypti/Gene/Summary?db=core;g=AAEL005992) | adam (a disintegrin and metalloprotease) [Source:VB External Description;Acc:AAEL005992] | 0.383458 | 1.322365 | 2.205901 | 2.779671 |
| [AAEL006006](https://www.vectorbase.org/aedes_aegypti/Gene/Summary?db=core;g=AAEL006006) | swiprosin [Source:VB External Description;Acc:AAEL006006] | 0.028817 | 1.206113 | 1.850223 | 2.866335 |
| [AAEL006104](https://www.vectorbase.org/aedes_aegypti/Gene/Summary?db=core;g=AAEL006104) |  | 1.779026 | 4.217519 | 4.653844 | 4.394967 |
| [AAEL006533](https://www.vectorbase.org/aedes_aegypti/Gene/Summary?db=core;g=AAEL006533) | Ets domain-containing protein [Source:VB External Description;Acc:AAEL006533] | 0.909581 | 2.987815 | 4.963934 | 5.442214 |
| [AAEL007199](https://www.vectorbase.org/aedes_aegypti/Gene/Summary?db=core;g=AAEL007199) |  | 0.378392 | 1.007727 | 2.13477 | 2.985075 |
| [AAEL007206](https://www.vectorbase.org/aedes_aegypti/Gene/Summary?db=core;g=AAEL007206) |  | 0.424727 | 0.728672 | 2.206726 | 2.918264 |
| [AAEL007207](https://www.vectorbase.org/aedes_aegypti/Gene/Summary?db=core;g=AAEL007207) |  | 0.246671 | 0.319751 | 1.989508 | 2.700432 |
| [AAEL007704](https://www.vectorbase.org/aedes_aegypti/Gene/Summary?db=core;g=AAEL007704) | lipoma preferred partner/lpp [Source:VB External Description;Acc:AAEL007704] | 0.446725 | 1.50536 | 2.574553 | 3.062503 |
| [AAEL010076](https://www.vectorbase.org/aedes_aegypti/Gene/Summary?db=core;g=AAEL010076) |  | 0.502078 | 1.297405 | 2.820431 | 3.165278 |
| [AAEL010678](https://www.vectorbase.org/aedes_aegypti/Gene/Summary?db=core;g=AAEL010678) |  | 0.123577 | 1.392055 | 1.746229 | 2.38919 |
| [AAEL011397](https://www.vectorbase.org/aedes_aegypti/Gene/Summary?db=core;g=AAEL011397) |  | 0.451408 | 1.095324 | 2.304397 | 2.97216 |
| [AAEL012617](https://www.vectorbase.org/aedes_aegypti/Gene/Summary?db=core;g=AAEL012617) |  | 0.220275 | 0.387014 | 1.803038 | 2.506761 |
| [AAEL013345](https://www.vectorbase.org/aedes_aegypti/Gene/Summary?db=core;g=AAEL013345) | alphaA-crystallin, putative [Source:VB External Description;Acc:AAEL013345] | 0.351318 | 1.041607 | 2.371855 | 3.753264 |
| [AAEL013349](https://www.vectorbase.org/aedes_aegypti/Gene/Summary?db=core;g=AAEL013349) | lethal(2)essential for life protein, l2efl [Source:VB External Description;Acc:AAEL013349] | 1.015753 | 0.685873 | 1.980143 | 2.917902 |
| [AAEL013350](https://www.vectorbase.org/aedes_aegypti/Gene/Summary?db=core;g=AAEL013350) | heat shock protein 26kD, putative [Source:VB External Description;Acc:AAEL013350] | 0.767178 | 0.202582 | 1.785211 | 2.646362 |
| [AAEL017225](https://www.vectorbase.org/aedes_aegypti/Gene/Summary?db=core;g=AAEL017225) |  | 1.973979 | 3.155248 | 4.139914 | 4.332275 |
| [AAEL017514](https://www.vectorbase.org/aedes_aegypti/Gene/Summary?db=core;g=AAEL017514) |  | 2.390819 | 1.900236 | 4.629312 | 6.196688 |
| [AAEL017973](https://www.vectorbase.org/aedes_aegypti/Gene/Summary?db=core;g=AAEL017973) | heat shock protein HSP70 [Source:VB Community Annotation;Acc:AAEL017973] | 0.364613 | 1.329097 | 3.384552 | 4.411295 |
| [AAEL017974](https://www.vectorbase.org/aedes_aegypti/Gene/Summary?db=core;g=AAEL017974) | heat shock protein HSP70 [Source:VB Community Annotation;Acc:AAEL017974] | 0.691489 | 1.876675 | 3.976855 | 5.269457 |
| [AAEL017975](https://www.vectorbase.org/aedes_aegypti/Gene/Summary?db=core;g=AAEL017975) | heat shock protein HSP70 [Source:VB Community Annotation;Acc:AAEL017975] | 0.552452 | 1.559318 | 3.482145 | 4.498861 |
| [AAEL017976](https://www.vectorbase.org/aedes_aegypti/Gene/Summary?db=core;g=AAEL017976) | heat shock protein HSP70 [Source:VB Community Annotation;Acc:AAEL017976] | 0.70742 | 1.6859 | 3.913671 | 5.114229 |
| [AAEL017977](https://www.vectorbase.org/aedes_aegypti/Gene/Summary?db=core;g=AAEL017977) | heat shock protein HSP70 [Source:VB Community Annotation;Acc:AAEL017977] | -0.07556 | 0.804456 | 2.603493 | 3.331793 |
| [AAEL017979](https://www.vectorbase.org/aedes_aegypti/Gene/Summary?db=core;g=AAEL017979) | heat shock protein HSP70 [Source:VB Community Annotation;Acc:AAEL017979] | 1.864912 | 1.809738 | 4.489023 | 5.604148 |
| [AAEL017980](https://www.vectorbase.org/aedes_aegypti/Gene/Summary?db=core;g=AAEL017980) | heat shock protein HSP70 [Source:VB Community Annotation;Acc:AAEL017980] | 1.56657 | 1.677597 | 4.12756 | 4.888908 |
| [AAEL017981](https://www.vectorbase.org/aedes_aegypti/Gene/Summary?db=core;g=AAEL017981) | heat shock protein HSP70 [Source:VB Community Annotation;Acc:AAEL017981] | 0.404583 | 1.108658 | 2.874351 | 3.728047 |
| Not in clusters |  |  |  |  |  |
| AAEL008767 | serine protease [Source:VB External Description;Acc:AAEL008767] | 9.78278 | 4.629569 | -19.7408 | -4.72524 |
