# Supplementary material for: Transcriptional cellular responses in midgut tissue of Aedes aegypti larvae following intoxication with Cry11Aa toxin from Bacillus thuringiensis
Source: BMC Genomics. 2015 Dec 9;16:1042. doi: 10.1186/s12864-015-2240-7 (PMC4673840; doi:10.1186/s12864-015-2240-7)
Supplement: Additional file 3: Table S3. — List of differentially expressed genes in control (non-toxin exposed larvae) RNAseq data at 12 h. (DOCX 68 kb) [file 12864_2015_2240_MOESM3_ESM.docx]

**Table S3.** List of differentially expressed genes in control (non-toxin exposed larvae) RNAseq data at 12 hours

| Gene ID | Description | Regulation | Common with Cry11Aa exposure |
| --- | --- | --- | --- |
| AAEL003816 |  | Down regulated | Yes |
| AAEL003841 | defensin anti-microbial peptide | Down regulated | Yes |
| AAEL003821 |  | Down regulated | No |
| AAEL006704 | fibrinogen and fibronectin | Down regulated | No |
| AAEL007942 | fibrinogen and fibronectin | Down regulated | No |
| AAEL004223 | cecropin anti-microbial peptide | Down regulated | No |
| AAEL000566 |  | Down regulated | Yes |
| AAEL003588 | DNA-J/hsp40 | Down regulated | Yes |
| AAEL002309 | Thioredoxin Peroxidase | Down regulated | Yes |
| AAEL007060 | lipase | Up regulated | No |
| AAEL003201 | Carboxy/choline esterase Alpha Esterase | Up regulated | Yes |
| AAEL008312 |  | Up regulated | No |
| AAEL001418 |  | Up regulated | No |
