# Supplementary material for: Transcriptional cellular responses in midgut tissue of Aedes aegypti larvae following intoxication with Cry11Aa toxin from Bacillus thuringiensis
Source: BMC Genomics. 2015 Dec 9;16:1042. doi: 10.1186/s12864-015-2240-7 (PMC4673840; doi:10.1186/s12864-015-2240-7)
Supplement: Additional file 4: Table S4. — List of all Interpro domains found in differential expressed genes without biological process GO annotation. (DOCX 92 kb) [file 12864_2015_2240_MOESM4_ESM.docx]

**Table S4** . List of all Interpro domains found in differential expressed genes without biological process GO annotation

| Condition | InteproID | Description | Percent Rank |
| --- | --- | --- | --- |
| 6 downregulated | IPR007588 | Zync finger, FLYWCH-type | 1 |
| 6 h upregulated | IPR002018 | Carboxylesterase, typeB | 0.94 |
|  | IPR029058 | Alpha/Beta hydrolase fold | 1 |
| 9 h downregulated | IPR015880 | Zync finger, C2H2-like | 0.9 |
|  | IPR013087 | Zync finger C2H2-type/integrase DNA binding domain | 0.9 |
|  | IPR007087 | Zync finger, C2H2 | 0.9 |
| 9 h upregulated | IPR029048 | Heat shock protein 70kD, C-terminal domain | 0.966 |
|  | IPR029047 | Heat shock protein 70kD, peptide binding domain | 0.966 |
|  | IPR013126 | Heat shock protein 70 family | 0.966 |
|  | IPR018181 | Heat shock protein 70, conserved site | 0.966 |
|  | IPR029058 | Alpha/Beta hydrolase fold | 0.955 |
|  | IPR002018 | Carboxylesterase, typeB | 0.944 |
|  | IPR013783 | Immunoglobulin-like fold | 0.933 |
| 12 h downregulated | IPR015880 | Zync finger C2H2-like | 1 |
|  | IPR007087 | Zync finger, C2H2 | 0.996 |
|  | IPR013087 | Zync finger C2H2-type/integrase DNA binding domain | 0.992 |
|  | IPR027417 | P-loop containing nucleoside triphoshpate hydrolase | 0.988 |
|  | IPR011009 | Protein kinase-like domain | 0.977 |
|  | IPR016024 | Armadillo-type fold | 0.977 |
|  | IPR013083 | Zync finger, RING/FYVE/PHD-type | 0.977 |
|  | IPR011990 | Tetratricopeptide-like helical domain | 0.973 |
|  | IPR015943 | WD40/YVTN repeat-like-containing domain | 0.936 |
|  | IPR003593 | AAA+ ATPase domain | 0.936 |
|  | IPR013069 | BTB/POZ | 0.936 |
|  | IPR013026 | Tetratricopeptide repeat-containing domain | 0.936 |
|  | IPR011333 | BTB/POZ fold | 0.936 |
|  | IPR013783 | Immunoglobulin-like fold | 0.936 |
|  | IPR013525 | ABC-2 type transporter | 0.936 |
|  | IPR003439 | ABC transporter-like | 0.936 |
|  | IPR012934 | Zync finger, AD-type | 0.936 |
|  | IPR000210 | BTB/POZ-like | 0.936 |
| 12 h upregulated | IPR029058 | Alpha/Beta hydrolase fold | 1 |
|  | IPR008978 | HSP20-like chaperone | 0.997 |
|  | IPR002068 | Alpha crystallin/Hsp20 domain | 0.991 |
|  | IPR001436 | Alpha crystallin/Heat shock protein | 0.991 |
|  | IPR027417 | P-loop containing nucleoside triphoshpate hydrolase | 0.988 |
|  | IPR029047 | Heat shock protein 70kD, peptide-binding domain | 0.969 |
|  | IPR013126 | Heat shock protein 70 family | 0.969 |
|  | IPR029048 | Heat shock protein 70kD, C-terminal domain | 0.969 |
|  | IPR001611 | Leucine-rich repeat | 0.969 |
|  | IPR011992 | EF-hand domain pair | 0.969 |
|  | IPR002048 | EF-hand domain | 0.969 |
|  | IPR018181 | Heat shock protein 70, conserved site | 0.969 |
|  | IPR013783 | Immunoglobulin-like fold | 0.958 |
|  | IPR002018 | Carboxylesterase, typeB | 0.958 |
|  | IPR018247 | EF-Hand 1, calcium-binding site | 0.958 |
|  | IPR011993 | Pleckstrin homology-like domain | 0.958 |
|  | IPR001849 | Pleckstrin homology domain | 0.947 |
|  | IPR020683 | Ankyrin repeat-containing domain | 0.947 |
|  | IPR013083 | Zync finger, RING/FYVE/PHD-type | 0.947 |
|  | IPR015880 | Zync finger C2H2-like | 0.947 |
|  | IPR015943 | WD40/YVTN repeat-like-containing domain | 0.925 |
|  | IPR002110 | Ankyrin repeat | 0.925 |
|  | IPR007110 | Immunoglobulin-like domain | 0.925 |
|  | IPR017986 | WD40-repeat-containing domain | 0.925 |
|  | IPR001680 | WD40 repeat | 0.925 |
|  | IPR001452 | SH3 domain | 0.925 |
|  | IPR008985 | Concanavalin A-like lectin/glucanases superfamily | 0.925 |
|  | IPR018502 | Annexin repeat | 0.925 |
|  | IPR003591 | Leucin-rich repeat, typical subtype | 0.903 |
|  | IPR001715 | Calponin homology domain | 0.903 |
|  | IPR018252 | Annexin repeat, conserved site | 0.903 |
|  | IPR000504 | RNA recognition motif domain | 0.903 |
|  | IPR013320 | Concanavalin A-like lectin/glucanase domain | 0.903 |
|  | IPR012677 | Nucleotide-binding alpha-beta plait domain | 0.903 |
|  | IPR007087 | Zync finger, C2H2 | 0.903 |
|  | IPR001464 | Annexin | 0.903 |
