# Supplementary figures and images for: Transcriptional cellular responses in midgut tissue of Aedes aegypti larvae following intoxication with Cry11Aa toxin from Bacillus thuringiensis
Source: BMC Genomics. 2015 Dec 9;16:1042. doi: 10.1186/s12864-015-2240-7 (PMC4673840; doi:10.1186/s12864-015-2240-7)

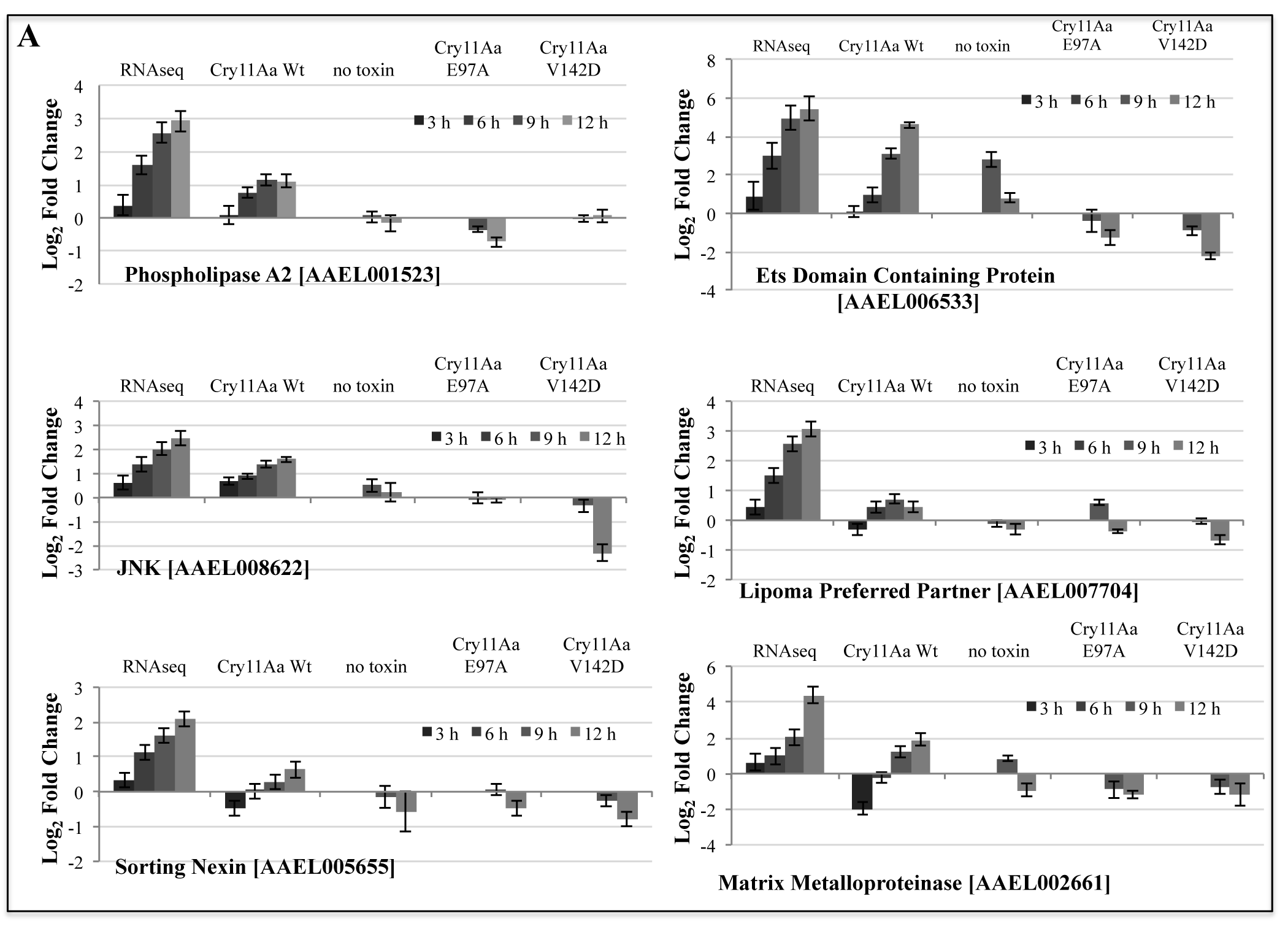

Supplement: Additional file 5: Figure S1. — RNAseq and log2 transformed RT-qPCR fold changes. Data is presented for RNAseq and RT-qPCR at 3, 6, 9, and 12 h of an LC50 Cry11Aa treatment. Also shown are RT-qPCR values for 9 and 12 h of unexposed larvae or Cry11Aa non-toxic mutants treatments. All log2 fold changes are referred to control larvae at the start of respective treatment. Panel A and B show genes with high correlation between RNAseq data and RT-qPCR of Cry11Aa. Panel C shows the three genes with low or no positive correlation between these data. (ZIP 779 kb) [file 12864_2015_2240_MOESM5_ESM.zip › figS1A.tif]

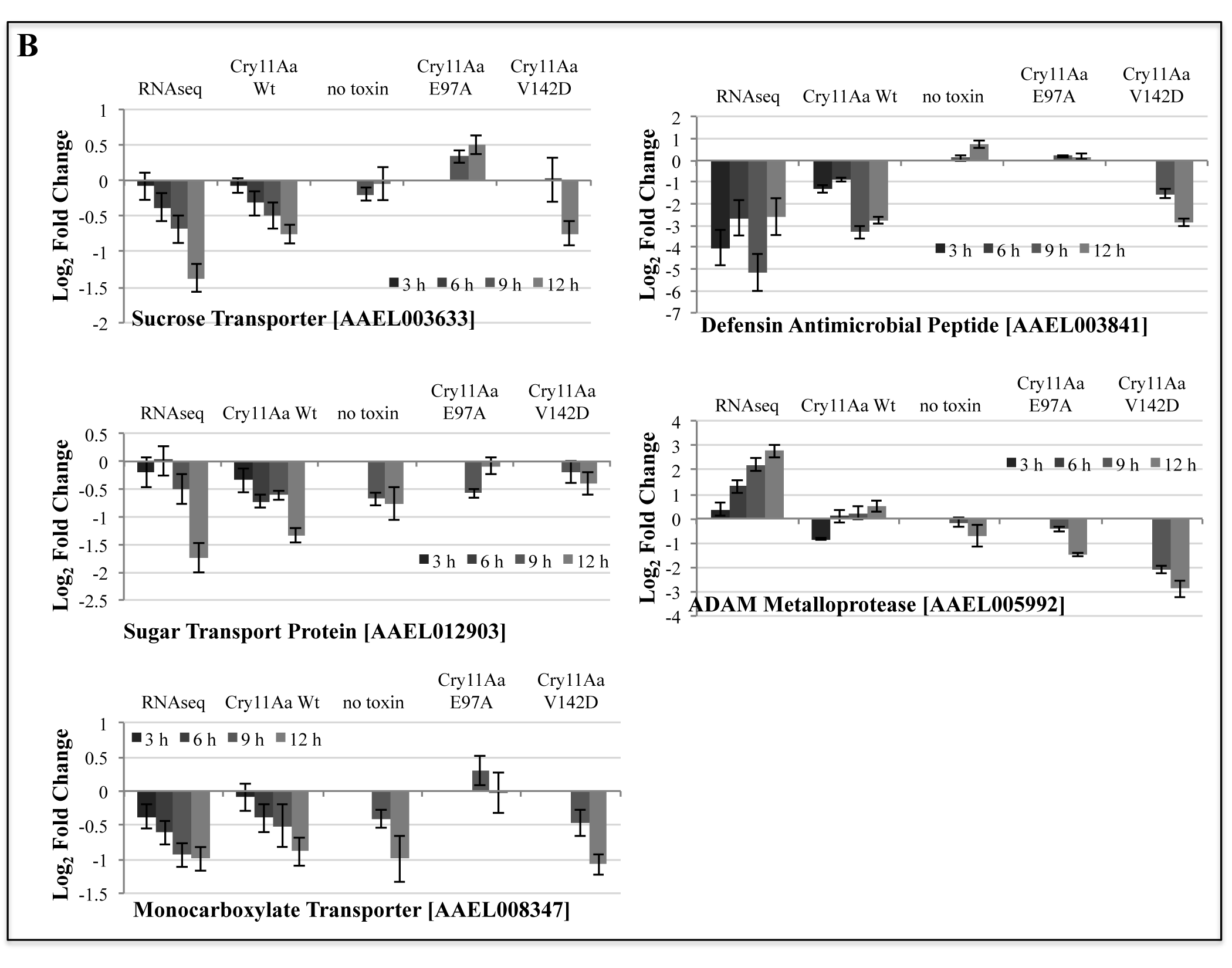

Supplement: Additional file 5: Figure S1. — RNAseq and log2 transformed RT-qPCR fold changes. Data is presented for RNAseq and RT-qPCR at 3, 6, 9, and 12 h of an LC50 Cry11Aa treatment. Also shown are RT-qPCR values for 9 and 12 h of unexposed larvae or Cry11Aa non-toxic mutants treatments. All log2 fold changes are referred to control larvae at the start of respective treatment. Panel A and B show genes with high correlation between RNAseq data and RT-qPCR of Cry11Aa. Panel C shows the three genes with low or no positive correlation between these data. (ZIP 779 kb) [file 12864_2015_2240_MOESM5_ESM.zip › figS1-B.tif]

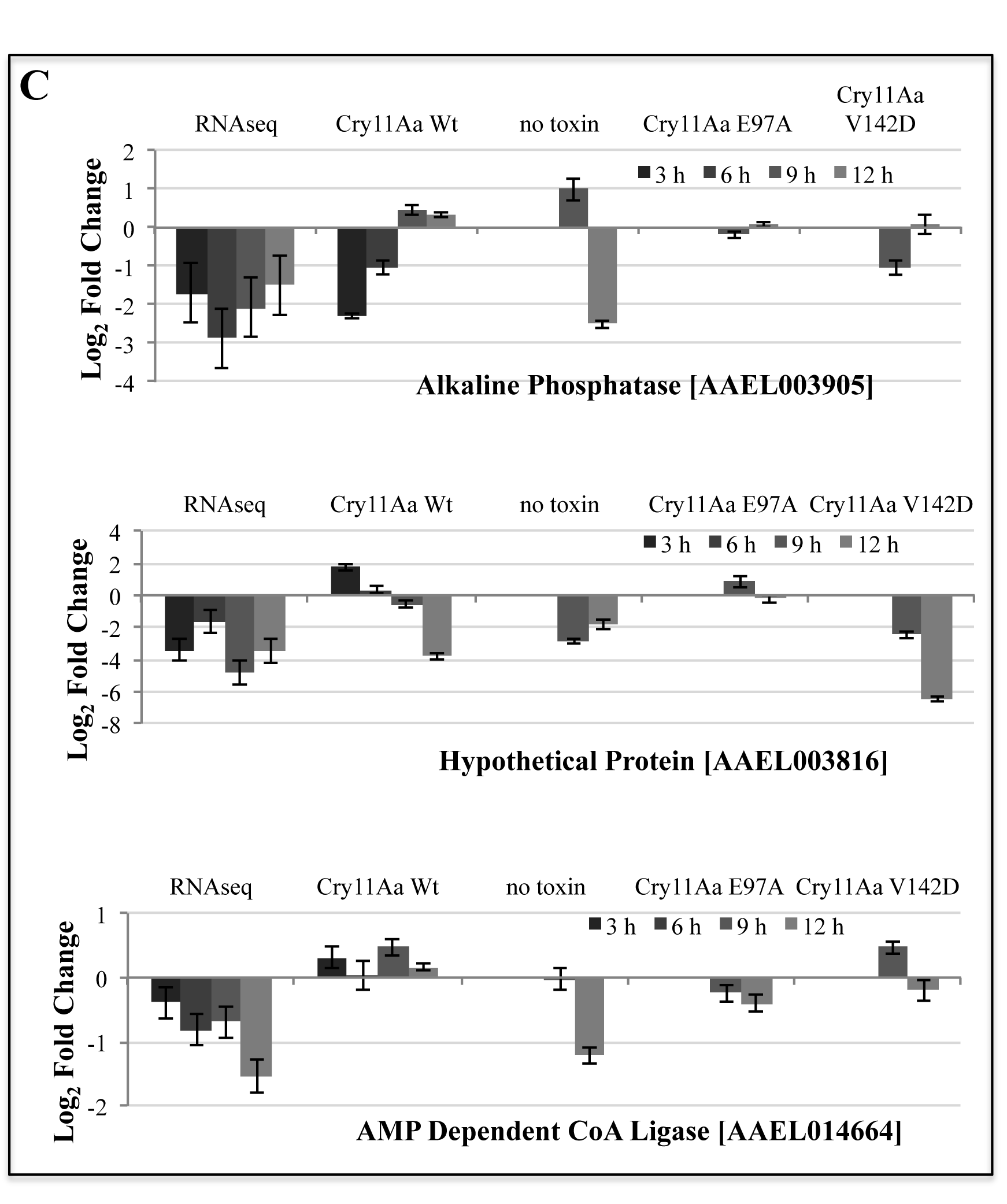

Supplement: Additional file 5: Figure S1. — RNAseq and log2 transformed RT-qPCR fold changes. Data is presented for RNAseq and RT-qPCR at 3, 6, 9, and 12 h of an LC50 Cry11Aa treatment. Also shown are RT-qPCR values for 9 and 12 h of unexposed larvae or Cry11Aa non-toxic mutants treatments. All log2 fold changes are referred to control larvae at the start of respective treatment. Panel A and B show genes with high correlation between RNAseq data and RT-qPCR of Cry11Aa. Panel C shows the three genes with low or no positive correlation between these data. (ZIP 779 kb) [file 12864_2015_2240_MOESM5_ESM.zip › figS1-C.tif]

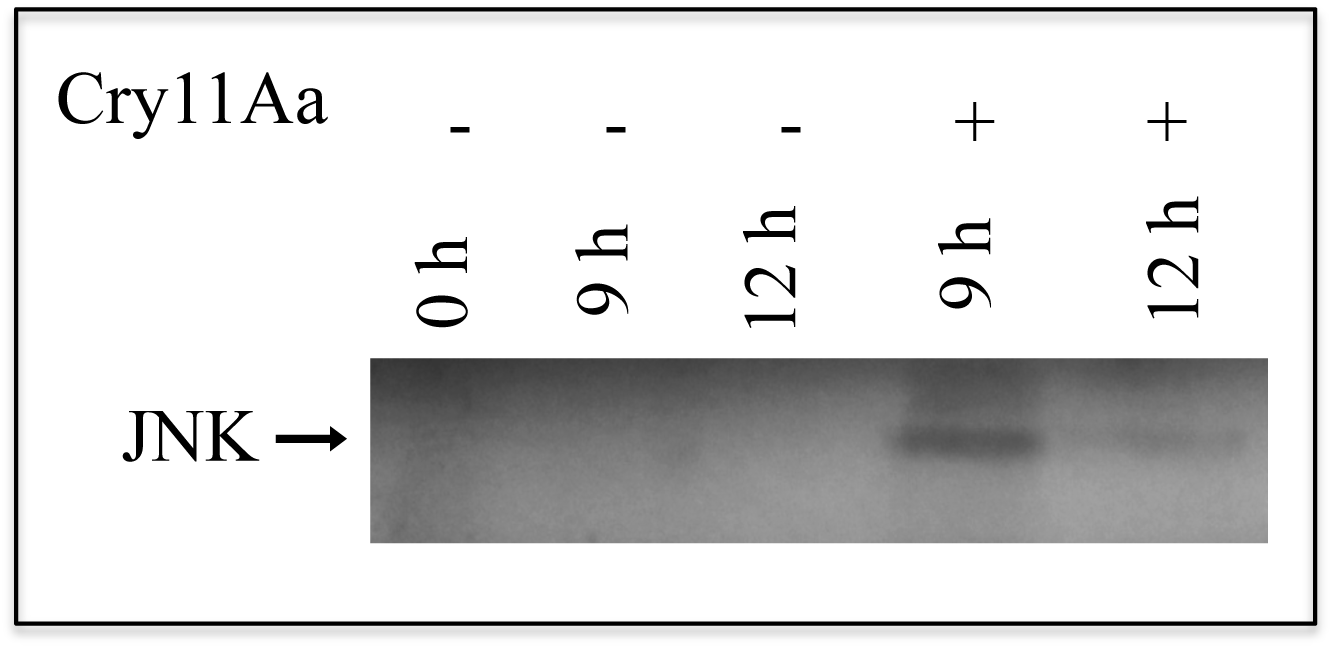

Supplement: Additional file 6: Figure S2. — Characterization of JNK expression by Western Blot analysis using α-JNK antibody. Presence of JNK was determined on protein extracts of midguts of A. aegypti larvae exposed to Cry11Aa for 9 or 12 h (lanes 4 and 5, respectively). Proteins were separated by SDS-PAGE and transferred to PVDF membranes for Western Blotting with human αJNK2 antibody. Non-toxin exposed larvae dissected at corresponding times were used as controls (lanes 1 to 3). (TIF 1042 kb) [file 12864_2015_2240_MOESM6_ESM.tif]
